# (±)-Japonones A and B, two pairs of new enantiomers with anti-KSHV activities from *Hypericum japonicum*

**DOI:** 10.1038/srep27588

**Published:** 2016-06-08

**Authors:** Linzhen Hu, Hucheng Zhu, Lei Li, Jinfeng Huang, Weiguang Sun, Junjun Liu, Hua Li, Zengwei Luo, Jianping Wang, Yongbo Xue, Yu Zhang, Yonghui Zhang

**Affiliations:** 1 Hubei Key Laboratory of Natural Medicinal Chemistry and Resource Evaluation, School of Pharmacy, Tongji Medical College, Huazhong University of Science and Technology, Wuhan 430030, China; 2 Union Hospital, Tongji Medical College, Huazhong University of Science and Technology, Wuhan 430022, China

## Abstract

Two pairs of new enantiomers with unusual 5,5-spiroketal cores, termed (±)-japonones A and B [(±)-**1** and (±)-**2**], were obtained from *Hypericum japonicum* Thunb. The absolute configurations of (±)-**1** and (±)-**2** were characterized by extensive analyses of spectroscopic data and calculated electronic circular dichroism (ECD) spectra, the application of modified Mosher’s methods, and the assistance of quantum chemical predictions (QCP) of ^13^C NMR chemical shifts. Among these metabolites, (+)-**1** exhibited some inhibitory activity on Kaposi’s sarcoma associated herpesvirus (KSHV). Virtual screening of (±)-**1** and (±)-**2** were conducted using the Surflex-Dock module in the Sybyl software, and (+)-**1** exhibited ability to bind with ERK to form key interactions with residues Lys52, Pro56, Ile101, Asp165, Gly167 and Val99.

*Hypericum japonicum* Thunb, an annual herbaceous plant of the genus *Hypericum* (Hypericaceae), widely distributed in Asia, Oceania, and North America, has been historically used for the treatment of hepatitis, tumors, and gastrointestinal disorder in Chinese traditional medicine^1^,^2^. Previous studies on this plant have revealed the presence of flavonoids, xanthonoids, chromone glycosides, phloroglucinol derivatives and lactones, and many of these secondary metabolites exhibit versatile pharmacological activities^3^,^4^,^5^,^6^,^7^.

In our continuous investigations of structurally unique and biologically active organic substances from the genus *Hypericum*, a substantial number of phloroglucinol derivatives were obtained from *H. sampsonii*, *H. ascyron*, and *H*. *attenuatum*^8^,^9^,^10^. Currently, our sustained interest in this genus led to the discovery of two pairs of new 1,6-dioxaspiro[4.4]non-2-en-4-one enantiomers (*i.e.*, (±)-japonones A and B) (Fig. 1) from the aerial parts of *H. japonicum*. Among these enantiomers, (+)-japonone A [(+)-**1**] exhibited some inhibitory activity towards KSHV lytic replication. The isolation, structural elucidation, and bioactivity screening are elaborated in the accompanying paper. A hypothetical biosynthetic pathway for (±)-japonones A and B has also been proposed.

## Results and Discussion

### Isolation and structure elucidation

The aerial parts of *H. japonicum* (30 kg) were dried naturally and immersed in 95% EtOH for three weeks at 25 °C to afford a brown syrup (0.75 kg), which was successively extracted with petroleum ether and chloroform against water. The petroleum ether extract (300 g) was subjected to flash chromatography on a silica gel column, RP-18 medium pressure liquid chromatography, semipreparative High Performance Liquid Chromatography (HPLC), and CHIRALPAKIC preparative column to furnish compounds (+)**-1** (3.1 mg), (−)**-1** (2.9 mg), (+)**-2** (3.0 mg) and (−)**-2** (2.7 mg).

(±)-Japonone A [(±)-**1**] was isolated via enantioseparation procedure into a pair of enantiomers with 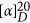 + 33.0 (*c* 0.03, MeOH) and 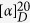 −32.6 (*c* 0.03, MeOH), respectively. The molecular formula of C_17_H_26_O_4_, which corresponds to 5 degrees of unsaturation, was indicated by the high-resolution electrospray ionisation mass spectrometry (HR-ESI-MS) data (*m/z* 317.1721 [M + Na]^+^) and ^13^C NMR data. The UV spectra displayed an absorption maximum at 272 nm. The IR spectra exhibited characteristic absorptions for the hydroxyl (3421 cm^−1^) and *α*,*β*-unsaturated carbonyl (1699 cm^−1^) functionalities. The ^1^H and ^13^C NMR spectra (Table 1) of **1** displayed signals corresponding to two olefinic protons, two methyl doublets, and three methyl singlets as well as signals of 17 carbon atoms, which involved five quaternary carbon atoms (containing one olefinic, one carbonyl, and one oxygenated carbon atom), four methines (*i.e.*, two olefinic, one oxygenated, and one aliphatic methine), three methylenes, and five methyls.

**Figure 2:**
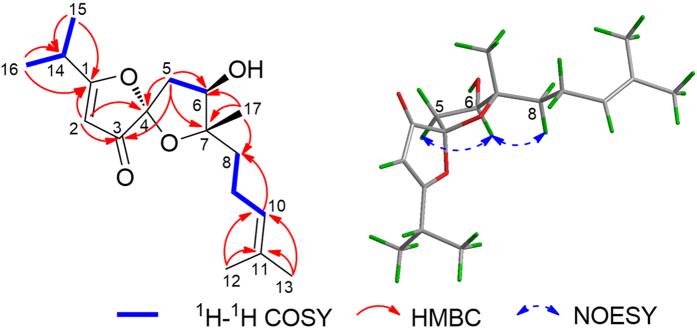
Key 2D NMR correlations for 1.

The structural connectivity of **1** was established by analyses of its ^1^H − ^1^H COSY and HMBC spectra aided by its HSQC spectrum (Fig. 2). The HMBC correlations from H-2 to C-1, C-3, and C-4, as well as the characteristic carbon chemical shifts of C-1 (*δ*_C_ 200.9), C-2 (*δ*_C_ 99.3), C-3 (*δ*_C_ 203.3), and C-4 (*δ*_C_ 112.1) suggested the presence of a *β*-*O*-substituted *α*,*β*-unsaturated ketone moiety^11^, which is consistent with the UV maximum at 272 nm. Furthermore, the HMBC cross peaks from H-5 to C-3, C-4, C-6, and C-7 along with the ^1^H − ^1^H COSY spin system of H-5/H-6 as well as the chemical shift of C-7 (*δ*_C_ 92.6) indicated a 1,6-dioxaspiro[4.4]non-2-en-4-one skeleton, as shown in Fig. 2. The additional HMBC correlations from Me-12 and Me-13 to C-10 and C-11 along with the ^1^H − ^1^H COSY cross peaks of H-8/H-9/H-10 suggested the presence of an isoprenylmethyl group, which was located at C-7 based on the HMBC correlations from Me-17 to C-6, C-7, and C-8. In addition, an isopropyl group attached to C-1 was confirmed via the HMBC signals from Me-15 and Me-16 to C-1 and C-14.

To unravel the relative stereochemistry of **1**, the key NOE correlations were carefully analyzed and illustrated as shown in Fig. 2. The NOE correlation between H-5*α*/H-6 and H-6/H-8 suggested the cofacial location of these protons, while no NOE correlation was observed between H-17 and H-6. Thus, OH-6 and Me-17 are located on the same side of the 5-membered ring plane. However, the relative configuration of C-4 could not be determined by NOESY spectrum due to the lack of diagnostic signal.

**Figure 3:**
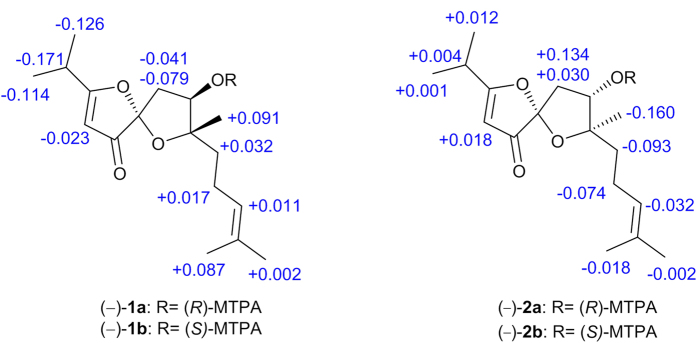
∆δ values (in ppm) = *δ*_S-MTPA-ester_ − *δ*_R-MTPA-ester_ for (−)-**1a**/(−)-**1b** and (−)-**2a**/(−)-**2b**, respectively.

**Figure 4:**
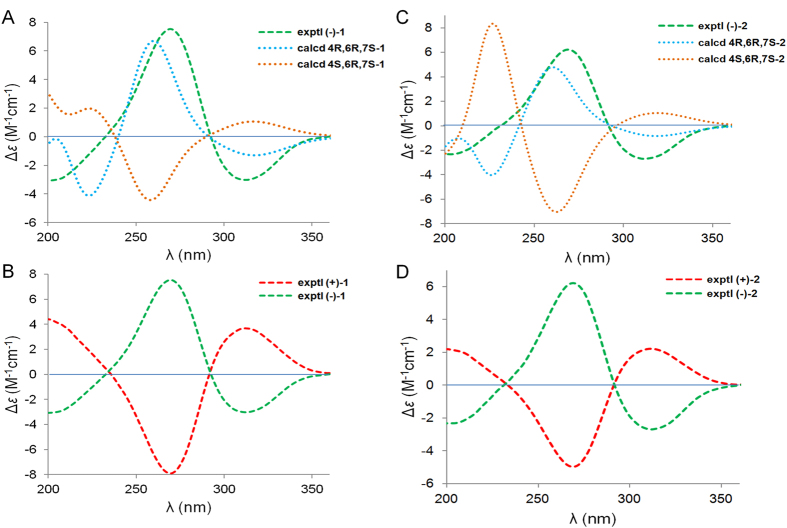
Experimental and calculated ECD spectra.

The absolute configuration of C-6 in (−)-**1** was validated using a modified Mosher’s experiment^12^,^13^. The prepared (*S*)- and (*R*)-MTPA esters of (−)-**1** were subjected to ^1^H NMR analysis, and the distinct values of the ^1^H NMR chemical shifts (*∆δ* = *δ*_*S*-MTPA-ester_ − *δ*_*R*-MTPA-ester_) were summarized for the proton signals adjacent to C-6, as shown in Fig. 3. Based on these results, the absolute configuration of C-6 was confirmed to be *R*. Based on analyses of the NOE experimental results, the chiral characteristic of C-7 was unambiguously confirmed to be *S*. To determine the absolute configuration of C-4, the time-dependent density functional theory (TD-DFT) ECD calculations were carried out for 4*R*,6*R*,7*S*-**1** and 4*S*,6*R*,7*S*-**1**, respectively (Fig. 4A). According to the ECD spectroscopic calculations, the absolute configurations of C-4 in (−)-**1** was established to be *R*. Therefore, the absolute configuation of (−)-**1** was determined to be 4*R*,6*R*,7*S*, and accordingly, the absolute configuration of (+)-**1** was 4*S*,6*S*,7*R* (Fig. 4B).

**Figure 5:**
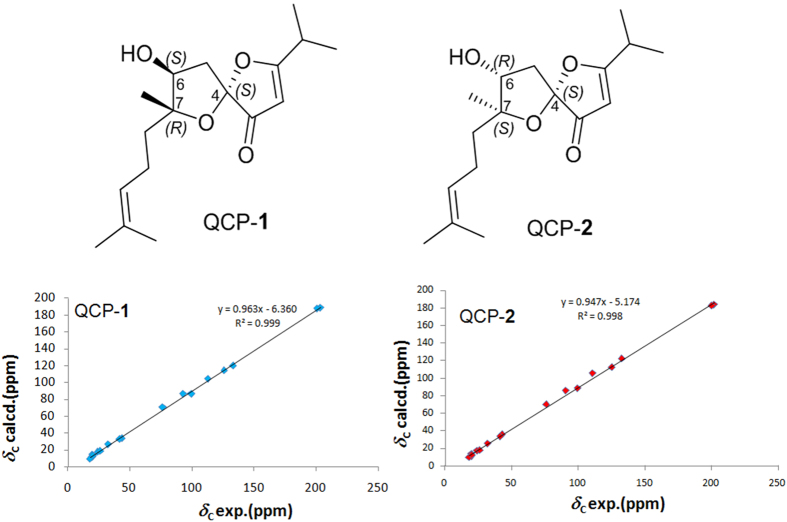
Configurations of QCP-1 and QCP-2. Linear correlations between the scaled calculated and experimental ^13^C NMR chemical shifts for QCP-1 and QCP-2.

In addition, a quantum chemical prediction (QCP) of the ^13^C NMR data for (+)-**1** (4*S*,6*S*,7*R*) (QCP-**1**, Fig. 5) was performed. A linear correlation between the calculated ^13^C NMR chemical shifts acquired from QCP and the experimental shifts was constructed (Fig. 5) to obtain scaled calculated data to establish the maximum absolute deviations (MaxDev) and the average absolute deviations (AveDev) (Table 2). The MaxDev and AveDev revealed that the experimental data matched well with those of predicted data, which further confirmed the structure of (+)-**1**.

(±)-Japonone B [(±)-**2**] (C_17_H_26_O_4_, HR-ESI-MS [M + Na]^+^ *m/z* 317.2721, 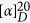 +21.0 (*c* 0.07, MeOH) and 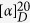 −22.0 (*c* 0.08, MeOH)), which is an enantiomorph pair, was isolated via an enantioseparation procedure that was identical to that used for (±)-**1**. The UV and IR spectral data of **2** were similar to those of **1**, as shown in the Supplementary Information (SI). Additionally, the ^1^H and ^13^C NMR spectra of **2** closely resembled those of **1** with very slight shifts (Table 1). The above analyses indicated **1** and **2** possess homologous structures, which was further defined by its HMBC and ^1^H − ^1^HCOSY spectra (for details see Figure S1, SI). The relative configurations of C-6 and C-7 were determined to be the same as those of **1** based on a NOESY experiment, and the absolute configuration of C-6 in (−)-**2** was determined to be *S* by the application of the modified Mosher’s method (Fig. 3). Thus, (−)-**2** was speculated to be a C-4 epimer of (+)-**1**. Eventually, the absolute configuration of (−)-**2** was confirmed to be 4*R*,6*S*,7*R* by the analyses of ECD calculations of 4*R*,6*S*,7*R*-**2** and 4*S*,6*S*,7*R*-**2** (Fig. 4C). Consequently, the absolute configuration of (+)-**2** was established to be 4*S*,6*R*,7*S* (Fig. 4D). The structure of (+)-**2** was also secured by predicted ^13^C NMR data in the same manner as that of (+)-**1** (QCP-2, Fig. 5).

**Figure 6:**
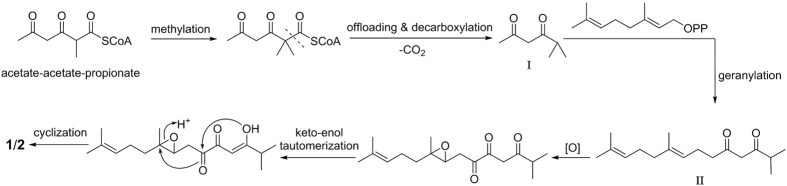
Plausible biogenetic pathway for 1/2.

The isolation of the two pairs of 1,6-dioxaspiro[4.4]non-2-en-4-one enantiomers ((±)-japonones A and B [(±)**-1** and (±)**-2**]) with intriguing dioxaspironone structures represents the first discovery from the *Hypericum* family. A plausible biogenetic formation pathway for **1** and **2** is shown in Fig. 6. Acetate-acetate-propionate is most likely an initial precursor in the formation of compounds **1** and **2**. First, this precursor might undergo methylation and decarboxylation to generate an intermediate material **I**. Then, geranyl-geranylation and oxidization of **I** may lead to the generation of the key intermediate **II**, which further endures a concerted intramolecular cyclization reaction to generate the 5,5-spiroketal core and formed the final structures of **1** and **2**.

### Anti-KSHV activity evaluation

Natural products have furnished new compounds/lead structures as momentous resources in exploration of front-line drugs^14^. However, drug discovery from natural-product still is a challenge because of insufficient knowledge on the biological targets of the numerous natural-product and technical limitations in distinguishing new compounds with desirable activities^15^. With the limited masses of metabolites (±)**-1** and (±)**-2**, inhibitory activities on *β*-site amyloid precursor protein cleaving enzyme 1 (BACE1), cytotoxic activities against five human cancer cell lines (i.e., HL-60, SMMC-7721, A-549, MCF-7, and SW480), and inhibitory activities towards NO production were assessed. The results showed that both (±)**-1** and (±)**-2** exhibited inefficacy with IC_50_ > 40 *μ*M for BACE1 inhibition and cytotoxicity assays, and with IC_50_ > 25 *μ*M for NO production inhibition assay, respectively. Nevertheless, in the anti-KSHV assay, it is interesting that as one of stereoisomers, (+)**-1** exhibited a better potency than the others’ with a higher selectivity index. The anti-infectivity assay on KSHV lytic replication was measured according to the previously published method^16^. The results revealed that compound (+)**-1** exhibited potency with considerably less toxicity and better selectivity (*i.e.*, EC_50_ 166.00 *μ*M and selectivity index higher than 3.01), respectively, while (−)**-1** and (±)**-2** exhibited inert activities on anti-KSHV (Table 3 and Figure S2).

### Inverse docking identifies ERK as a possible antiviral target

**Figure 7:**
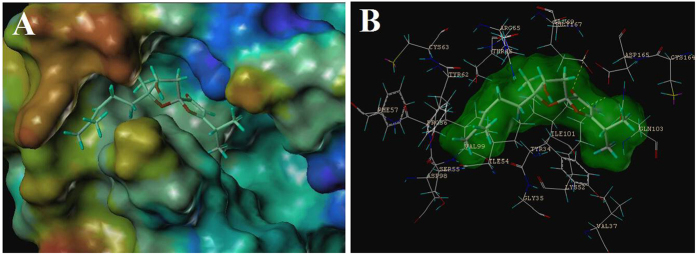
Binding poses of (+)-1 bound to ERK.

Based on the results of anti-KSHV activities, these compounds were subjected to further investigation to deduce the hypothetic anti-KSHV mechanisms. Six KSHV therapeutic targets including KSHV protease, KSHV LANA, PKC, P38, JNK, and ERK were used in virtual screening as implemented in the the Surflex-Dock module of the Sybyl software. Taking Total-Score as the standard of the scoring function, the interactions between the targets and the molecules were assessed. The calculated results predicted that ERK exhibited better binding affinity with compound (+)**-1** (Total-Score value = 7.38, as shown in Table S1) than others. (+)**-1** had the ability to form key hydrophilic interactions with residues Lys52, Asp165, and Gly167. Furthermore, hydrophobic interactions with Pro56, Ile101, and Val99 were also observed (Fig. 7).

### Specific binding with ERK

The ability of the purified ERK to binding compounds (±)-**1** and (±)-**2** were tested using microscale thermophoresis (MST) analyses. As shown in Table S1, the K_D_ value of (+)-**1** [274 (±9.5) × 10^–6^ M] (Figure S3A) was lower than those of the other compounds (Figures S3B–D), which indicated a useful binding affinity between compound (+)-**1** with ERK.

In summary, the genus *Hypericum* possesses numerous compounds along with diversified biological activity^17^. Japonones A and B [(±)-**1** and (±)-**2**], which are two pairs of enantiomers with the unusual 5,5-spiroketal cores, were discovered for the first time from the genus *Hypericum*, This discovery greatly enriches the types of secondary metabolites from *Hypericum*. Structure determinations of these new metabolites were unequivocally resolved via extensive spectral analyses, ^13^C NMR and ECD calculations, and a modified Mosher’s method. Furthermore, compound (+)-**1** exhibited considerably less toxicity and better selectivity on anti-KSHV activity. Additionally, the equal amounts of isolated racemic mixtures (**1** and **2**) suggested their biosynthetic formation involving nonenzymatic steps. Their different efficacy against KSHV infection also provides a clue for the exploration of their potential structure−activity relationships.

## Methods

### Experimental procedures

Thin-layer chromatography (TLC) was conducted with HPTLC Silica gel 60 RP-18 F254s 25 Glass plates (Merck Millipore) and LuxPlate^®^ silica gel 60 F254 (Merck, Germany). Silica gel (120–200 mesh; Qingdao Bang-Kai High and New Technology Co., LTD., China), Sephadex LH-20 (Pharmacia, America), and RP-18 (50** ***μ*m, Merck, Germany) were used for column chromatography. HPLC experiments were subjected to LC3050 Analysis of HPLC system (CXTH, Beijing, China) equipped with an UV 3000 detector and a semi-preparative column (5** ***μ*m, 10 × 250 mm, Welch Ultimate^®^ XB-C_18_). Enantioseparation was achieved using a CHIRALPAKIC column (5** ***μ*m, 10 × 250 mm, Daicel Chiral Technologies Co., LTD., China). The HR-ESI-MS data were resolved in positive ion mode on a Thermo Scientific^TM^ LTQ Orbitrap XL^TM^ spectrometer. The UV and FT-IR spectra were recorded on a PerkinElmer Lambda 35 and Bruker Vertex 70 apparatus, respectively. A Hanon P810 automatic polarimeter was used to record the optical rotation values. The ECD spectra were measured on a JASCO J-1500 Spectrometer (JASCO, Japan). The NMR spectra were recorded on a Bruker AM-400/600 Spectrometer (Bruker, Switzerland) using tetramethylsilane (TMS) as an internal standard, and the ^1^H and ^13^C NMR data were normalized to the solvent peaks for methanol-*d*_4_ at *δ*_H_ 3.31 and *δ*_C_ 49.15.

### Plant material

The aerial parts of *H. japonicum* were harvested in October 2011 from Da-Bie Mountain, Qi-Chun County, Hubei Province, P. R. China and authenticated by Professor Jianping Wang. A voucher specimen (ID 20111011) has been preserved in the Herbarium Laboratory, School of Pharmacy, Tongji Medical College, Huazhong University of Science and Technology, P. R. China.

### Extraction and isolation

The aerial parts of *H. japonicum* (30 kg) were dried naturally and immersed in 95% EtOH for three weeks at 25 °C to afford a brown syrup (0.75 kg) under vacuum distillation, which was sequentially extracted with petroleum ether and chloroform against water. Based on TLC analyses, the petroleum ether extract (300 g) was partitioned into seven fractions (Fr.1–Fr.7) by silica gel column chromatography (CC) with gradient elution using petroleum ether-acetone (50:1 → 5:1). Next, Fr.5 was subjected further to silica gel CC to yield five subfractions (Fr.5.1–Fr.5.5). Based on TLC analyses, Fr.5.3 was selected to be repurified over MPLC (RP-18, 1.5 × 20 cm, MeOH-H_2_O, 40% → 80%) to furnish subfractions of Fr.5.3.1–Fr.5.3.5. Finally, Fr.5.3.2 was passed through Sephadex LH-20 (2 × 150 cm, CH_2_Cl_2_-MeOH), loaded on a silica gel CC eluted with CHCl_3_-MeOH 25/1, and exhaustively separated via semipreparative HPLC (MeOH-H_2_O 50%) to yield two pairs of racemates, *viz.*, (±)**-1** (7.0 mg) and (±)**-2** (6.2 mg).

### Semipreparative enantioseparation

**Figure 8:**
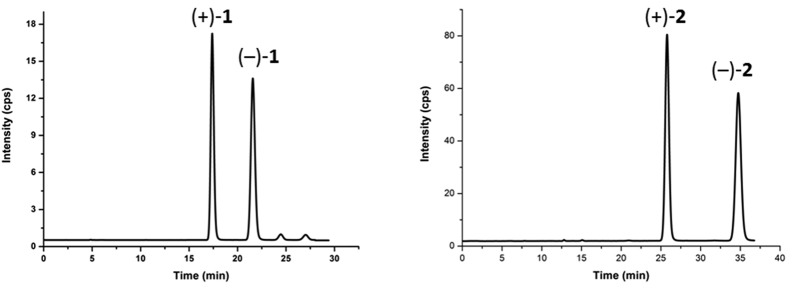
Chromatograms showing the enantioseparation of the two pairs of racemates (1 and 2).

To acquire the isolation of two pairs of racemic mixtures [(±)-**1** and (±)-**2**], analytical and semipreparative enantioseparations were achieved using chiral HPLC methods. The racemic resolution of (±)-**1** and (±)-**2** was performed using a CHIRALPAKIC preparative column (5** ***μ*m, 10 × 250 mm, Daicel Chiral Technologies Co., LTD., China). The separation chromatograms for these two racemates are shown in Fig. 8. The mass ratios of optical antipodes in the two isolated pairs of enantiomers were approximately of 1:1. Moreover, better than baseline separation was achieved for (±)-**1** and (±)-**2** from their racemic mixtures (Fig. 8). Hexane-isopropanol was used as the mobile phase at a flow rate of 3.0 mL/min, and a column temperature of 25 °C with UV detection at 270 nm were applied as the chromatographic conditions for the successful enantioseparation of (±)-**1** and (±)-**2**.

### Preparation of the (*S*)-MTPA and (*R*)-MTPA esters from (−)-1 and (−)-2

MTPA esters of (−)-**1** and (−)-**2** were prepared according to a previously described method^12^,^13^. A solution of (−)-**1** (0.79 mg) in anhydrous CH_2_Cl_2_ (2.0 mL) was treated with (*R*)-MTPA (24.3 mg) in the presence of dimethylaminopyridine (15 mg) and trimethylamine. Then, the mixture was agitated at room temperature under reflux for 3 h followed by quenching with the addition of 80** ***μ*L of anhydrous MeOH. This reaction mixture was condensed under vacuum evaporation to afford a residue, which was subjected to a small silica gel column [1.0 g, hexane-isopropanol (80:1 → 50:1), v/v)] to provide the (*S*)-MTPA ester of (−)-**1** [(−)-**1a**, 1.2 mg]. The (*R*)-MTPA derivative [(−)-**1b**, 1.3 mg] was obtained using (*S*)-MTPA chloride and chromatographed in the same manner. A similar procedure was applied to yield the MTPA esters of compounds (−)-**2** [(−)-**2a** and (−)-**2b**].

(±)*-Japonone A* [(±)-**1**]: UV (MeOH) *λ*_max_ (log *ε*) 272 (3.77) nm; IR (KBr) *v*_max_ 3446 cm^–1^, 2972 cm^–1^, 2914 cm^–1^, 1696 cm^–1^, and 1575 cm^–1^; ^1^H and ^13^C NMR data, see Table 1; positive HRESIMS: *m/z* 317.1721 [M + Na]^+^ (calcd for C_17_H_26_O_4_Na, 317.1729).

(+)-*Japonone A* [(+)-**1**], colorless oil, 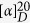 +33.0 (*c* 0.03, MeOH), ECD (*c* 3.40 × 10^−4^ M, MeOH) *λ*_max_ nm (*∆ε*) 270 (−8.07), 312 (+3.76);

(−)**-***Japonone A* [(−)-**1**], colorless oil, 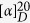 −32.6 (*c* 0.03, MeOH), ECD (*c* 3.00 × 10^−4^ M, MeOH) *λ*_max_ nm (*∆ε*) 270 (+7.53), 312 (−3.02).

(±)-*Japonone B* [(±)-**2**]: UV (MeOH) *λ*_max_ (log *ε*) 271 (3.88) nm; IR (KBr) *v*_max_ 3421 cm^–1^, 2972 cm^–1^, 2936 cm^–1^, 1696 cm^–1^, and 1575 cm^–1^; ^1^H and ^13^C NMR data, see Table 1; positive HRESIMS: *m/z* 317.1721 [M + Na]^+^ (calcd for C_17_H_26_O_4_Na, 317.1729).

(+)**-***Japonone B* [(+)-**2**], colorless oil, 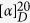 +21.0 (*c* 0.07, MeOH), ECD (*c* 3.24 × 10^−4^ M, MeOH) *λ*_max_ nm (*∆ε*) 269 (−4.84), 311 (+2.16);

(−)**-***Japonone B* [(−)-**2**], colorless oil, 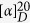 −22.0 (*c* 0.08, MeOH), ECD (*c* 4.03 × 10^−4^ M, MeOH) *λ*_max_ nm (*∆ε*) 269 (+6.54), 312 (−2.84).

### Compound (−)-1a

(*S*)-MTPA-ester: Amorphous powder; ^1^HNMR (CD_3_OD, 400 MHz) *δ*_H_: 7.571 − 7.548 (2H, m, aromatic protons), 7.481 − 7.446 (3H, m, aromatic protons), 5.417 (1H, s, H-2), 5.150 (1H, m, H-10), 3.565 (3H, br s, OMe), 2.755 (1H, dd, *J* = 15.2, 6.1 Hz, H-5b), 2.574 (1H, m, H-14), 2.191 (1H, d, *J* = 15.2 Hz, H-5a), 2.130 (2H, m, H-9), 1.676 (2H, m, H-8), 1.693 (3H, s, H-13), 1.644 (3H, s, H-12), 1.349 (3H, s, H-17), 1.133 (3H, d, *J* = 7.0 Hz, H-15), 1.115 (3H, d, *J* = 7.0 Hz, H-16); positive HRESIMS: *m/z* 533.2121 [M + Na]^+^ (calcd for C_27_H_33_F_3_O_6_Na, 533.2127).

### Compound (−)-1b

(*R*)-MTPA-ester: Amorphous powder; ^1^HNMR (CD_3_OD, 400 MHz) *δ*_H_: 7.545 − 7.521 (2H, m, aromatic protons), 7.480 − 7.443 (3H, m, aromatic protons), 5.440 (1H, s, H-2), 5.139 (1H, m, H-10), 3.537 (3H, br s, OMe), 2.796 (1H, d, *J* = 6.8 Hz, H-5b), 2.745 (1H, sept, *J* = 7.0 Hz, H-14), 2.270 (1H, dd, *J* = 14.3, 6.8 Hz, H-5a), 2.113 (2H, m, H-9), 1.691 (3H, s, H-13), 1.644 (2H, m, H-8), 1.557 (3H, s, H-12), 1.258 (3H, s, H-17), 1.247 (3H, d, *J* = 7.0 Hz, H-15), 1.241 (3H, d, *J* = 7.0 Hz, H-16); positive HRESIMS: *m/z* 533.2114 [M + Na]^+^ (calcd for C_27_H_33_F_3_O_6_Na, 533.2127).

### Compound (−)-2a

(*S*)-MTPA-ester: Amorphous powder; ^1^HNMR(CD_3_OD, 400 MHz) *δ*_H_: 7.593 − 7.570 (2H, m, aromatic protons), 7.463 − 7.447 (3H, m, aromatic protons), 5.458 (1H, s, H-2), 5.108 (1H, m, H-10), 3.612 (3H, br s, OMe), 2.830 (1H, dd, *J* = 14.7, 7.0 Hz, H-5b), 2.764 (1H, sept, *J* = 6.7 Hz, H-14), 2.400 (1H, d, *J* = 14.7, 6.3 Hz, H-5a), 2.039 (2H, m, H-9), 1.650 (2H, m, H-8), 1.689 (3H, s, H-13), 1.615 (3H, s, H-12), 1.260 (3H, overlapped, H-15), 1.242 (3H, overlapped, H-16), 1.099 (3H, s, H-17); positive HRESIMS: *m/z* 533.2113 [M + Na]^+^ (calcd for C_27_H_33_F_3_O_6_Na, 533.2127).

### Compound (−)-2b

(*R*)-MTPA-ester: Amorphous powder; ^1^HNMR(CD_3_OD, 400 MHz) *δ*_H_: 7.545 − 7.521 (2H, m, aromatic protons), 7.471 − 7.457 (3H, m, aromatic protons), 5.440 (1H, s, H-2), 5.140 (1H, m, H-10), 3.537 (3H, br s, OMe), 2.800 (1H, d, *J* = 6.6 Hz, H-5b), 2.760 (1H, sept, *J* = 6.7 Hz, H-14), 2.266 (1H, d, *J* = 6.6 Hz, H-5a), 2.113 (2H, m, H-9), 1.743 (2H, m, H-8), 1.691 (3H, s, H-13), 1.633 (3H, s, H-12), 1.259 (3H, s, H-17), 1.248 (3H, overlapped, H-15), 1.241 (3H, overlapped, H-16); positive HRESIMS: *m*/*z* 533.2113 [M + Na]^+^ (calcd for C_27_H_33_F_3_O_6_Na, 533.2127).

### Anti-KSHV assays

Anti-KSHV assays including cytotoxicity and anti-KSHV infectivity assays were performed. Human iSLK.219 cells were employed in order to assess the antiviral activity of the desired compounds against KSHV. The rKSHV.219 virus bearing green fluorescent protein (GFP) under regulation of the elongation factor 1*α* (EF-1*α*) promoter was recombined into the iSLK.219 cells. 1.2 mM sodium butyrate (NaB) (Aladdin Industrial Corporation) and 1 *μ*g/mL doxycycline (Dox) (Aladdin Industrial Corporation) were used to promote the lytic replication of KSHV^18^,^19^. The iSLK.219 cells grown to 80% confluence within 96-well plates were accurately added with the desired concentrations of the compounds treated with Dox and NaB. Then, the cell viability was measured after 48 h using the AlamarBlue^®^ Cell Viability Assay (Thermo Fisher Scientific™, Waltham, Massachusetts, USA). The luminescent expression was recorded using the PerkinElmer Multilabel Reader (Waltham, MA, USA). The 50% cytotoxic concentration (CC_50_) of the compounds was obtained using mathematical statistics via Graphpad5.0 Prism. The results are shown in Figure S2 (mean values with standard deviations, n = 3).

According to previous studies, the infectivity assays were carried out to determine the anti-KSHV activity of the compounds^16^. The iSLK.219 cells that were treated or untreated with the compounds in the presence of Dox and NaB supernatants were incubated for 48 h. Then, the supernatants were collected and used to infect the Vero cells cultivated in a 96-well plate, followed by centrifugation at 1,500 × *g* for 60 min^20^. Then, the supernatants were removed and superseded with fresh Dulbecco’s Modified Eagle Medium (DMEM). At 48 h, fluorescence detection and quantitative analyses were performed using a High-Content Screening System (HCS) (PerkinElmer) to measure the expressions of GFP per well in the Vero cells. Nine image fields per well were analyzed using an automatic microscope based HCS, and the GFP signals per well was determined using the Harmony 3.5 software (PerkinElmer). The data were normalized as the fold change compared to the DMSO control. The 50% effective concentration (EC_50_) corresponded to each concentration of compound that offered a 50% reduction in the quantitative expression of the intensity of GFP. The results are shown in Figure S2 (mean values with standard deviations, n = 3).

### Docking simulation analyses

To predict the potential binding targets of compounds (±)-**1** and (±)-**2** and illustrate the accurate binding model and mechanism of interaction, molecular docking analyses were performed with Surflex-Dock as implemented in the SYBYL-X 2.0 program package (Tripos International, St. Louis, MO, USA)^21^,^22^. The crystal structures of the docking targets including KSHV protease, KSHV LANA, PKC, P38, JNK, and ERK were obtained from the Protein Data Bank (http://www.rcsb.org)^23^,^24^,^25^,^26^,^27^,^28^. Prior to the docking simulations, the structures of the compounds were visualized using ChemOffice 12.0 (CambridgeSoft), and the minimum energy conformation of each compound was determined using the standard Tripos molecular mechanics force field in the SYBYL-X 2.0 molecular modelling package. For the docking experiment, the default parameters and docking mode of Surflex-Dock GeomX were applied to acquire 30 conformations for each structure. The conformation of the maximum total score was adopted for further investigation.

### Binding affinity using microscale thermophoresis (MST)

MST was applied to evaluate the binding interactions between recombined ERK and compounds (±)-**1** and (±)-**2** using a setup similar to that previously described^29^,^30^. The protein was labelled with the Monolith NT™ Protein Labelling Kit RED (Cat#L001) according to the supplied labelling protocol. The labelled ERK was maintained constant at 100 nM, and all of the tested samples were diluted in 20 mM HEPES (pH 7.5), 5% DMSO and 0.05 (v/v)% Tween-20. The compounds were diluted in steps covering a range from 500 M to 2, 100 nM. After the labelled protein and the compounds were mixed in equal volumes and incubated at 22 °C for 10 min to reach equilibrium, all of tests were repeated 3 times and run on a MonolithNT label-free instrument (NanoTemper GmbH, Munich, Germany) with 40% LED power and 20% MST power. The K_D_ values were calculated based on the Hill equation using the Monolith NT.015T analysis software.

## Additional Information

**How to cite this article**: Hu, L. *et al.* (±)-Japonones A and B, two pairs of new enantiomers with anti-KSHV activities from *Hypericum japonicum*. *Sci. Rep.* **6**, 27588; doi: 10.1038/srep27588 (2016).

## Supplementary Material

Supplementary Information

## Figures and Tables

**Figure 1:**
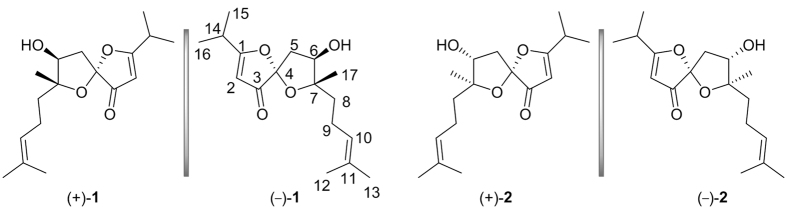
Structures of compounds 1 and 2.

**Table 1:** ^1^H (400 MHz) and ^13^C (100 MHz) NMR data for (±)-japonones A and B [(±)-1 and(±)- 2] (methanol-*d*_4_, *δ* in ppm, *J* in Hz).

NO.	(±)-Japonones A [(±)-1]		(±)-Japonones B [(±)-2]	
	*δ*_H_	*δ*_C_	*δ*_H_	*δ*_C_
1		200.9		200.4
2	5.40 s	99.3	5.43 s	99.7
3		203.3		202.5
4		112.1		111.0
5	2.52 dd (14.2, 7.0)	43.1	2.36 dd (13.4, 6.8)	42.6
	2.14 dd (14.2, 3.8)		2.22 dd (13.4, 8.6)	
6	4.20 dd (7.0, 3.8)	76.4	4.33 dd (8.6, 6.8)	76.1
7		92.6		91.0
8	1.55 m	41.3	1.65 m	41.6
9	2.07 m	23.8	2.10 m	23.6
10	5.12 ddd (7.2, 5.8, 1.4)	125.4	5.13 m	125.4
11		132.7		132.7
12	1.62 s	17.8	1.62 s	17.9
13	1.68 s	26.0	1.68 s	26.0
14	2.74 sept (7.0)	32.0	2.74 sept (7.0)	32.0
15	1.26 d (7.0)	19.7	1.24 d (7.0)	19.7
16	1.24 d (7.0)	19.7	1.24 d (7.0)	19.6
17	1.34 s	20.3	1.23 s	20.5

**Table 2:** Comparison of experimental and computed ^13^C NMR chemical shifts for (±)-japonone A [(±)-**1**] and QCP-**1** and (±)-japonone B [(±)-**2**] and QCP-**2** (*δ* in ppm).

NO.	(±)-Japonone A [(±)-1]		(±)-Japonone B [(±)-2]	
	Exptl.	Scal.calc. (QCP-1)	Exptl.	Scal.calc. (QCP-2)
1	200.9	201.2	200.4	198.2
2	99.3	95.6	99.7	98.3
3	203.3	202.4	202.5	199.0
4	112.1	114.4	111.0	116.4
5	43.1	41.2	42.6	43.2
6	76.4	79.8	76.1	80.0
7	92.6	96.0	91.0	96.0
8	41.3	40.2	41.6	40.6
9	23.8	24.9	23.6	23.7
10	125.4	124.8	125.4	124.5
11	132.7	131.5	132.7	134.3
12	17.8	16.7	17.9	15.5
13	26.0	25.9	26.0	24.9
14	32.0	33.4	32.0	32.2
15	19.7	20.9	19.7	19.9
16	19.7	17.9	19.6	16.9
17	20.3	19.7	20.5	18.6
	AveDev	1.5	AveDev	2.0
	MaxDev	3.7	MaxDev	5.3
	*R*^2^	0.9991	*R*^2^	0.9982

**Table 3:** Anti-KSHV activities of (±)-japonones A and B [(±)-1 and (±)-2] (*μ*M).

Compound	CC_50_	EC_50_	Selectivity index (CC_50_/EC_50_)
(+)-**1**	>500	166.0	>3.01
(−)-**1**	>500	189.8	>2.63
(+)-**2**	>500	398.0	>1.26
(−)-**2**	>500	251.2	>1.99
