# Supplementary material for: (±)-Japonones A and B, two pairs of new enantiomers with anti-KSHV activities from Hypericum japonicum
Source: Sci Rep. 2016 Jun 8;6:27588. doi: 10.1038/srep27588 (PMC4897785; doi:10.1038/srep27588)
Supplement: Supplementary Information [file srep27588-s1.doc]

(±)-Japonones A and B, two pairs of new enantiomers with anti-KSHV activities from Hypericum japonicum

Linzhen Hu^1,2^, Hucheng Zhu^1^, Lei Li^1^, Jinfeng Huang^1^, Weiguang Sun^1^, Junjun Liu^1^, Hua Li^1^, Jianping Wang, Zengwei Luo^1^, Yongbo Xue^1,*^, Yu Zhang^2,*^ & Yonghui Zhang^1,*^

^1^Hubei Key Laboratory of Natural Medicinal Chemistry and Resource Evaluation, School of Pharmacy, Tongji Medical College, Huazhong University of Science and Technology, Wuhan 430030, China

^2^Union Hospital, Tongji Medical College, Huazhong University of Science and Technology, Wuhan 430030, China

Content

[**Table** **S1**. Predicted binding free energies of compounds and targets towards KSHV 1](#__RefHeading___Toc446493917)

[**Figure S1**. Key 2D NMR correlations for **2**. 1](#__RefHeading___Toc446493918)

[**Figure** **S2**. The effects of (**±)-1** and (±)-**2** on Human iSLK.219 cells viabilities and the inhibitory effects of (**±)-1** and (±)-**2** on lytic replication of KSHV infecting Vero cells. 1](#__RefHeading___Toc446493919)

[**Figure** **S3**. Measurement of the affinity between compounds (±)-**1** and (±)-**2** with ERK using MST.. 2](#__RefHeading___Toc446493920)

[ECD spectra calculation 2](#__RefHeading___Toc446493921)

[^13^C NMR calculation section 30](#__RefHeading___Toc446493922)

[HRESIMS, NMR, UV, and IR spectra of (±)-**1** and (±)-**2** 34](#__RefHeading___Toc446493923)

[HRESIMS and ^1^H NMR spectra of compounds (−)-**1a**, (−)-**1b**, (−)-**2a**, and (−)-**2b** 44](#__RefHeading___Toc446493926)

**Table S1. Predicted binding free energies of compounds and targets towards KSHV (Total Score).**

| **PDB/ID** | **Protein name** | (+)-**1** | (–)-**1** | (+)-**2** | (–)-**2** |
| --- | --- | --- | --- | --- | --- |
| 2PBK | KSHV protease | 5.93 | 5.11 | 5.92 | 6.71 |
| 4K2J | KSHV LANA | 5.57 | 6.54 | 5.52 | 5.32 |
| 3IW4 | PKC | 6.20 | 4.53 | 5.46 | 6.48 |
| 3PY3 | P38 | 3.3 | 3.27 | 6.20 | 4.97 |
| 3V6R | JNK | 7.66 | 5.86 | 5.96 | 7.29 |
| 4QYY | ERK | 7.38 | 7.63 | 5.52 | 7.23 |


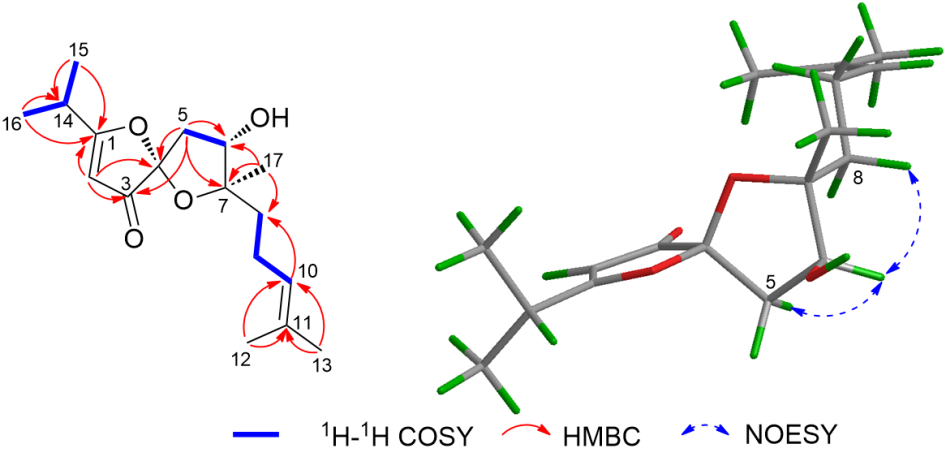


**Figure S1**. Key 2D NMR correlations for **2**.


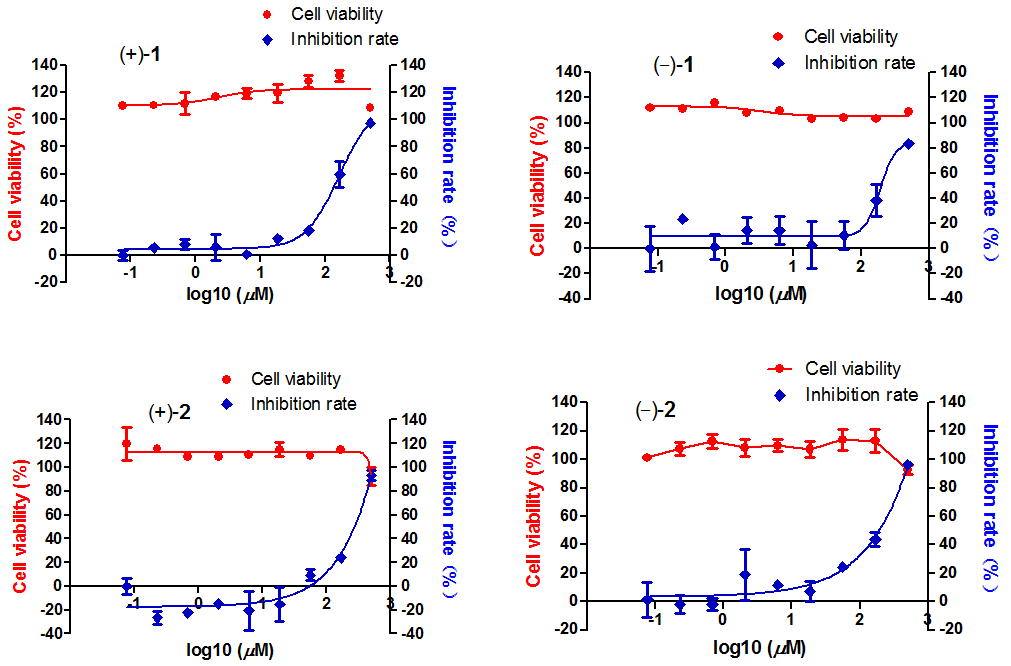


**Figure** **S2**. The effects of (**±)-1** and (±)-**2** on Human iSLK.219 cells viabilities were measured *in vitro*, and the inhibitory effects of (**±)-1** and (±)-**2** on lytic replication of KSHV infecting Vero cells were measured.


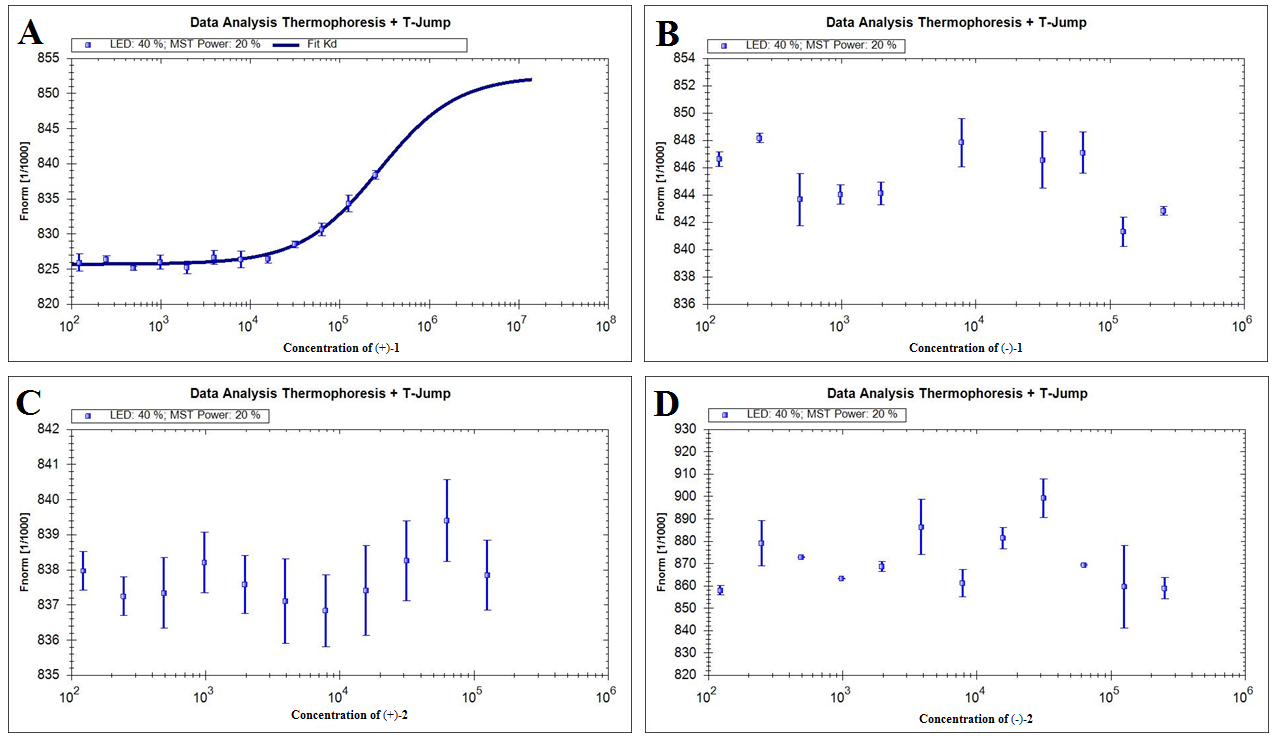


**Figure** **S3**. Measurement of the affinity between compounds (±)-**1** and (±)-**2** with ERK using MST. The resulting binding curve is shown. Based on the resulting binding curve, a K_D_ of 274 (± 9.5) × 10^–6^ M was calculated for (+)-**1**, and the other compounds exhibited no binding.

**ECD spectra calculation**

Conformational analyses were performed through both BALLOON and confab programs^1,2^ in order to establish the absolute structures of compounds (±)-**1** and (±)-**2**.The BALLOON program explores conformational spaces with genetic algorithm, and synchronously, the confab program systematically generates diverse low energy conformations that are proposed to be close to crystal structures. The conformations generated by the above programs were assembled together by the removal of duplicated conformations whose root mean square (RMS) distance was less than 0.5 Å. Semi-empirical PM3 quantum mechanical geometry optimizations were executed on conformations through the Gaussian 09 program^3^. Duplicated conformations after geometry optimization were subsequently identified and disposed. Remaining conformations were further optimized at B3LYP/6-31G* level of theory in methanol solvent with IEFPCM3 solvation model using Gaussian 09 program^4^, and duplicated conformations presenting after these calculations were removed according to the same RMS criteria above. Harmonic vibrational frequencies were fulfilled to build the stability of the finally obtained conformers. Oscillator strengths and rotational strengths of 20 weakest electronic excitations of each conformer were calculated by the TDDFT methodology at the B3LYP/6-311++G** level of theory adopting methanol as solvent by the IEFPCM solvation model carried out in Gaussian 09 program. The ECD spectra data for each conformer were then simulated by using a Gaussian function with a band width *σ* of 0.45 eV. Calculated spectra for each conformation were combined after Boltzmann weighting according to their population contribution.

1. M. J. Vainio, M. S. Johnson, 2007. Generating conformer ensembles using a multiobjective genetic algorithm. *J. Chem. Inf. Model*, **47**, 2462–2474.
2. N. M. O′Boyle, T. Vandermeersch, C. J. Flynn, A. R. Maguire, G. R. Hutchison, 2011. Confab-systematic generation of diverse low-energy conformers. *J. Cheminformatics*,**3**, 1–9.
3. Gaussian 09, Revision C.01, M. J. Frisch, G. W. Trucks, H. B. Schlegel, G. E. Scuseria, M. A. Robb, J. R. Cheeseman, G. Scalmani, V. Barone, B. Mennucci, G. A. Petersson, H. Nakatsuji, M. Caricato, X. Li, H. P. Hratchian, A. F. Izmaylov, J. Bloino, G. Zheng, J. L. Sonnenberg, M. Hada, M. Ehara, K. Toyota, R. Fukuda, J. Hasegawa, M. Ishida, T. Nakajima, Y. Honda, O. Kitao, H. Nakai, T. Vreven, J. A. Montgomery, J. E. Peralta, F. Ogliaro, M. Bearpark, J. J. Heyd, E. Brothers, K. N. Kudin, V. N. Staroverov, T. Keith, R. Kobayashi, J. Normand, K. Raghavachari, A. Rendell, J. C. Burant, S. S. Iyengar, J. Tomasi, M. Cossi,N.Rega, J. M. Millam, M. Klene, J. E. Knox, J. B. Cross, V. Bakken, C. Adamo, J. Jaramillo, R. Gomperts, R. E. Stratmann, O. Yazyev, A. J. Austin, R. Cammi, C. Pomelli, J. W. Ochterski, R. L. Martin, K. Morokuma, V. G. Zakrzewski, G. A. Voth, P. Salvador, J. J. Dannenberg, , S. Dapprich, A. D. Daniels, O. Farkas, J. B. Foresman, J. V. Ortiz, J. Cioslowski, D. J. Fox, *Gaussian, Inc., Wallingford CT*, 2010.
4. J. Tomasi, B. Mennucci, R. Cammi, 2005. Quantum mechanical continuum solvation models. *Chem. Rev.*,**105**, 2999–3094.

Optimized geometries of predominant conformers for compound 4*R*,6*R*,7*S*-**1** at the B3LYP/6-31G (d,p) level in methanol solution.


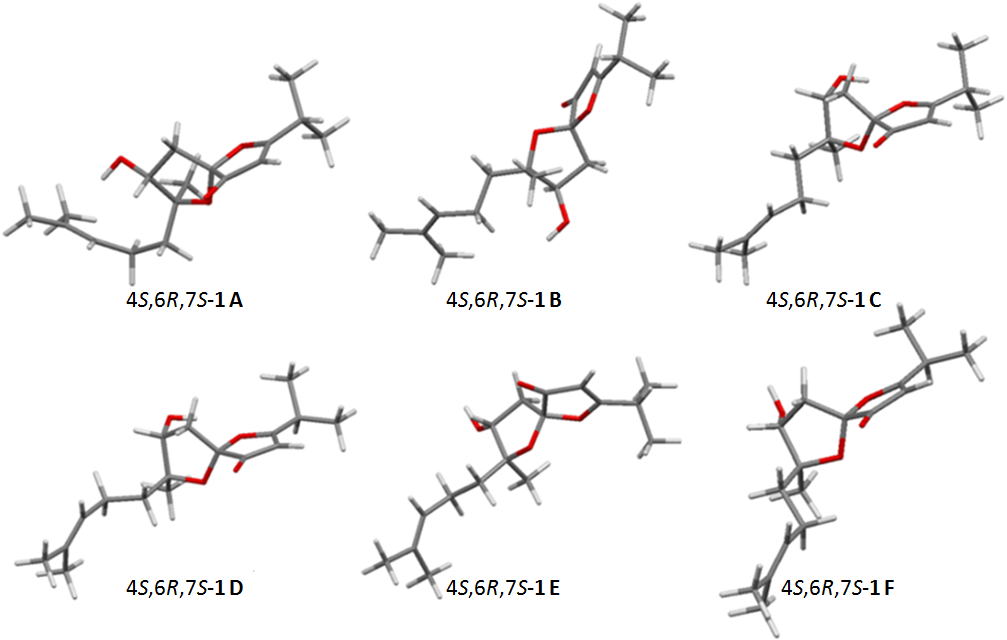


Important thermodynamic parameters (a.u.) and Boltzmann distributions of the optimized compound 4*S,*6*R*,7*S*-**1** at B3LYP/6-31G (d,p) level in methanol solution.

| Conformations | E+ZPE | G | % |
| --- | --- | --- | --- |
| 4*S,*6*R*,7*S*-**1 A** | -963.943343 | -963.996907 | 1.94 |
| 4*S,*6*R*,7*S*-**1 B** | -963.941126 | -963.996558 | 1.34 |
| 4*S*,6*R*,7*S*-**1 C** | -963.945613 | -964.000482 | 85.33 |
| 4*S*,6*R*,7*S*-**1 D** | -963.944778 | -963.998240 | 7.95 |
| 4*S*,6*R*,7*S*-**1 E** | -963.941805 | -963.996440 | 1.18 |
| 4*S*,6*R*,7*S*-**1 F** | -963.943264 | -963.997055 | 2.27 |

E+ZPE, G: total energy with zero point energy (ZPE) and Gibbs free energy in methanol solution at B3LYP/6-31G (d,p) level. %: Boltzmann distributions, using the relative Gibbs free energies as weighting factors.

Optimized Z-matrixes of compound 4*S*,6*R*,7*S*-**1** in methanol solution (Å) at B3LYP/6-31G (d,p) level.

| 4*S,*6*R*,7*S*-**1 A** | | | | 4*S*,6*R*,7*S*-**1 B** | | | |
| --- | --- | --- | --- | --- | --- | --- | --- |
| C | -3.517043 | 1.545547 | -0.211513 | C | 3.304224 | -1.554722 | 0.517035 |
| C | -3.603707 | 0.205817 | 0.022908 | C | 3.583255 | -0.447953 | -0.227999 |
| C | -2.123319 | 1.904782 | -0.192025 | C | 1.944522 | -1.441291 | 0.977698 |
| C | 3.986325 | 0.394736 | -0.586923 | C | -4.493587 | -0.167822 | -1.040211 |
| C | 4.889194 | -0.165926 | 0.241817 | C | -5.554114 | -0.569595 | -0.321326 |
| C | -0.450914 | 0.56502 | 1.310209 | C | 0.904681 | 0.918911 | 1.412415 |
| C | 0.965313 | 0.48461 | 0.72869 | C | -0.600426 | 1.007144 | 1.133061 |
| C | -1.347845 | 0.583768 | 0.066975 | C | 1.40542 | -0.108741 | 0.390759 |
| C | 0.751802 | -0.238302 | -0.633163 | C | -0.70921 | 0.612064 | -0.368826 |
| C | 5.671047 | 0.683347 | 1.218506 | C | -6.649274 | -1.392409 | -0.959956 |
| C | 5.242653 | -1.633875 | 0.267825 | C | -5.779724 | -0.263297 | 1.139839 |
| C | 0.670898 | -1.758951 | -0.488371 | C | -0.432414 | 1.785432 | -1.310739 |
| C | -4.785909 | -1.779131 | -0.94159 | C | 5.406289 | 1.256433 | -0.257337 |
| C | -4.934977 | -1.261397 | 1.549494 | C | 5.891267 | -1.141125 | -0.973589 |
| C | 3.170514 | -0.260909 | -1.674675 | C | -3.305633 | 0.645706 | -0.594425 |
| C | 1.700969 | 0.204293 | -1.759058 | C | -1.991842 | -0.153005 | -0.724436 |
| C | -4.814978 | -0.670003 | 0.129529 | C | 4.84434 | -0.022218 | -0.918742 |
| O | -1.55782 | 2.983832 | -0.330083 | O | 1.263559 | -2.172039 | 1.688206 |
| O | -2.413303 | -0.416091 | 0.197809 | O | 2.554061 | 0.427353 | -0.343934 |
| O | -0.553415 | 0.284892 | -1.041093 | O | 0.360472 | -0.377841 | -0.495135 |
| O | 1.819872 | -0.173326 | 1.643525 | O | -1.059128 | 2.310563 | 1.456753 |
| H | -4.350267 | 2.213768 | -0.378339 | H | 3.979804 | -2.371302 | 0.725625 |
| H | 3.856976 | 1.477321 | -0.518977 | H | -4.458039 | -0.466862 | -2.089876 |
| H | -0.658378 | -0.325649 | 1.909584 | H | 1.371949 | 1.889939 | 1.227383 |
| H | -0.598424 | 1.446711 | 1.937692 | H | 1.123769 | 0.619622 | 2.439603 |
| H | 1.327952 | 1.501053 | 0.517874 | H | -1.130833 | 0.251161 | 1.728045 |
| H | 6.747884 | 0.621064 | 1.00937 | H | -6.770319 | -2.355665 | -0.444684 |
| H | 5.375792 | 1.736274 | 1.175098 | H | -7.619489 | -0.880673 | -0.889379 |
| H | 5.534473 | 0.328736 | 2.248985 | H | -6.446992 | -1.594257 | -2.016667 |
| H | 4.629352 | -2.241059 | -0.401415 | H | -5.888297 | -1.1933 | 1.714699 |
| H | 6.293511 | -1.774535 | -0.020152 | H | -4.974223 | 0.318354 | 1.594089 |
| H | 5.143267 | -2.037772 | 1.283843 | H | -6.716378 | 0.294765 | 1.276721 |
| H | 1.613367 | -2.169471 | -0.116457 | H | 0.543988 | 2.235622 | -1.10818 |
| H | 0.448417 | -2.214925 | -1.458874 | H | -1.188871 | 2.566764 | -1.199776 |
| H | -0.117314 | -2.047625 | 0.213155 | H | -0.438316 | 1.436366 | -2.348685 |
| H | -3.925377 | -2.439987 | -0.79691 | H | 4.66107 | 2.056612 | -0.241832 |
| H | -4.729602 | -1.356765 | -1.950373 | H | 6.279112 | 1.606768 | -0.817434 |
| H | -5.697501 | -2.381723 | -0.873336 | H | 5.719048 | 1.053579 | 0.773173 |
| H | -4.080912 | -1.907357 | 1.776925 | H | 6.22687 | -1.42029 | 0.031626 |
| H | -5.848499 | -1.860349 | 1.623411 | H | 6.766653 | -0.800506 | -1.535461 |
| H | -4.981129 | -0.471774 | 2.30683 | H | 5.498631 | -2.03686 | -1.465784 |
| H | 3.62997 | -0.003491 | -2.641078 | H | -3.243203 | 1.550109 | -1.215531 |
| H | 3.216534 | -1.352229 | -1.609358 | H | -3.439405 | 0.986945 | 0.438152 |
| H | 1.279258 | -0.169254 | -2.700335 | H | -1.894969 | -0.519855 | -1.754633 |
| H | 1.666001 | 1.300152 | -1.817126 | H | -2.043663 | -1.039273 | -0.079983 |
| H | -5.682554 | -0.026318 | -0.055174 | H | 4.550479 | 0.240317 | -1.945254 |
| H | 2.724381 | -0.099802 | 1.282047 | H | -2.026728 | 2.286577 | 1.5176 |
| 4*S*,6*R*,7*S*-**1 C** | | | | 4*S*,6*R*,7*S*-**1 D** | | | |
| C | 2.772641 | 2.088073 | 0.14696 | C | 3.265607 | 1.701974 | 0.155096 |
| C | 3.280369 | 0.837383 | 0.335113 | C | 3.487318 | 0.366552 | 0.313964 |
| C | 1.421626 | 1.958131 | -0.334776 | C | 1.88199 | 1.890371 | -0.200376 |
| C | -4.098369 | 0.722818 | 0.049545 | C | -4.520651 | 0.188958 | -0.547889 |
| C | -5.362228 | 0.40066 | 0.369943 | C | -5.661753 | 0.325558 | 0.146722 |
| C | 0.850279 | -0.114537 | -1.830192 | C | 0.696989 | 0.015983 | -1.592522 |
| C | 0.18087 | -1.45435 | -1.484849 | C | -0.200902 | -1.153687 | -1.159412 |
| C | 1.154906 | 0.436191 | -0.441602 | C | 1.260143 | 0.47388 | -0.253126 |
| C | -0.631516 | -1.093816 | -0.203371 | C | -0.763171 | -0.65721 | 0.207731 |
| C | -6.52706 | 1.135598 | -0.252509 | C | -6.739344 | 1.275892 | -0.322523 |
| C | -5.759824 | -0.680414 | 1.346262 | C | -5.997879 | -0.416139 | 1.418207 |
| C | -0.673465 | -2.207643 | 0.836902 | C | -0.860867 | -1.745174 | 1.271822 |
| C | 4.52784 | -0.405715 | 2.11352 | C | 5.186133 | -1.246888 | -0.548945 |
| C | 5.386262 | -0.36373 | -0.286105 | C | 5.863225 | 0.536411 | 1.140838 |
| C | -2.814354 | 0.113307 | 0.549625 | C | -3.334702 | -0.691096 | -0.24646 |
| C | -2.034463 | -0.580876 | -0.585891 | C | -2.069919 | 0.142629 | 0.049719 |
| C | 4.636684 | 0.416918 | 0.813453 | C | 4.738917 | -0.386092 | 0.654206 |
| O | 0.581133 | 2.796586 | -0.639414 | O | 1.238539 | 2.903962 | -0.445876 |
| O | 2.420963 | -0.167155 | 0.036652 | O | 2.3932 | -0.408836 | 0.109881 |
| O | 0.119958 | 0.036747 | 0.384091 | O | 0.245761 | 0.323785 | 0.673853 |
| O | 1.137781 | -2.491807 | -1.304831 | O | 0.521132 | -2.378167 | -1.085791 |
| H | 3.29377 | 3.01483 | 0.342123 | H | 3.995015 | 2.487689 | 0.286168 |
| H | -3.958451 | 1.524417 | -0.679177 | H | -4.409053 | 0.796187 | -1.448686 |
| H | 1.73715 | -0.231734 | -2.455782 | H | 1.462495 | -0.281277 | -2.31187 |
| H | 0.154351 | 0.569887 | -2.324826 | H | 0.112086 | 0.839493 | -2.014209 |
| H | -0.486777 | -1.804834 | -2.275512 | H | -1.010249 | -1.336898 | -1.869212 |
| H | -7.186678 | 0.443189 | -0.794598 | H | -7.684078 | 0.743662 | -0.50258 |
| H | -7.149017 | 1.614971 | 0.516708 | H | -6.95454 | 2.036505 | 0.441196 |
| H | -6.196428 | 1.909347 | -0.952934 | H | -6.457375 | 1.790899 | -1.246418 |
| H | -6.411841 | -1.41827 | 0.858378 | H | -5.203806 | -1.086513 | 1.755064 |
| H | -4.90854 | -1.216156 | 1.772449 | H | -6.207855 | 0.292232 | 2.231504 |
| H | -6.34218 | -0.257062 | 2.176594 | H | -6.910767 | -1.013737 | 1.287239 |
| H | -1.237196 | -3.064008 | 0.45199 | H | 0.120786 | -2.179797 | 1.473335 |
| H | -1.15787 | -1.859889 | 1.753543 | H | -1.526948 | -2.550013 | 0.947708 |
| H | 0.333803 | -2.546965 | 1.086797 | H | -1.252577 | -1.323577 | 2.203588 |
| H | 3.97034 | -1.332534 | 1.94469 | H | 4.388733 | -1.919003 | -0.878717 |
| H | 4.022564 | 0.161312 | 2.902295 | H | 6.051932 | -1.853616 | -0.265043 |
| H | 5.530035 | -0.667479 | 2.468018 | H | 5.474949 | -0.611182 | -1.393431 |
| H | 4.862794 | -1.292851 | -0.534015 | H | 6.729482 | -0.062636 | 1.438487 |
| H | 6.390725 | -0.620162 | 0.065819 | H | 5.550031 | 1.133269 | 2.003582 |
| H | 5.484968 | 0.230984 | -1.200205 | H | 6.186205 | 1.220477 | 0.347922 |
| H | -2.175436 | 0.905088 | 0.962527 | H | -3.541959 | -1.35894 | 0.594356 |
| H | -3.004256 | -0.594184 | 1.361974 | H | -3.147545 | -1.338771 | -1.116123 |
| H | -1.937653 | 0.123711 | -1.422345 | H | -2.222803 | 0.707953 | 0.976975 |
| H | -2.616267 | -1.431459 | -0.963133 | H | -1.931685 | 0.88905 | -0.74361 |
| H | 5.19065 | 1.338228 | 1.026294 | H | 4.465059 | -1.072911 | 1.467806 |
| H | 1.857587 | -2.141337 | -0.751818 | H | 1.348599 | -2.20602 | -0.603883 |
| 4*S*,6*R*,7*S*-**1 E** | | | | 4*S*,6*R*,7*S*-**1 F** | | | |
| C | 3.47795 | -0.984042 | -1.410619 | C | -2.694877 | -1.855075 | -0.185203 |
| C | 3.633776 | 0.111231 | -0.614609 | C | -3.237039 | -0.696822 | 0.290067 |
| C | 2.191061 | -1.55889 | -1.11838 | C | -1.361795 | -1.560171 | -0.639022 |
| C | -4.470559 | -0.314289 | -0.375691 | C | 3.985564 | -0.918999 | -0.067638 |
| C | -5.530982 | 0.488464 | -0.561526 | C | 5.293385 | -0.796072 | 0.212046 |
| C | 1.17052 | -1.333653 | 1.279968 | C | -0.849185 | 0.788575 | -1.628898 |
| C | -0.367796 | -1.244338 | 1.291407 | C | -0.01968 | 1.955287 | -1.069844 |
| C | 1.554403 | -0.645461 | -0.035896 | C | -1.150864 | -0.043591 | -0.384499 |
| C | -0.658122 | 0.02111 | 0.449415 | C | 0.750803 | 1.290372 | 0.099046 |
| C | -6.658566 | 0.076101 | -1.479791 | C | 6.325117 | -1.583326 | -0.562791 |
| C | -5.72528 | 1.835065 | 0.093399 | C | 5.870359 | 0.097218 | 1.284099 |
| C | -0.529061 | 1.317029 | 1.253904 | C | 0.94152 | 2.190807 | 1.315749 |
| C | 4.359319 | 2.490303 | -0.885911 | C | -5.361352 | 0.561631 | -0.070673 |
| C | 5.40393 | 1.011356 | 0.908074 | C | -5.401618 | -1.690463 | 1.123281 |
| C | -3.255436 | -0.087781 | 0.486185 | C | 2.81365 | -0.226349 | 0.578077 |
| C | -1.962792 | -0.051798 | -0.355823 | C | 2.07706 | 0.69313 | -0.417072 |
| C | 4.789751 | 1.058671 | -0.506127 | C | -4.597853 | -0.412858 | 0.853961 |
| O | 1.622662 | -2.552312 | -1.558175 | O | -0.498542 | -2.269408 | -1.145557 |
| O | 2.585856 | 0.368919 | 0.204088 | O | -2.419305 | 0.379593 | 0.221585 |
| O | 0.407583 | -0.038314 | -0.551415 | O | -0.115132 | 0.174328 | 0.514063 |
| O | -0.931597 | -1.129877 | 2.588481 | O | -0.84423 | 3.000939 | -0.565939 |
| H | 4.197526 | -1.350704 | -2.129182 | H | -3.173295 | -2.823177 | -0.20556 |
| H | -4.461835 | -1.261491 | -0.919705 | H | 3.711389 | -1.599625 | -0.876797 |
| H | 1.582276 | -0.779528 | 2.127558 | H | -1.754374 | 1.112911 | -2.14734 |
| H | 1.535147 | -2.362712 | 1.32577 | H | -0.254226 | 0.178702 | -2.316303 |
| H | -0.787182 | -2.111765 | 0.763769 | H | 0.680579 | 2.349094 | -1.817456 |
| H | -7.613902 | 0.032768 | -0.937716 | H | 7.040087 | -0.913572 | -1.061567 |
| H | -6.796362 | 0.807327 | -2.288885 | H | 6.917891 | -2.223614 | 0.105765 |
| H | -6.480271 | -0.904065 | -1.933559 | H | 5.866001 | -2.220443 | -1.325495 |
| H | -5.849689 | 2.618426 | -0.667093 | H | 6.432382 | -0.494731 | 2.019958 |
| H | -6.64442 | 1.842615 | 0.695829 | H | 6.586996 | 0.807242 | 0.848379 |
| H | -4.896982 | 2.127037 | 0.743014 | H | 5.115101 | 0.673226 | 1.823796 |
| H | -0.655548 | 2.176567 | 0.587316 | H | 1.389934 | 1.62736 | 2.138688 |
| H | 0.45324 | 1.39581 | 1.72912 | H | -0.017297 | 2.591818 | 1.647446 |
| H | -1.288101 | 1.367135 | 2.038649 | H | 1.603046 | 3.028602 | 1.07106 |
| H | 3.598334 | 2.866252 | -0.194622 | H | -5.563772 | 0.095428 | -1.041632 |
| H | 3.950174 | 2.526549 | -1.901028 | H | -4.789632 | 1.478447 | -0.23936 |
| H | 5.224711 | 3.15954 | -0.841705 | H | -6.319278 | 0.8296 | 0.386425 |
| H | 4.67914 | 1.338864 | 1.660272 | H | -5.613775 | -2.229277 | 0.19282 |
| H | 6.272533 | 1.676246 | 0.955386 | H | -6.359347 | -1.43399 | 1.586982 |
| H | 5.733601 | -0.000853 | 1.164992 | H | -4.867903 | -2.367571 | 1.798166 |
| H | -3.347688 | 0.837346 | 1.062936 | H | 2.099273 | -0.981782 | 0.931803 |
| H | -3.175698 | -0.900389 | 1.221292 | H | 3.129434 | 0.344918 | 1.455765 |
| H | -1.997021 | 0.80853 | -1.036345 | H | 1.869401 | 0.119911 | -1.329941 |
| H | -1.912125 | -0.948413 | -0.987171 | H | 2.736667 | 1.519914 | -0.710231 |
| H | 5.542131 | 0.716287 | -1.225764 | H | -4.425285 | 0.106141 | 1.807886 |
| H | -0.870722 | -1.999171 | 3.015177 | H | -1.321473 | 3.381088 | -1.320593 |

Key transitions, oscillator strengths, and rotatory strengths in the ECD of conformers 4*S*,6*R*,7*S*-**1 C** at B3LYP /6-311++G (d,p) level.

| Species | Excited State | *∆E(eV)^a^* | *λ(nm)^b^* | *f*^c^ | *R_vel_^d^* |
| --- | --- | --- | --- | --- | --- |
| 4*S*,6*R*,7*S*-**1 C** | 78->81 | 3.9246 | 315.92 | 0.0019 | 14.4126 |
|  | 80->81 | 4.2253 | 293.43 | 0.0003 | -0.408 |
|  | 79->81 | 4.8146 | 257.52 | 0.2194 | -45.7659 |
|  | 77->81 | 5.3375 | 232.29 | 0.023 | -1.6993 |
|  | 76->81 | 5.4866 | 225.98 | 0.0427 | 20.8869 |
|  | 80->82 | 5.5041 | 225.26 | 0.0016 | -7.253 |
|  | 80->83 | 5.86 | 211.58 | 0.003 | 3.4289 |
|  | 80->85 | 6.0095 | 206.31 | 0.0053 | 4.2455 |
|  | 80->84 | 6.0651 | 204.42 | 0.0746 | 2.5643 |
|  | 79->82 | 6.1336 | 202.14 | 0.0093 | -2.6978 |
|  | 80->87 | 6.2189 | 199.37 | 0.0219 | 16.6842 |
|  | 78->82 | 6.3393 | 195.58 | 0.0041 | 10.7469 |
|  | 80->86 | 6.3559 | 195.07 | 0.2025 | -12.409 |
|  | 80->88 | 6.4226 | 193.04 | 0.0589 | 18.2421 |
|  | 79->83 | 6.5071 | 190.54 | 0.0066 | -1.4465 |
|  | 75->81 | 6.5455 | 189.42 | 0.0216 | 14.372 |
|  | 79->85 | 6.5833 | 188.33 | 0.005 | -7.7264 |
|  | 80->89 | 6.6275 | 187.08 | 0.1043 | -9.7464 |
|  | 79->85 | 6.6492 | 186.47 | 0.0381 | -1.7497 |
|  | 74->81 | 6.667 | 185.97 | 0.0292 | 6.4696 |
|  | 80->90 | 6.6811 | 185.57 | 0.0073 | 4.5823 |
|  | 78->83 | 6.6895 | 185.34 | 0.0016 | -5.789 |
|  | 73->81 | 6.7126 | 184.7 | 0.0052 | -8.5687 |
|  | 80->91 | 6.7487 | 183.71 | 0.0063 | -4.2457 |
|  | 78->84 | 6.756 | 183.52 | 0.0077 | 14.2917 |
|  | 80->92 | 6.7925 | 182.53 | 0.014 | 3.8462 |
|  | 78->85 | 6.8138 | 181.96 | 0.026 | 9.0368 |
|  | 79->87 | 6.825 | 181.66 | 0.0055 | -1.4364 |
|  | 71->81 | 6.8467 | 181.09 | 0.0013 | 5.1523 |
|  | 80->93 | 6.8548 | 180.87 | 0.0101 | -1.2955 |
|  | 79->86 | 6.8676 | 180.53 | 0.005 | -0.7329 |
|  | 77->82 | 6.8837 | 180.11 | 0.0109 | 5.0265 |
|  | 72->81 | 6.9123 | 179.37 | 0.0371 | -18.8454 |
|  | 80->92 | 6.9339 | 178.81 | 0.0123 | 13.2782 |
|  | 79->88 | 6.9854 | 177.49 | 0.0073 | 1.8723 |
|  | 78->86 | 6.9999 | 177.12 | 0.0026 | 0.3258 |
|  | 80->94 | 7.0038 | 177.02 | 0.0645 | -0.4502 |
|  | 78->86 | 7.025 | 176.49 | 0.0061 | 3.236 |
|  | 78->87 | 7.0433 | 176.03 | 0.003 | 5.9383 |
|  | 79->89 | 7.0682 | 175.41 | 0.0005 | -0.6762 |

*^a^*Excitation energy. *^b^*Wavelength. *^c^*Oscillator strength. *^d^*Rotatory strength in velocity form (10^-40^cgs.).

Optimized geometries of predominant conformers for compound 4*R*,6*R*,7*S*-**1** at the B3LYP/6-31G (d,p) level in methanol solution.


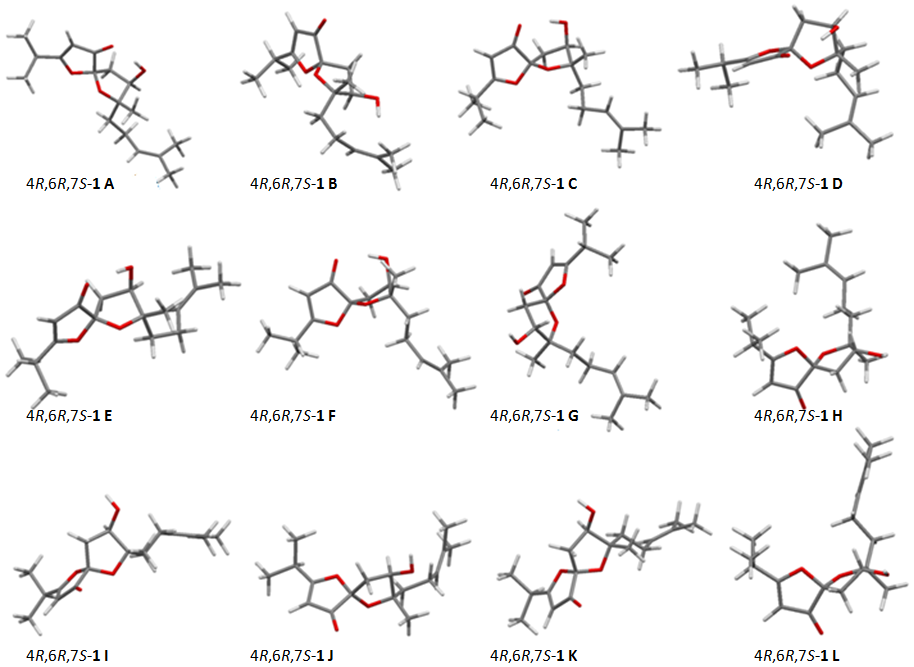


Important thermodynamic parameters (a.u.) and Boltzmann distributions of the optimized compound 4*R*,6*R*,7*S*-**1** at B3LYP/6-31G (d,p) level in methanol solution.

| Conformations | E+ZPE | G | % |
| --- | --- | --- | --- |
| 4*R*,6*R*,7*S*-**1 A** | -963.945043 | -963.998040 | 7.21 |
| 4*R*,6*R*,7*S*-**1 B** | -963.944692 | -963.997886 | 6.13 |
| 4*R*,6*R*,7*S*-**1 C** | -963.944320 | -963.997532 | 4.21 |
| 4*R*,6*R*,7*S*-**1 D** | -963.946034 | -963.999004 | 20.02 |
| 4*R*,6*R*,7*S*-**1 E** | -963.946282 | -963.998983 | 19.58 |
| 4*R*,6*R*,7*S*-**1 F** | -963.945720 | -963.998592 | 12.94 |
| 4*R*,6*R*,7*S*-**1 G** | -963.943081 | -963.997760 | 5.36 |
| 4*R*,6*R*,7*S*-**1 H** | -963.943162 | -963.997738 | 5.24 |
| 4*R*,6*R*,7*S*-**1 I** | -963.943143 | -963.997765 | 5.39 |
| 4*R*,6*R*,7*S*-**1 J** | -963.943209 | -963.998040 | 7.21 |
| 4*R*,6*R*,7*S*-**1 K** | -963.943104 | -963.997649 | 4.77 |
| 4*R*,6*R*,7*S*-**1 L** | -963.942833 | -963.996801 | 1.94 |

E+ZPE, G: total energy with zero point energy (ZPE) and Gibbs free energy in methanol solution at B3LYP/6-31G (d,p) level. %: Boltzmann distributions, using the relative Gibbs free energies as weighting factors.

Optimized Z-matrixes of compound 4*R*,6*R*,7*S*-**1** in methanol solution (Å) at B3LYP/6-31G (d,p) level.

| 4*R*,6*R*,7*S*-**1 A** | | | | 4*R*,6*R*,7*S*-**1 B** | | | |
| --- | --- | --- | --- | --- | --- | --- | --- |
| C | -3.572797 | 0.995883 | 0.43446 | C | -3.777965 | 1.077195 | -0.146101 |
| C | -3.500782 | -0.253998 | -0.113926 | C | -3.44612 | -0.241376 | -0.041144 |
| C | -2.250978 | 1.547332 | 0.409942 | C | -2.557659 | 1.831214 | -0.258142 |
| C | 3.962585 | -1.361728 | 0.114087 | C | 3.423645 | -1.181616 | 0.558915 |
| C | 5.132531 | -0.704909 | 0.050295 | C | 4.542817 | -0.970395 | -0.16173 |
| C | -0.54077 | 0.888762 | -1.429666 | C | -0.496577 | 0.738901 | -1.421722 |
| C | 0.827411 | 1.243872 | -0.826839 | C | 0.803449 | 0.191666 | -0.83154 |
| C | -1.335342 | 0.454811 | -0.191561 | C | -1.409388 | 0.787702 | -0.205769 |
| C | 0.973042 | 0.174398 | 0.292882 | C | 0.822904 | 0.806536 | 0.602493 |
| C | 6.181771 | -0.894987 | 1.121493 | C | 4.957759 | -1.939783 | -1.245666 |
| C | 5.531753 | 0.250986 | -1.048183 | C | 5.49705 | 0.182094 | 0.043908 |
| C | 1.782374 | 0.638976 | 1.496204 | C | 1.468999 | 2.191403 | 0.638437 |
| C | -4.282168 | -2.543809 | 0.491534 | C | -4.164189 | -2.381618 | -1.122573 |
| C | -5.976903 | -0.733838 | -0.104623 | C | -4.062687 | -2.179806 | 1.415676 |
| C | 2.815844 | -1.34585 | -0.864239 | C | 2.885162 | -0.381837 | 1.720306 |
| C | 1.407917 | -1.211345 | -0.235608 | C | 1.36076 | -0.1345 | 1.693734 |
| C | -4.566009 | -1.280526 | -0.352872 | C | -4.334084 | -1.44183 | 0.088889 |
| O | -1.811692 | 2.645959 | 0.760506 | O | -2.349537 | 3.033486 | -0.381234 |
| O | -2.257572 | -0.628059 | -0.502911 | O | -2.117297 | -0.494546 | -0.065095 |
| O | -0.416439 | 0.031643 | 0.772961 | O | -0.604661 | 0.986406 | 0.911809 |
| O | 0.85702 | 2.577618 | -0.342228 | O | 1.897845 | 0.542895 | -1.655614 |
| H | -4.463901 | 1.480151 | 0.805507 | H | -4.781603 | 1.478689 | -0.135503 |
| H | 3.812459 | -2.030172 | 0.964882 | H | 2.844933 | -2.07781 | 0.323748 |
| H | -0.989684 | 1.736883 | -1.951378 | H | -0.336789 | 1.758375 | -1.789471 |
| H | -0.474954 | 0.05004 | -2.127465 | H | -0.907091 | 0.130641 | -2.229824 |
| H | 1.632352 | 1.170066 | -1.561475 | H | 0.725979 | -0.901237 | -0.740538 |
| H | 5.85336 | -1.59482 | 1.896569 | H | 5.067573 | -1.429992 | -2.212348 |
| H | 7.11977 | -1.275544 | 0.692996 | H | 5.937047 | -2.38155 | -1.015385 |
| H | 6.429088 | 0.060413 | 1.605368 | H | 4.236858 | -2.75427 | -1.367042 |
| H | 6.417815 | -0.122236 | -1.580659 | H | 5.664972 | 0.71854 | -0.89911 |
| H | 4.745217 | 0.418695 | -1.787658 | H | 5.154125 | 0.903063 | 0.789127 |
| H | 5.813274 | 1.226025 | -0.627284 | H | 6.478182 | -0.192439 | 0.366604 |
| H | 1.374982 | 1.571431 | 1.891731 | H | 1.415869 | 2.600817 | 1.652946 |
| H | 2.826858 | 0.800365 | 1.218294 | H | 0.945305 | 2.877269 | -0.034145 |
| H | 1.754602 | -0.122277 | 2.282902 | H | 2.51707 | 2.150511 | 0.331853 |
| H | -3.28188 | -2.938193 | 0.292963 | H | -4.381788 | -1.863179 | -2.062304 |
| H | -5.015617 | -3.319055 | 0.248542 | H | -4.852872 | -3.227756 | -1.030836 |
| H | -4.361183 | -2.320033 | 1.561245 | H | -3.143341 | -2.773664 | -1.173701 |
| H | -6.182761 | 0.147295 | -0.720966 | H | -4.216951 | -1.520767 | 2.276496 |
| H | -6.116347 | -0.45917 | 0.947079 | H | -3.035327 | -2.556167 | 1.45033 |
| H | -6.717756 | -1.500851 | -0.350566 | H | -4.744383 | -3.031396 | 1.509752 |
| H | 2.959802 | -0.583502 | -1.636797 | H | 3.090247 | -0.945387 | 2.643168 |
| H | 2.814409 | -2.307237 | -1.400748 | H | 3.415025 | 0.568961 | 1.831464 |
| H | 1.316097 | -1.917462 | 0.599983 | H | 1.068973 | 0.291906 | 2.66132 |
| H | 0.674434 | -1.536282 | -0.982242 | H | 0.834477 | -1.094314 | 1.606045 |
| H | -4.475119 | -1.564919 | -1.411163 | H | -5.364511 | -1.068629 | 0.099335 |
| H | 0.02726 | 2.72894 | 0.163444 | H | 2.673979 | 0.070143 | -1.29872 |
| 4*R*,6*R*,7*S*-**1 C** | | | | 4*R*,6*R*,7*S*-**1 D** | | | |
| C | -3.540794 | -0.212047 | -0.95849 | C | 2.945459 | 0.115593 | -1.052451 |
| C | -3.075383 | 0.911104 | -0.334396 | C | 2.718248 | -0.660962 | 0.048932 |
| C | -2.60295 | -1.262126 | -0.698587 | C | 1.952867 | 1.148449 | -1.056118 |
| C | 3.736365 | 0.455656 | -0.47175 | C | -3.48232 | -0.973729 | 0.619111 |
| C | 4.869962 | 1.108748 | -0.164681 | C | -3.603481 | -1.881439 | -0.363974 |
| C | -1.19465 | -1.312075 | 1.482204 | C | 0.928392 | 2.058665 | 1.150505 |
| C | -0.003241 | -2.225012 | 1.148122 | C | -0.388227 | 2.717695 | 0.706219 |
| C | -1.464514 | -0.62769 | 0.136959 | C | 1.047867 | 0.885482 | 0.171356 |
| C | 0.794906 | -1.353255 | 0.133412 | C | -1.246541 | 1.481934 | 0.304046 |
| C | 6.20011 | 0.636325 | -0.705784 | C | -4.92997 | -2.097472 | -1.055589 |
| C | 4.960858 | 2.335254 | 0.711542 | C | -2.486039 | -2.762204 | -0.869464 |
| C | 1.559311 | -2.166706 | -0.902887 | C | -2.244022 | 1.775867 | -0.8106 |
| C | -4.032948 | 2.632573 | 1.205183 | C | 2.515748 | -3.101068 | 0.52906 |
| C | -2.738231 | 3.32946 | -0.877956 | C | 4.759586 | -2.123589 | -0.185385 |
| C | 2.32993 | 0.770988 | -0.028845 | C | -2.274002 | -0.649311 | 1.458928 |
| C | 1.67437 | -0.317982 | 0.866623 | C | -1.9059 | 0.848544 | 1.552374 |
| C | -3.679797 | 2.27859 | -0.254644 | C | 3.445467 | -1.866682 | 0.562077 |
| O | -2.59309 | -2.456047 | -1.010814 | O | 1.760813 | 2.099779 | -1.818171 |
| O | -1.899162 | 0.750512 | 0.317041 | O | 1.65763 | -0.276257 | 0.800543 |
| O | -0.275056 | -0.65463 | -0.597972 | O | -0.243436 | 0.562785 | -0.257615 |
| O | -0.43165 | -3.469785 | 0.620319 | O | -0.182711 | 3.64359 | -0.34776 |
| H | -4.455497 | -0.285281 | -1.529485 | H | 3.734699 | -0.018867 | -1.776996 |
| H | 3.835084 | -0.41886 | -1.115624 | H | -4.376978 | -0.405323 | 0.884413 |
| H | -2.051036 | -1.879715 | 1.852692 | H | 1.770864 | 2.751656 | 1.096408 |
| H | -0.935951 | -0.554169 | 2.225824 | H | 0.868332 | 1.666813 | 2.169082 |
| H | 0.602474 | -2.455159 | 2.028839 | H | -0.866092 | 3.27517 | 1.516726 |
| H | 6.685336 | 1.421402 | -1.302971 | H | -4.847687 | -1.91179 | -2.135838 |
| H | 6.896063 | 0.396041 | 0.110467 | H | -5.267269 | -3.1381 | -0.94666 |
| H | 6.094084 | -0.253005 | -1.335363 | H | -5.711891 | -1.443751 | -0.655743 |
| H | 5.62795 | 2.148907 | 1.564765 | H | -2.386844 | -2.668191 | -1.959559 |
| H | 5.40056 | 3.175082 | 0.15561 | H | -1.516513 | -2.529834 | -0.423463 |
| H | 3.99682 | 2.663595 | 1.106544 | H | -2.705547 | -3.820691 | -0.670149 |
| H | 1.952037 | -1.516689 | -1.689473 | H | -2.767341 | 0.863954 | -1.106791 |
| H | 0.905498 | -2.91117 | -1.360525 | H | -1.73353 | 2.197117 | -1.678972 |
| H | 2.399917 | -2.686933 | -0.431916 | H | -2.986875 | 2.50186 | -0.463044 |
| H | -3.133211 | 2.668759 | 1.827573 | H | 1.591382 | -2.918502 | 1.083961 |
| H | -4.722584 | 1.899871 | 1.636999 | H | 3.027754 | -3.956734 | 0.980441 |
| H | -4.513397 | 3.615678 | 1.237379 | H | 2.256133 | -3.362345 | -0.502861 |
| H | -2.508557 | 3.089704 | -1.921361 | H | 4.576815 | -2.362176 | -1.239211 |
| H | -1.796885 | 3.389768 | -0.322716 | H | 5.27826 | -2.975582 | 0.264854 |
| H | -3.217992 | 4.313065 | -0.850122 | H | 5.426765 | -1.256751 | -0.139178 |
| H | 2.313181 | 1.71733 | 0.518917 | H | -2.479966 | -0.979963 | 2.488655 |
| H | 1.688057 | 0.925125 | -0.907148 | H | -1.392505 | -1.206551 | 1.131813 |
| H | 1.056691 | 0.184503 | 1.619242 | H | -1.228783 | 0.963145 | 2.407478 |
| H | 2.450821 | -0.858971 | 1.421877 | H | -2.799952 | 1.438077 | 1.795466 |
| H | -4.605605 | 2.244858 | -0.83989 | H | 3.671632 | -1.652896 | 1.616718 |
| H | -1.132025 | -3.284971 | -0.044872 | H | 0.407977 | 3.216438 | -1.007686 |
| 4*R*,6*R*,7*S*-**1 E** | | | | 4*R*,6*R*,7*S*-**1 F** | | | |
| C | -3.48991 | 1.319321 | -0.194986 | C | 3.511653 | 0.20366 | -0.182623 |
| C | -3.473344 | -0.045972 | -0.137075 | C | 2.849415 | -0.991053 | -0.180451 |
| C | -2.128073 | 1.760801 | -0.206472 | C | 2.536152 | 1.230256 | 0.03437 |
| C | 3.49616 | -1.438818 | -0.178895 | C | -3.524425 | -1.132245 | -0.643972 |
| C | 4.661647 | -0.846761 | -0.490155 | C | -4.649118 | -1.465255 | 0.010063 |
| C | -0.246443 | 0.302763 | -1.247386 | C | 0.362667 | 0.794931 | 1.38675 |
| C | 1.047485 | 0.841809 | -0.618597 | C | -0.524251 | 1.978542 | 0.965863 |
| C | -1.257771 | 0.484127 | -0.108784 | C | 1.164305 | 0.513729 | 0.115848 |
| C | 0.909986 | 0.370994 | 0.85628 | C | -0.78564 | 1.700573 | -0.550894 |
| C | 5.23305 | -0.955898 | -1.885108 | C | -5.771175 | -2.184021 | -0.703316 |
| C | 5.512369 | -0.04894 | 0.468568 | C | -4.921294 | -1.183025 | 1.468255 |
| C | 1.580685 | 1.299039 | 1.862207 | C | -0.747133 | 2.96826 | -1.404785 |
| C | -4.573852 | -1.911547 | -1.386601 | C | 4.830455 | -2.448352 | -0.733022 |
| C | -4.61261 | -1.854329 | 1.162013 | C | 3.113363 | -3.166095 | 1.010771 |
| C | 2.779426 | -1.480817 | 1.146654 | C | -2.305167 | -0.417718 | -0.119907 |
| C | 1.278855 | -1.111172 | 1.088789 | C | -2.091073 | 0.930147 | -0.837871 |
| C | -4.615539 | -1.014418 | -0.131808 | C | 3.358784 | -2.394278 | -0.306067 |
| O | -1.62725 | 2.886535 | -0.280749 | O | 2.652291 | 2.453561 | 0.154594 |
| O | -2.237556 | -0.595641 | -0.090449 | O | 1.507748 | -0.889692 | -0.00824 |
| O | -0.543563 | 0.490572 | 1.091543 | O | 0.354175 | 0.869873 | -0.969728 |
| O | 1.147267 | 2.252182 | -0.751597 | O | 0.10555 | 3.224076 | 1.221767 |
| H | -4.373868 | 1.940014 | -0.234456 | H | 4.57475 | 0.345466 | -0.309444 |
| H | 2.994435 | -1.996105 | -0.973627 | H | -3.466341 | -1.395539 | -1.702395 |
| H | -0.51901 | 0.846882 | -2.154433 | H | 0.986814 | 1.042008 | 2.2485 |
| H | -0.170311 | -0.759967 | -1.491605 | H | -0.22795 | -0.091999 | 1.626702 |
| H | 1.94286 | 0.423479 | -1.081712 | H | -1.466169 | 2.001118 | 1.520042 |
| H | 4.58884 | -1.543087 | -2.547324 | H | -5.988895 | -3.149419 | -0.224882 |
| H | 5.370374 | 0.037933 | -2.333701 | H | -6.702779 | -1.602168 | -0.660949 |
| H | 6.22604 | -1.426953 | -1.868583 | H | -5.534156 | -2.371408 | -1.755517 |
| H | 6.503317 | -0.511111 | 0.578816 | H | -4.1105 | -0.647819 | 1.967814 |
| H | 5.686378 | 0.963504 | 0.079123 | H | -5.837381 | -0.58659 | 1.579768 |
| H | 5.075826 | 0.043524 | 1.465764 | H | -5.094893 | -2.119156 | 2.017076 |
| H | 1.437375 | 0.926013 | 2.882051 | H | 0.208361 | 3.484299 | -1.295289 |
| H | 1.159419 | 2.303276 | 1.790779 | H | -1.546638 | 3.654718 | -1.108719 |
| H | 2.654667 | 1.368069 | 1.6663 | H | -0.88547 | 2.709827 | -2.459786 |
| H | -4.606282 | -1.314988 | -2.304217 | H | 5.135245 | -3.490158 | -0.872593 |
| H | -5.437484 | -2.584238 | -1.384579 | H | 5.481601 | -2.008833 | 0.030976 |
| H | -3.663968 | -2.519849 | -1.402212 | H | 4.997172 | -1.916597 | -1.675375 |
| H | -5.476962 | -2.526134 | 1.164419 | H | 2.057913 | -3.141543 | 1.295621 |
| H | -4.671246 | -1.217383 | 2.050704 | H | 3.704028 | -2.735874 | 1.827271 |
| H | -3.704548 | -2.461421 | 1.232157 | H | 3.413611 | -4.210907 | 0.884052 |
| H | 2.822613 | -2.513101 | 1.526508 | H | -2.380791 | -0.267022 | 0.961373 |
| H | 3.286709 | -0.869007 | 1.898405 | H | -1.421981 | -1.048255 | -0.294082 |
| H | 0.822054 | -1.387599 | 2.046735 | H | -2.930858 | 1.598641 | -0.607984 |
| H | 0.788158 | -1.733074 | 0.329425 | H | -2.126108 | 0.754413 | -1.921083 |
| H | -5.532484 | -0.415103 | -0.160093 | H | 2.743556 | -2.867609 | -1.084736 |
| H | 0.273191 | 2.636161 | -0.516471 | H | 1.014646 | 3.169521 | 0.852421 |
| 4*R*,6*R*,7*S*-**1 G** | | | | 4*R*,6*R*,7*S*-**1 H** | | | |
| C | -3.493591 | 0.155183 | -1.046379 | C | 3.103892 | 0.799163 | 1.348634 |
| C | -3.040161 | 0.870384 | 0.022539 | C | 2.547895 | 1.387093 | 0.251095 |
| C | -2.61827 | -0.974123 | -1.225258 | C | 2.548287 | -0.522577 | 1.471298 |
| C | 3.500651 | 1.349393 | -0.171062 | C | -3.969452 | -0.022323 | -0.058265 |
| C | 4.840337 | 1.438155 | -0.203445 | C | -4.411355 | 1.223511 | 0.178571 |
| C | -1.479489 | -2.031098 | 0.868756 | C | 1.873051 | -1.802327 | -0.697961 |
| C | -0.034551 | -1.927346 | 1.378798 | C | 0.490424 | -2.074928 | -1.308999 |
| C | -1.550653 | -0.864192 | -0.105426 | C | 1.564856 | -0.679156 | 0.281207 |
| C | 0.743233 | -1.416569 | 0.135813 | C | -0.478254 | -1.812901 | -0.123723 |
| C | 5.544671 | 2.666874 | 0.324592 | C | -5.656871 | 1.741834 | -0.503138 |
| C | 5.766789 | 0.376741 | -0.746729 | C | -3.756654 | 2.212416 | 1.112799 |
| C | 1.262891 | -2.557228 | -0.740047 | C | -0.734062 | -3.070123 | 0.708793 |
| C | -2.511639 | 3.263261 | 0.492922 | C | 3.385086 | 2.643581 | -1.744653 |
| C | -4.925671 | 2.546926 | 0.09926 | C | 1.490159 | 3.581845 | -0.322698 |
| C | 2.618987 | 0.219715 | -0.636592 | C | -2.760048 | -0.719439 | 0.509123 |
| C | 1.83319 | -0.40961 | 0.53167 | C | -1.763753 | -1.119424 | -0.597106 |
| C | -3.554558 | 2.133462 | 0.647044 | C | 2.78833 | 2.749272 | -0.326032 |
| O | -2.62782 | -1.888662 | -2.041743 | O | 2.745123 | -1.417499 | 2.286117 |
| O | -1.934559 | 0.358486 | 0.61459 | O | 1.655064 | 0.616746 | -0.412186 |
| O | -0.277819 | -0.703286 | -0.644108 | O | 0.263174 | -0.87307 | 0.729785 |
| O | 0.522725 | -3.145446 | 1.844835 | O | 0.309607 | -3.387808 | -1.814247 |
| H | -4.350793 | 0.395269 | -1.658113 | H | 3.825852 | 1.254777 | 2.011783 |
| H | 2.955904 | 2.19341 | 0.258054 | H | -4.544741 | -0.628837 | -0.76163 |
| H | -1.613804 | -2.964825 | 0.31219 | H | 2.213989 | -2.67819 | -0.135471 |
| H | -2.236847 | -1.970204 | 1.653317 | H | 2.637932 | -1.525722 | -1.426835 |
| H | 0.01575 | -1.162318 | 2.165603 | H | 0.293279 | -1.335675 | -2.09733 |
| H | 6.23848 | 2.406049 | 1.136356 | H | -6.41661 | 2.038272 | 0.233939 |
| H | 6.152161 | 3.141598 | -0.458983 | H | -5.437218 | 2.64009 | -1.097475 |
| H | 4.838673 | 3.411699 | 0.705673 | H | -6.101411 | 0.994178 | -1.16793 |
| H | 6.368822 | 0.775956 | -1.574931 | H | -3.489234 | 3.132392 | 0.574581 |
| H | 6.479809 | 0.056969 | 0.025776 | H | -4.453784 | 2.512661 | 1.907589 |
| H | 5.245272 | -0.511939 | -1.10958 | H | -2.852248 | 1.826878 | 1.588807 |
| H | 1.643682 | -2.157777 | -1.684024 | H | -1.251434 | -2.807824 | 1.635655 |
| H | 0.464023 | -3.267768 | -0.971574 | H | 0.207238 | -3.559217 | 0.97613 |
| H | 2.069191 | -3.097069 | -0.235773 | H | -1.347839 | -3.784974 | 0.153709 |
| H | -2.374657 | 3.521302 | -0.563269 | H | 2.684708 | 2.152252 | -2.427634 |
| H | -1.542535 | 2.969081 | 0.90539 | H | 4.320703 | 2.074421 | -1.741967 |
| H | -2.856152 | 4.157655 | 1.021836 | H | 3.595806 | 3.64598 | -2.131267 |
| H | -4.868959 | 2.79312 | -0.967171 | H | 0.726545 | 3.117833 | -0.955041 |
| H | -5.282271 | 3.435987 | 0.628752 | H | 1.695016 | 4.58485 | -0.71098 |
| H | -5.668232 | 1.752919 | 0.229546 | H | 1.084451 | 3.682482 | 0.689424 |
| H | 1.893523 | 0.607088 | -1.366135 | H | -2.242561 | -0.096333 | 1.244705 |
| H | 3.202569 | -0.547683 | -1.153244 | H | -3.093693 | -1.621809 | 1.040446 |
| H | 1.352654 | 0.389958 | 1.110585 | H | -1.47395 | -0.218669 | -1.152567 |
| H | 2.528702 | -0.917051 | 1.213584 | H | -2.254933 | -1.789061 | -1.316772 |
| H | -3.651435 | 1.918981 | 1.721179 | H | 3.520869 | 3.239264 | 0.325463 |
| H | 0.123266 | -3.338705 | 2.707628 | H | 0.800339 | -3.450075 | -2.648761 |
| 4*R*,6*R*,7*S*-**1 I** | | | | 4*R*,6*R*,7*S*-**1 J** | | | |
| C | -3.73161 | -0.248565 | -0.937964 | C | -3.777322 | -0.219013 | -0.605976 |
| C | -3.188824 | 0.805253 | -0.263351 | C | -3.074128 | 0.880139 | -0.209021 |
| C | -2.784032 | -1.331698 | -0.901325 | C | -2.901504 | -1.358123 | -0.51903 |
| C | 3.942942 | 1.136286 | 0.33404 | C | 4.162142 | 0.459159 | -0.876524 |
| C | 5.146418 | 1.319794 | -0.232934 | C | 4.899241 | 1.368515 | -0.218313 |
| C | -1.216852 | -1.566152 | 1.168327 | C | -1.049983 | -1.387994 | 1.31354 |
| C | 0.274904 | -1.240376 | 1.338279 | C | 0.468436 | -1.18239 | 1.203294 |
| C | -1.565813 | -0.80254 | -0.100348 | C | -1.544302 | -0.811359 | -0.004673 |
| C | 0.785298 | -1.128125 | -0.124046 | C | 0.743443 | -1.367293 | -0.313014 |
| C | 5.811442 | 2.676817 | -0.207132 | C | 5.797777 | 2.323177 | -0.970511 |
| C | 5.949588 | 0.247349 | -0.929018 | C | 4.927994 | 1.540096 | 1.281567 |
| C | 1.293291 | -2.456128 | -0.685352 | C | 1.035794 | -2.818405 | -0.695706 |
| C | -5.017924 | 2.469641 | -0.762484 | C | -4.858272 | 2.590019 | -0.717364 |
| C | -3.967976 | 2.326424 | 1.555125 | C | -3.455842 | 2.74505 | 1.405262 |
| C | 3.118948 | -0.122854 | 0.412494 | C | 3.213791 | -0.568992 | -0.316646 |
| C | 1.774171 | 0.028737 | -0.330494 | C | 1.782394 | -0.378501 | -0.862027 |
| C | -3.743595 | 2.16667 | 0.034645 | C | -3.491839 | 2.31474 | -0.078397 |
| O | -2.831333 | -2.468702 | -1.357689 | O | -3.085979 | -2.545974 | -0.76071 |
| O | -1.952922 | 0.574995 | 0.240586 | O | -1.790652 | 0.630526 | 0.144488 |
| O | -0.421832 | -0.7799 | -0.88975 | O | -0.537304 | -1.011323 | -0.943136 |
| O | 1.011399 | -2.222686 | 2.050878 | O | 1.241528 | -2.095261 | 1.96729 |
| H | -4.695389 | -0.266578 | -1.42516 | H | -4.804459 | -0.233331 | -0.939723 |
| H | 3.473806 | 2.005905 | 0.799712 | H | 4.239241 | 0.449225 | -1.9661 |
| H | -1.342555 | -2.637954 | 0.980097 | H | -1.27834 | -2.458985 | 1.338407 |
| H | -1.837885 | -1.276905 | 2.018969 | H | -1.505659 | -0.90492 | 2.180652 |
| H | 0.378904 | -0.261724 | 1.826662 | H | 0.715355 | -0.149269 | 1.483892 |
| H | 6.787668 | 2.629158 | 0.295963 | H | 5.526268 | 3.367879 | -0.762796 |
| H | 6.007335 | 3.040209 | -1.225871 | H | 6.8454 | 2.209139 | -0.657684 |
| H | 5.198957 | 3.423178 | 0.308907 | H | 5.747159 | 2.166678 | -2.05282 |
| H | 6.169873 | 0.542793 | -1.964196 | H | 5.944683 | 1.383885 | 1.6685 |
| H | 6.921538 | 0.110265 | -0.434747 | H | 4.648024 | 2.566299 | 1.557219 |
| H | 5.44827 | -0.722809 | -0.956865 | H | 4.260196 | 0.856174 | 1.810272 |
| H | 1.546271 | -2.330729 | -1.743533 | H | 1.142437 | -2.895022 | -1.783026 |
| H | 0.524603 | -3.230987 | -0.607018 | H | 0.215847 | -3.473618 | -0.386375 |
| H | 2.181713 | -2.799086 | -0.15019 | H | 1.955177 | -3.175916 | -0.226635 |
| H | -5.832266 | 1.792484 | -0.480755 | H | -5.655734 | 2.045464 | -0.199019 |
| H | -4.851094 | 2.376654 | -1.840561 | H | -4.875096 | 2.301154 | -1.773329 |
| H | -5.347729 | 3.492747 | -0.556522 | H | -5.087485 | 3.658376 | -0.654051 |
| H | -4.7414 | 1.634195 | 1.906709 | H | -4.200787 | 2.190503 | 1.987107 |
| H | -4.297516 | 3.34727 | 1.773598 | H | -3.684976 | 3.812552 | 1.485147 |
| H | -3.049012 | 2.132564 | 2.115468 | H | -2.470793 | 2.568929 | 1.846389 |
| H | 3.667729 | -0.980023 | 0.01115 | H | 3.569552 | -1.570137 | -0.599253 |
| H | 2.917309 | -0.355681 | 1.467866 | H | 3.198562 | -0.549011 | 0.777292 |
| H | 1.962907 | 0.138247 | -1.405861 | H | 1.79338 | -0.463104 | -1.95668 |
| H | 1.277856 | 0.952073 | -0.004114 | H | 1.437142 | 0.636664 | -0.629288 |
| H | -2.961598 | 2.881284 | -0.259814 | H | -2.727033 | 2.899572 | -0.609495 |
| H | 0.766963 | -2.149415 | 2.987118 | H | 1.164259 | -1.835877 | 2.899173 |
| 4*R*,6*R*,7*S*-**1 K** | | | | 4*R*,6*R*,7*S*-**1 L** | | | |
| C | -3.376664 | 0.179729 | -1.255203 | C | -3.561098 | 0.455299 | -0.836537 |
| C | -2.958121 | 0.939018 | -0.202511 | C | -2.705884 | 1.301987 | -0.195921 |
| C | -2.497284 | -0.955693 | -1.358044 | C | -2.97236 | -0.857792 | -0.823793 |
| C | 3.582758 | 1.403924 | -0.177046 | C | 3.391857 | 0.692186 | -0.805993 |
| C | 4.922738 | 1.493178 | -0.164729 | C | 4.505606 | 1.112781 | -0.184441 |
| C | -1.440686 | -1.923006 | 0.819894 | C | -1.46362 | -1.547185 | 1.184674 |
| C | -0.016499 | -1.798137 | 1.38139 | C | 0.057697 | -1.754125 | 1.24954 |
| C | -1.46883 | -0.800706 | -0.207566 | C | -1.619397 | -0.707234 | -0.07731 |
| C | 0.811508 | -1.346326 | 0.147828 | C | 0.454603 | -1.846701 | -0.244657 |
| C | 5.607368 | 2.745419 | 0.333036 | C | 5.701095 | 1.584536 | -0.979961 |
| C | 5.868216 | 0.409905 | -0.625772 | C | 4.695465 | 1.16729 | 1.312577 |
| C | 1.363179 | -2.526791 | -0.652309 | C | 0.304723 | -3.26745 | -0.798251 |
| C | -4.578627 | 2.844745 | -0.528023 | C | -2.891967 | 3.03349 | 1.60064 |
| C | -4.049911 | 1.952141 | 1.799904 | C | -1.707396 | 3.563734 | -0.592599 |
| C | 2.718088 | 0.253942 | -0.624265 | C | 2.107906 | 0.185225 | -0.202076 |
| C | 1.886156 | -0.321499 | 0.539825 | C | 1.847165 | -1.287638 | -0.57896 |
| C | -3.514285 | 2.207384 | 0.373066 | C | -2.843672 | 2.76829 | 0.081646 |
| O | -2.481796 | -1.903549 | -2.135585 | O | -3.367104 | -1.932775 | -1.262547 |
| O | -1.870537 | 0.454995 | 0.443752 | O | -1.572206 | 0.721076 | 0.262515 |
| O | -0.176391 | -0.670432 | -0.705236 | O | -0.545801 | -1.00893 | -0.914274 |
| O | 0.517704 | -2.994067 | 1.924879 | O | 0.472656 | -2.92091 | 1.941324 |
| H | -4.210725 | 0.395954 | -1.906688 | H | -4.507519 | 0.730306 | -1.280672 |
| H | 3.022916 | 2.265449 | 0.193903 | H | 3.398702 | 0.699554 | -1.898275 |
| H | -1.555708 | -2.880625 | 0.30084 | H | -1.964065 | -2.510052 | 1.038118 |
| H | -2.228095 | -1.825769 | 1.570505 | H | -1.886601 | -1.056125 | 2.06368 |
| H | 0.005327 | -0.997459 | 2.1332 | H | 0.520565 | -0.865892 | 1.695702 |
| H | 6.270465 | 2.522652 | 1.180941 | H | 5.958792 | 2.623034 | -0.727917 |
| H | 6.243493 | 3.1853 | -0.448149 | H | 6.59126 | 0.981856 | -0.750359 |
| H | 4.887864 | 3.505679 | 0.6537 | H | 5.522393 | 1.530723 | -2.058676 |
| H | 6.49947 | 0.771982 | -1.449333 | H | 5.564131 | 0.564475 | 1.611894 |
| H | 6.553451 | 0.1272 | 0.185411 | H | 4.907383 | 2.195103 | 1.638918 |
| H | 5.360299 | -0.49542 | -0.966066 | H | 3.82998 | 0.809043 | 1.874872 |
| H | 1.786676 | -2.172092 | -1.595956 | H | 0.485284 | -3.256171 | -1.878003 |
| H | 0.571389 | -3.244514 | -0.886238 | H | -0.704392 | -3.653659 | -0.626695 |
| H | 2.143824 | -3.046121 | -0.08948 | H | 1.02185 | -3.946114 | -0.329155 |
| H | -5.458531 | 2.197949 | -0.620417 | H | -1.957833 | 2.725078 | 2.080912 |
| H | -4.190678 | 3.042725 | -1.532553 | H | -3.717924 | 2.49162 | 2.073289 |
| H | -4.906749 | 3.795885 | -0.097101 | H | -3.035439 | 4.10339 | 1.783462 |
| H | -4.904206 | 1.266191 | 1.775446 | H | -0.732983 | 3.277849 | -0.183733 |
| H | -4.381501 | 2.896395 | 2.24355 | H | -1.852716 | 4.634536 | -0.416495 |
| H | -3.277698 | 1.52048 | 2.442787 | H | -1.690258 | 3.394234 | -1.674317 |
| H | 2.022133 | 0.60761 | -1.398326 | H | 2.117244 | 0.303159 | 0.885674 |
| H | 3.319669 | -0.536268 | -1.082549 | H | 1.268976 | 0.787884 | -0.574938 |
| H | 1.38357 | 0.50327 | 1.061932 | H | 2.589969 | -1.929768 | -0.088441 |
| H | 2.553587 | -0.796586 | 1.271315 | H | 2.000512 | -1.409582 | -1.659327 |
| H | -2.662348 | 2.897017 | 0.459741 | H | -3.798041 | 3.079966 | -0.357835 |
| H | 0.085651 | -3.145818 | 2.780265 | H | 0.366029 | -2.752288 | 2.890712 |

Key transitions, oscillator strengths, and rotatory strengths in the ECD of conformers 4*R*,6*R*,7*S*-**1 D** at B3LYP /6-311++G (d,p) level.

| Species | Excited State | *ΔE(eV)^a^* | *λ(nm)^b^* | *f*^c^ | *R_vel_^d^* |
| --- | --- | --- | --- | --- | --- |
| 4*R*,6*R*,7*S*-**1 D** | 78->81 | 3.8966 | 318.18 | 0.0006 | -7.0181 |
|  | 80->81 | 3.9759 | 311.84 | 0.0006 | -1.4303 |
|  | 79->81 | 4.6632 | 265.88 | 0.1506 | 59.8718 |
|  | 77->81 | 4.9187 | 252.06 | 0.0034 | 9.9231 |
|  | 76->81 | 5.4217 | 228.68 | 0.1204 | -63.8299 |
|  | 80->82 | 5.4446 | 227.72 | 0.0031 | -2.5497 |
|  | 80->84 | 5.8242 | 212.88 | 0.0218 | 0.932 |
|  | 80->84 | 5.885 | 210.68 | 0.0144 | 1.7278 |
|  | 80->85 | 6.0344 | 205.46 | 0.0174 | 14.6255 |
|  | 79->82 | 6.2138 | 199.53 | 0.0031 | -19.8439 |
|  | 80->86 | 6.2209 | 199.3 | 0.0594 | -17.7584 |
|  | 75->81 | 6.2889 | 197.15 | 0.0094 | -10.3817 |
|  | 80->88 | 6.3295 | 195.88 | 0.0318 | -22.9699 |
|  | 80->87 | 6.3538 | 195.13 | 0.131 | -15.2228 |
|  | 78->82 | 6.3705 | 194.62 | 0.0069 | 17.9113 |
|  | 74->81 | 6.429 | 192.85 | 0.0116 | 17.2683 |
|  | 80->89 | 6.4672 | 191.71 | 0.0291 | -6.6818 |
|  | 80->90 | 6.5396 | 189.59 | 0.0076 | -2.8977 |
|  | 79->83 | 6.5596 | 189.01 | 0.0055 | 1.3672 |
|  | 73->81 | 6.5815 | 188.38 | 0.0413 | 0.9492 |
|  | 79->84 | 6.6214 | 187.25 | 0.009 | 4.3526 |
|  | 80->91 | 6.6525 | 186.37 | 0.0041 | -7.5481 |
|  | 78->83 | 6.6772 | 185.68 | 0.0011 | 4.2021 |
|  | 79->85 | 6.6967 | 185.14 | 0.0154 | 13.594 |
|  | 72->81 | 6.7097 | 184.78 | 0.0134 | -15.9481 |
|  | 80->93 | 6.7414 | 183.92 | 0.0049 | -10.7108 |
|  | 78->84 | 6.7637 | 183.31 | 0.0021 | -2.5396 |
|  | 70->81 | 6.7954 | 182.45 | 0.0356 | -6.4896 |
|  | 77->82 | 6.8189 | 181.82 | 0.0142 | 11.3139 |
|  | 80->92 | 6.8287 | 181.56 | 0.0733 | 12.3817 |
|  | 78->85 | 6.8382 | 181.31 | 0.0209 | -6.7061 |
|  | 79->86 | 6.8517 | 180.95 | 0.0087 | 18.3216 |
|  | 71->81 | 6.8931 | 179.87 | 0.0222 | 6.5891 |
|  | 80->94 | 6.9493 | 178.41 | 0.0016 | -0.1772 |
|  | 78->86 | 6.9772 | 177.7 | 0.0066 | -9.3975 |
|  | 79->87 | 7.005 | 176.99 | 0.0055 | 10.7618 |
|  | 79->88 | 7.0216 | 176.58 | 0.0112 | 5.5378 |
|  | 80->95 | 7.0302 | 176.36 | 0.0156 | -11.2252 |
|  | 80->96 | 7.0867 | 174.95 | 0.0516 | 6.4582 |
|  | 69->81 | 7.1003 | 174.62 | 0.0119 | -0.3727 |

*^a^*Excitation energy. *^b^*Wavelength. *^c^*Oscillator strength. *^d^*Rotatory strength in velocity form (10^-40^cgs.).

Optimized geometries of predominant conformers for compound 4*S*,6*S*,7*R*-**2** at the B3LYP/6-31G (d,p) level in methanol solution.


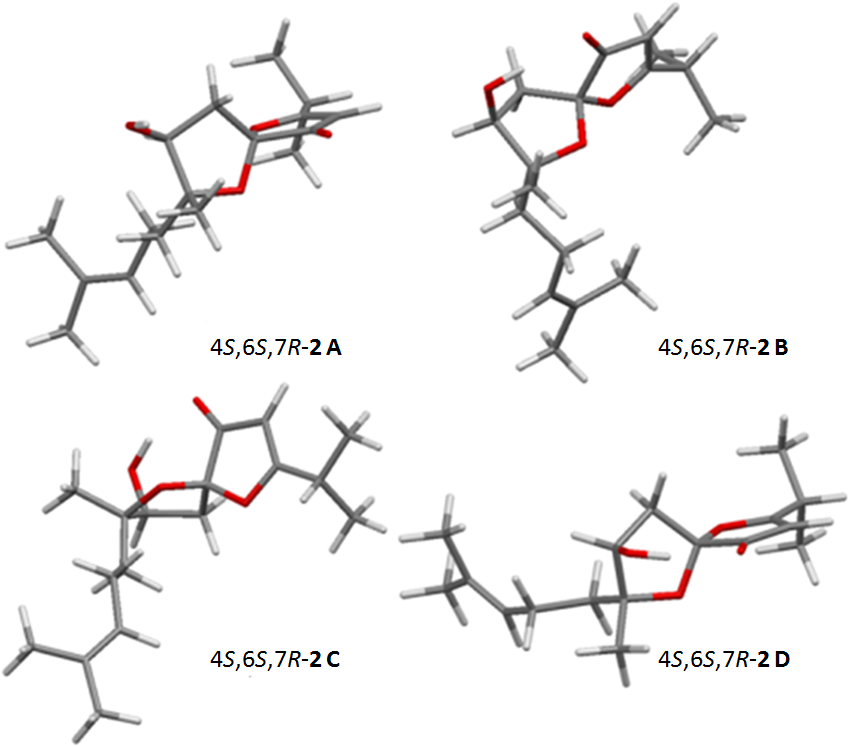


Important thermodynamic parameters (a.u.) and Boltzmann distributions of the optimized compound 4*S*,6*S*,7*R*-**2** at B3LYP/6-31G(d,p) level in methanol solution.

| Conformations | E+ZPE | G | % |
| --- | --- | --- | --- |
| 4*S*,6*S*,7*R*-**2 A** | -963.943275 | -963.998451 | 1.13 |
| 4*S*,6*S*,7*R*-**2 B** | -963.948581 | -964.002190 | 59.27 |
| 4*S*,6*S*,7*R*-**2 C** | -963.948185 | -964.001137 | 19.43 |
| 4*S*,6*S*,7*R*-**2 D** | -963.947738 | -964.001172 | 20.17 |

E+ZPE, G: total energy with zero point energy (ZPE) and Gibbs free energy in methanol solution at B3LYP/6-31G (d,p) level. %: Boltzmann distributions, using the relative Gibbs free energies as weighting factors.

Optimized Z-matrixes of compound 4*S*,6*S*,7*R*-**2** in methanol solution (Å) at B3LYP/6-31G (d,p) level.

| 4*S*,6*S*,7*R*-**2 A** | | | | 4*S*,6*S*,7*R*-**2 B** | | | |
| --- | --- | --- | --- | --- | --- | --- | --- |
| C | 3.53293 | 0.548527 | -0.805212 | C | 3.191833 | 1.016655 | -0.60835 |
| C | 2.630469 | 1.365077 | -0.190674 | C | 2.133924 | 1.76376 | -0.17246 |
| C | 3.002283 | -0.788661 | -0.78387 | C | 2.85113 | -0.359493 | -0.408832 |
| C | -3.381177 | 0.614819 | -0.827004 | C | -3.768913 | -0.569643 | -0.166509 |
| C | -4.464694 | 1.061019 | -0.170737 | C | -4.291806 | 0.65514 | 0.007852 |
| C | 1.485169 | -1.518437 | 1.202396 | C | 1.264303 | -1.102302 | 1.508972 |
| C | -0.026771 | -1.78709 | 1.249785 | C | 0.756663 | -2.484956 | 1.065928 |
| C | 1.629741 | -0.686746 | -0.065504 | C | 1.415212 | -0.369754 | 0.170909 |
| C | -0.409525 | -1.883561 | -0.256957 | C | -0.160024 | -2.117256 | -0.137746 |
| C | -5.701013 | 1.491442 | -0.926232 | C | -5.68036 | 0.826927 | 0.579701 |
| C | -4.579177 | 1.186681 | 1.329722 | C | -3.595616 | 1.950851 | -0.333083 |
| C | -0.243484 | -3.306169 | -0.801332 | C | -0.227617 | -3.200202 | -1.208272 |
| C | 1.539634 | 3.574689 | -0.634269 | C | 0.788419 | 3.696552 | -1.013137 |
| C | 2.711993 | 3.127325 | 1.583947 | C | 1.803004 | 3.740259 | 1.325497 |
| C | -2.06598 | 0.142253 | -0.263913 | C | -2.415603 | -0.94705 | -0.710804 |
| C | -1.807486 | -1.341729 | -0.595484 | C | -1.55815 | -1.675455 | 0.344941 |
| C | 2.699075 | 2.839419 | 0.068344 | C | 1.971193 | 3.25142 | -0.128167 |
| O | 3.450331 | -1.852348 | -1.198025 | O | 3.479962 | -1.402228 | -0.610314 |
| O | 1.51533 | 0.740784 | 0.256994 | O | 1.086625 | 1.043587 | 0.29457 |
| O | 0.589982 | -1.045146 | -0.923345 | O | 0.545118 | -0.974584 | -0.741265 |
| O | -0.42334 | -2.89298 | 2.039783 | O | 1.826666 | -3.351567 | 0.727279 |
| H | 4.474266 | 0.858983 | -1.236559 | H | 4.114506 | 1.404442 | -1.016288 |
| H | -3.441985 | 0.571314 | -1.916768 | H | -4.386555 | -1.420808 | 0.128995 |
| H | 1.871198 | -1.006176 | 2.085412 | H | 0.532098 | -0.574452 | 2.124967 |
| H | 2.039149 | -2.455801 | 1.069932 | H | 2.199903 | -1.171622 | 2.068136 |
| H | -0.530739 | -0.928915 | 1.700606 | H | 0.187243 | -2.988666 | 1.851859 |
| H | -5.577542 | 1.385093 | -2.008791 | H | -6.332904 | 1.365173 | -0.122322 |
| H | -6.57592 | 0.899575 | -0.621989 | H | -6.149845 | -0.134565 | 0.811411 |
| H | -5.949549 | 2.540692 | -0.712345 | H | -5.657532 | 1.426672 | 1.50063 |
| H | -4.775201 | 2.229198 | 1.617005 | H | -4.17881 | 2.521116 | -1.069571 |
| H | -5.431218 | 0.600165 | 1.700525 | H | -3.514909 | 2.590229 | 0.557048 |
| H | -3.686157 | 0.85444 | 1.864099 | H | -2.590819 | 1.809688 | -0.737907 |
| H | -0.973425 | -3.986664 | -0.351625 | H | -0.748101 | -4.081571 | -0.818648 |
| H | 0.7651 | -3.691031 | -0.613478 | H | 0.776638 | -3.500271 | -1.512331 |
| H | -0.399641 | -3.304784 | -1.884373 | H | -0.767896 | -2.843363 | -2.089552 |
| H | 0.572415 | 3.250573 | -0.237038 | H | -0.155361 | 3.285181 | -0.641126 |
| H | 1.547796 | 3.391181 | -1.713794 | H | 0.921847 | 3.370983 | -2.049979 |
| H | 1.63434 | 4.652984 | -0.469788 | H | 0.714853 | 4.788757 | -1.003693 |
| H | 2.809456 | 4.204634 | 1.75294 | H | 1.729976 | 4.832442 | 1.338039 |
| H | 3.551425 | 2.626178 | 2.077309 | H | 2.656338 | 3.44706 | 1.945837 |
| H | 1.783428 | 2.788494 | 2.0544 | H | 0.892803 | 3.3296 | 1.773918 |
| H | -1.250064 | 0.736239 | -0.696452 | H | -2.557134 | -1.60599 | -1.579057 |
| H | -2.027635 | 0.302915 | 0.817753 | H | -1.864855 | -0.073227 | -1.068709 |
| H | -1.977795 | -1.499672 | -1.668666 | H | -2.087949 | -2.570499 | 0.697451 |
| H | -2.540817 | -1.968731 | -0.071643 | H | -1.456987 | -1.017169 | 1.216294 |
| H | 3.645657 | 3.186883 | -0.361037 | H | 2.894384 | 3.677732 | -0.536509 |
| H | 0.073102 | -3.6734 | 1.743071 | H | 2.456533 | -2.84127 | 0.170316 |
| 4*S*,6*S*,7*R*-**2 C** | | | | 4*S*,6*S*,7*R*-**2 D** | | | |
| C | 3.191833 | 1.016655 | -0.60835 | C | 3.798604 | -0.387069 | 0.003581 |
| C | 2.133924 | 1.76376 | -0.17246 | C | 3.220732 | 0.851359 | -0.00381 |
| C | 2.85113 | -0.35949 | -0.40883 | C | 2.743798 | -1.347475 | 0.123999 |
| C | -3.76891 | -0.56964 | -0.16651 | C | -4.035894 | 0.677087 | -0.735618 |
| C | -4.29181 | 0.65514 | 0.007852 | C | -4.844404 | 1.274035 | 0.155067 |
| C | 1.264303 | -1.1023 | 1.508972 | C | 0.539779 | -0.783222 | 1.377513 |
| C | 0.756663 | -2.48496 | 1.065928 | C | -0.466145 | -1.819897 | 0.852467 |
| C | 1.415212 | -0.36975 | 0.170909 | C | 1.423256 | -0.539136 | 0.148965 |
| C | -0.16002 | -2.11726 | -0.13775 | C | -0.670973 | -1.354951 | -0.617383 |
| C | -5.68036 | 0.826927 | 0.579701 | C | -5.650994 | 2.492057 | -0.232725 |
| C | -3.59562 | 1.950851 | -0.33308 | C | -5.045283 | 0.825134 | 1.582582 |
| C | -0.22762 | -3.2002 | -1.20827 | C | -0.971357 | -2.490996 | -1.58816 |
| C | 0.788419 | 3.696552 | -1.01314 | C | 3.388975 | 2.965492 | -1.331009 |
| C | 1.803004 | 3.740259 | 1.325497 | C | 3.579873 | 3.006696 | 1.210998 |
| C | -2.4156 | -0.94705 | -0.7108 | C | -3.155107 | -0.531576 | -0.548962 |
| C | -1.55815 | -1.67546 | 0.344941 | C | -1.664481 | -0.182448 | -0.752591 |
| C | 1.971193 | 3.25142 | -0.12817 | C | 3.861024 | 2.202926 | -0.075963 |
| O | 3.479962 | -1.40223 | -0.61031 | O | 2.755532 | -2.579106 | 0.205297 |
| O | 1.086625 | 1.043587 | 0.29457 | O | 1.86969 | 0.845829 | 0.081349 |
| O | 0.545118 | -0.97458 | -0.74127 | O | 0.662619 | -0.842346 | -0.983669 |
| O | 1.826666 | -3.35157 | 0.727279 | O | 0.033015 | -3.143268 | 0.96406 |
| H | 4.114506 | 1.404442 | -1.01629 | H | 4.85816 | -0.590924 | -0.058722 |
| H | -4.38656 | -1.42081 | 0.128995 | H | -3.985741 | 1.114272 | -1.735238 |
| H | 0.532098 | -0.57445 | 2.124967 | H | 0.051143 | 0.153354 | 1.658369 |
| H | 2.199903 | -1.17162 | 2.068136 | H | 1.096584 | -1.154803 | 2.240501 |
| H | 0.187243 | -2.98867 | 1.851859 | H | -1.405439 | -1.799536 | 1.410397 |
| H | -6.3329 | 1.365173 | -0.12232 | H | -5.479613 | 2.782262 | -1.274252 |
| H | -6.14985 | -0.13457 | 0.811411 | H | -6.727166 | 2.311175 | -0.100999 |
| H | -5.65753 | 1.426672 | 1.50063 | H | -5.402242 | 3.350572 | 0.407011 |
| H | -4.17881 | 2.521116 | -1.06957 | H | -4.443137 | -0.044623 | 1.85508 |
| H | -3.51491 | 2.590229 | 0.557048 | H | -4.798052 | 1.637994 | 2.279368 |
| H | -2.59082 | 1.809688 | -0.73791 | H | -6.099822 | 0.574683 | 1.763992 |
| H | -0.7481 | -4.08157 | -0.81865 | H | -1.075686 | -2.10246 | -2.606885 |
| H | 0.776638 | -3.50027 | -1.51233 | H | -1.900533 | -2.999933 | -1.315469 |
| H | -0.7679 | -2.84336 | -2.08955 | H | -0.164853 | -3.226251 | -1.571262 |
| H | -0.15536 | 3.285181 | -0.64113 | H | 3.614306 | 2.406547 | -2.245166 |
| H | 0.921847 | 3.370983 | -2.04998 | H | 3.900103 | 3.931999 | -1.385645 |
| H | 0.714853 | 4.788757 | -1.00369 | H | 2.310311 | 3.148021 | -1.295337 |
| H | 1.729976 | 4.832442 | 1.338039 | H | 4.092118 | 3.972796 | 1.158671 |
| H | 2.656338 | 3.44706 | 1.945837 | H | 3.939392 | 2.476027 | 2.098748 |
| H | 0.892803 | 3.3296 | 1.773918 | H | 2.507614 | 3.191934 | 1.329854 |
| H | -2.55713 | -1.60599 | -1.57906 | H | -3.304944 | -0.980369 | 0.438338 |
| H | -1.86486 | -0.07323 | -1.06871 | H | -3.446073 | -1.295702 | -1.283039 |
| H | -2.08795 | -2.5705 | 0.697451 | H | -1.387637 | 0.612222 | -0.049437 |
| H | -1.45699 | -1.01717 | 1.216294 | H | -1.526749 | 0.238696 | -1.756774 |
| H | 2.894384 | 3.677732 | -0.53651 | H | 4.940745 | 2.032766 | -0.153425 |
| H | 2.456533 | -2.84127 | 0.170316 | H | 0.961954 | -3.137696 | 0.641321 |

Key transitions, oscillator strengths, and rotatory strengths in the ECD of conformers 4*S*,6*S*,7*R*-**2 B** at B3LYP /6-311++G (d,p) level.

| Species | Excited State | *ΔE(eV)^a^* | *λ(nm)^b^* | *f*^c^ | *R_vel_^d^* |
| --- | --- | --- | --- | --- | --- |
| 4*S*,6*S*,7*R*-**2 B** | 78->81 | 3.9021 | 317.73 | 0.0009 | 8.6422 |
|  | 80->81 | 4.0992 | 302.46 | 0.0002 | 0.2336 |
|  | 79->81 | 4.6789 | 264.99 | 0.1682 | -47.8662 |
|  | 77->81 | 4.9476 | 250.59 | 0.0033 | -10.5332 |
|  | 76->81 | 5.4585 | 227.14 | 0.1159 | 59.3468 |
|  | 80->82 | 5.5053 | 225.21 | 0.0028 | -6.8357 |
|  | 80->83 | 5.9441 | 208.58 | 0.0067 | -2.2029 |
|  | 80->84 | 5.9607 | 208 | 0.0182 | -6.5245 |
|  | 80->85 | 6.05 | 204.93 | 0.0417 | 10.5021 |
|  | 79->82 | 6.2228 | 199.24 | 0.0054 | -2.2317 |
|  | 80->87 | 6.3007 | 196.78 | 0.0141 | -14.4995 |
|  | 80->86 | 6.3664 | 194.75 | 0.0821 | 43.0731 |
|  | 75->81 | 6.3729 | 194.55 | 0.005 | 1.5022 |
|  | 78->82 | 6.4071 | 193.51 | 0.0466 | -36.7681 |
|  | 80->88 | 6.4459 | 192.35 | 0.0884 | 1.9901 |
|  | 74->81 | 6.4652 | 191.77 | 0.0644 | 13.6805 |
|  | 79->83 | 6.5432 | 189.49 | 0.0064 | -2.8948 |
|  | 80->89 | 6.5663 | 188.82 | 0.005 | -2.022 |
|  | 73->81 | 6.5944 | 188.02 | 0.0146 | 14.2121 |
|  | 79->84 | 6.6162 | 187.4 | 0.0005 | -1.1123 |
|  | 80->90 | 6.6547 | 186.31 | 0.0102 | 0.8062 |
|  | 78->83 | 6.6879 | 185.38 | 0.0074 | 7.4783 |
|  | 72->81 | 6.7104 | 184.76 | 0.0056 | -10.3629 |
|  | 79->85 | 6.7287 | 184.26 | 0.0128 | -13.3033 |
|  | 78->84 | 6.7516 | 183.64 | 0.0209 | 21.8064 |
|  | 80->91 | 6.7661 | 183.24 | 0.0334 | -28.4391 |
|  | 71->81 | 6.7819 | 182.82 | 0.0235 | 12.728 |
|  | 77->82 | 6.8319 | 181.48 | 0.0056 | -12.4117 |
|  | 80->93 | 6.8359 | 181.37 | 0.0039 | -17.2109 |
|  | 80->92 | 6.842 | 181.21 | 0.0056 | -8.5973 |
|  | 71->81 | 6.861 | 180.71 | 0.0813 | 44.3944 |
|  | 78->85 | 6.8883 | 179.99 | 0.0028 | -1.0137 |
|  | 79->86 | 6.9011 | 179.66 | 0.0038 | 3.4037 |
|  | 79->88 | 6.9788 | 177.66 | 0.0033 | 4.8357 |
|  | 79->87 | 7.0038 | 177.02 | 0.0014 | -0.2475 |
|  | 79->88 | 7.0153 | 176.73 | 0.0041 | 1.38 |
|  | 80->94 | 7.036 | 176.21 | 0.0215 | -3.8818 |
|  | 78->86 | 7.0579 | 175.67 | 0.0342 | -37.4639 |
|  | 80->95 | 7.0859 | 174.97 | 0.0539 | 21.3158 |
|  | 69->81 | 7.1032 | 174.55 | 0.0045 | -4.2947 |

*^a^*Excitation energy. *^b^*Wavelength. *^c^*Oscillator strength. *^d^*Rotatory strength in velocity form (10^-40^cgs.).

Optimized geometries of predominant conformers for compound 4*R*,6*S*,7*R*-**2** at the B3LYP/6-31G (d,p) level in methanol solution.


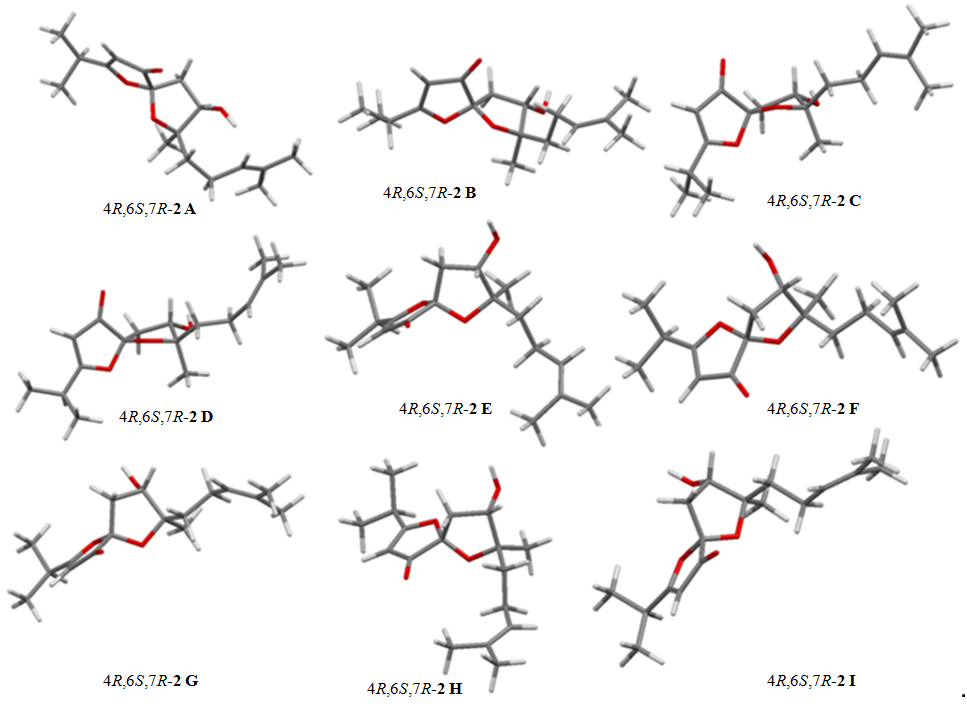


Important thermodynamic parameters (a.u.) and Boltzmann distributions of the optimized compound 4*R*,6*S*,7*R*-**2** at B3LYP/6-31G (d,p) level in methanol solution.

| Conformations | E+ZPE | G | % |
| --- | --- | --- | --- |
| 4*R*,6*S*,7*R*-**2 A** | -963.942996 | -963.995885 | 5.66 |
| 4*R*,6*S*,7*R*-**2 B** | -963.941462 | -963.995437 | 3.52 |
| 4*R*,6*S*,7*R*-**2 C** | -963.941805 | -963.996440 | 10.19 |
| 4*R*,6*S*,7*R*-**2 D** | -963.941674 | -963.996528 | 11.18 |
| 4*R*,6*S*,7*R*-**2 E** | -963.941563 | -963.996194 | 7.85 |
| 4*R*,6*S*,7*R*-**2 F** | -963.942753 | -963.997054 | 19.52 |
| 4*R*,6*S*,7*R*-**2 G** | -963.942805 | -963.996687 | 13.23 |
| 4*R*,6*S*,7*R*-**2 H** | -963.943514 | -963.996374 | 9.50 |
| 4*R*,6*S*,7*R*-**2 I** | -963.943262 | -963.997046 | 19.35 |

E+ZPE, G: total energy with zero point energy (ZPE) and Gibbs free energy in methanol solution at B3LYP/6-31G (d,p) level. %: Boltzmann distributions, using the relative Gibbs free energies as weighting factors.

Optimized Z-matrixes of compound 4*R*,6*S*,7*R*-**2** in methanol solution (Å) at B3LYP/6-31G (d,p) level.

| 4*R*,6*S*,7*R*-**2 A** | | | | 4*R*,6*S*,7*R*-**2 B** | | | |
| --- | --- | --- | --- | --- | --- | --- | --- |
| C | -3.512027 | 1.302979 | 0.328399 | C | -2.793043 | -1.583964 | 0.785785 |
| C | -3.532762 | 0.058172 | -0.226169 | C | -3.317722 | -0.697608 | -0.107196 |
| C | -2.134341 | 1.701165 | 0.460478 | C | -1.477624 | -1.118903 | 1.141721 |
| C | 4.026908 | 0.264855 | 0.750142 | C | 3.894759 | -1.06286 | -0.579553 |
| C | 4.969028 | -0.038855 | -0.164572 | C | 5.181829 | -1.033867 | -0.197109 |
| C | -0.349229 | 0.826305 | -1.24054 | C | -0.959158 | 1.445674 | 1.149001 |
| C | 1.045383 | 0.637464 | -0.632814 | C | 0.519538 | 1.733664 | 0.827347 |
| C | -1.29334 | 0.510773 | -0.074081 | C | -1.250926 | 0.18248 | 0.327984 |
| C | 0.802802 | -0.409798 | 0.492231 | C | 0.681625 | 1.167717 | -0.602496 |
| C | 5.770628 | 1.052962 | -0.836808 | C | 6.228314 | -1.821122 | -0.951859 |
| C | 5.348205 | -1.439846 | -0.581443 | C | 5.719696 | -0.253201 | 0.978157 |
| C | 0.763066 | -1.84509 | -0.036311 | C | 0.184435 | 2.13934 | -1.678788 |
| C | -4.655062 | -2.106087 | 0.301198 | C | -5.44644 | -1.960523 | -0.590257 |
| C | -6.033864 | -0.121084 | -0.508949 | C | -5.456181 | 0.573376 | -0.313713 |
| C | 3.184047 | -0.670536 | 1.58271 | C | 2.711965 | -0.355611 | 0.02958 |
| C | 1.703168 | -0.258226 | 1.729373 | C | 2.090961 | 0.663667 | -0.948894 |
| C | -4.680673 | -0.838665 | -0.582456 | C | -4.655231 | -0.661583 | -0.784304 |
| O | -1.624376 | 2.732879 | 0.882469 | O | -0.63671 | -1.57169 | 1.911308 |
| O | -2.308201 | -0.4594 | -0.493781 | O | -2.500217 | 0.339107 | -0.417539 |
| O | -0.528097 | -0.023763 | 0.965067 | O | -0.191169 | -0.0016 | -0.565209 |
| O | 1.959168 | 0.253187 | -1.641266 | O | 0.880091 | 3.104698 | 0.860719 |
| H | -4.372778 | 1.889598 | 0.614005 | H | -3.27362 | -2.477175 | 1.157011 |
| H | 3.881015 | 1.323242 | 0.977945 | H | 3.649896 | -1.667265 | -1.455996 |
| H | -0.503317 | 1.833509 | -1.633935 | H | -1.14106 | 1.295179 | 2.215909 |
| H | -0.502069 | 0.109824 | -2.052258 | H | -1.587074 | 2.270238 | 0.801025 |
| H | 1.362542 | 1.576103 | -0.155712 | H | 1.15003 | 1.15552 | 1.512541 |
| H | 5.460131 | 2.050297 | -0.510221 | H | 5.797315 | -2.37418 | -1.792668 |
| H | 6.840835 | 0.938514 | -0.61625 | H | 7.013807 | -1.159936 | -1.344655 |
| H | 5.671966 | 1.001365 | -1.929486 | H | 6.733041 | -2.541273 | -0.292362 |
| H | 6.391346 | -1.646638 | -0.305823 | H | 6.19243 | -0.926744 | 1.706672 |
| H | 4.723988 | -2.212967 | -0.128266 | H | 6.503539 | 0.443899 | 0.650529 |
| H | 5.288782 | -1.548013 | -1.672389 | H | 4.955699 | 0.324961 | 1.503042 |
| H | 0.525867 | -2.537361 | 0.778459 | H | 0.202324 | 1.643271 | -2.654631 |
| H | 1.725512 | -2.130212 | -0.469268 | H | 0.825053 | 3.024339 | -1.72401 |
| H | 0.002109 | -1.960029 | -0.813672 | H | -0.840354 | 2.469353 | -1.483812 |
| H | -5.443772 | -2.794397 | -0.019042 | H | -6.385645 | -1.908795 | -1.14995 |
| H | -4.830748 | -1.848125 | 1.351619 | H | -5.694375 | -2.119616 | 0.465346 |
| H | -3.693919 | -2.622494 | 0.227655 | H | -4.886129 | -2.831097 | -0.946516 |
| H | -6.058299 | 0.761381 | -1.156638 | H | -4.891932 | 1.496689 | -0.471629 |
| H | -6.259246 | 0.197923 | 0.515059 | H | -5.699885 | 0.493385 | 0.751709 |
| H | -6.829931 | -0.800396 | -0.82963 | H | -6.393414 | 0.639907 | -0.875348 |
| H | 3.253584 | -1.701461 | 1.223028 | H | 1.941557 | -1.092969 | 0.292076 |
| H | 3.602499 | -0.683097 | 2.600525 | H | 2.995818 | 0.14295 | 0.961233 |
| H | 1.644399 | 0.785447 | 2.065124 | H | 2.037666 | 0.214378 | -1.949336 |
| H | 1.257753 | -0.865898 | 2.526713 | H | 2.748726 | 1.536994 | -1.038488 |
| H | -4.503018 | -1.156559 | -1.619919 | H | -4.446052 | -0.529109 | -1.855676 |
| H | 2.845203 | 0.258298 | -1.229862 | H | 0.935894 | 3.37292 | 1.791311 |
| 4*R*,6*S*,7*R*-**2 C** | | | | 4*R*,6*S*,7*R*-**2 D** | | | |
| C | -3.477951 | -0.984042 | -1.410617 | C | 3.360318 | 1.477972 | -0.589875 |
| C | -3.633776 | 0.111232 | -0.614608 | C | 3.643593 | 0.203386 | -0.198696 |
| C | -2.191064 | -1.558891 | -1.118379 | C | 1.979298 | 1.736295 | -0.273303 |
| C | 4.470559 | -0.314289 | -0.375693 | C | -4.397658 | -0.663477 | -0.925933 |
| C | 5.530981 | 0.488465 | -0.561525 | C | -5.487782 | 0.102804 | -0.759693 |
| C | -1.17052 | -1.333654 | 1.279968 | C | 0.844437 | 0.54348 | 1.759988 |
| C | 0.367796 | -1.244338 | 1.291407 | C | -0.656172 | 0.269652 | 1.542104 |
| C | -1.554403 | -0.645462 | -0.035896 | C | 1.4322 | 0.424102 | 0.348768 |
| C | 0.658122 | 0.021109 | 0.449414 | C | -0.673818 | -0.644614 | 0.293777 |
| C | 6.658567 | 0.076103 | -1.47979 | C | -6.536447 | 0.196835 | -1.84425 |
| C | 5.725278 | 1.835064 | 0.093402 | C | -5.791851 | 0.922311 | 0.471371 |
| C | 0.52906 | 1.317029 | 1.2539 | C | -0.399365 | -2.114202 | 0.623378 |
| C | -5.403927 | 1.011361 | 0.908075 | C | 4.710692 | -1.826957 | -1.183706 |
| C | -4.359315 | 2.490304 | -0.885913 | C | 6.107634 | 0.25776 | -0.737513 |
| C | 3.255435 | -0.087783 | 0.486183 | C | -3.249482 | -0.889349 | 0.023387 |
| C | 1.962792 | -0.0518 | -0.355824 | C | -1.907384 | -0.451656 | -0.599116 |
| C | -4.789749 | 1.058673 | -0.506127 | C | 4.914008 | -0.589389 | -0.28114 |
| O | -1.622666 | -2.552315 | -1.558173 | O | 1.286245 | 2.737455 | -0.415521 |
| O | -2.585855 | 0.368919 | 0.204088 | O | 2.599052 | -0.460143 | 0.356954 |
| O | -0.407583 | -0.038318 | -0.551416 | O | 0.442234 | -0.105637 | -0.483134 |
| O | 0.931596 | -1.129875 | 2.588481 | O | -1.29744 | -0.359415 | 2.640295 |
| H | -4.197528 | -1.350703 | -2.12918 | H | 4.047467 | 2.172609 | -1.050178 |
| H | 4.461835 | -1.26149 | -0.919709 | H | -4.307048 | -1.201562 | -1.872281 |
| H | -1.535146 | -2.362713 | 1.325771 | H | 1.036574 | 1.523939 | 2.202548 |
| H | -1.582277 | -0.779529 | 2.127558 | H | 1.272743 | -0.223394 | 2.410863 |
| H | 0.787183 | -2.111766 | 0.763771 | H | -1.164127 | 1.209199 | 1.285295 |
| H | 7.613901 | 0.032762 | -0.937711 | H | -7.517997 | -0.132356 | -1.474684 |
| H | 6.48027 | -0.904059 | -1.933565 | H | -6.666024 | 1.235727 | -2.179202 |
| H | 6.796369 | 0.807334 | -2.288878 | H | -6.279726 | -0.412801 | -2.716567 |
| H | 5.84969 | 2.618427 | -0.667089 | H | -5.921169 | 1.981037 | 0.207058 |
| H | 4.896978 | 2.127036 | 0.743014 | H | -6.739449 | 0.600328 | 0.925348 |
| H | 6.644416 | 1.842614 | 0.695835 | H | -5.015217 | 0.859932 | 1.236906 |
| H | 0.655548 | 2.176567 | 0.587312 | H | -0.332543 | -2.692048 | -0.304407 |
| H | 1.2881 | 1.367136 | 2.038646 | H | -1.196933 | -2.533991 | 1.241309 |
| H | -0.453241 | 1.395811 | 1.729116 | H | 0.543676 | -2.230708 | 1.165723 |
| H | -6.272529 | 1.676253 | 0.955387 | H | 4.512476 | -1.523 | -2.217823 |
| H | -4.679136 | 1.338869 | 1.660272 | H | 3.873422 | -2.438984 | -0.837216 |
| H | -5.7336 | -0.000848 | 1.164994 | H | 5.616221 | -2.442063 | -1.175623 |
| H | -3.950171 | 2.526549 | -1.90103 | H | 5.965734 | 0.623303 | -1.760925 |
| H | -3.598329 | 2.866253 | -0.194625 | H | 7.018668 | -0.348609 | -0.722278 |
| H | -5.224706 | 3.159542 | -0.841707 | H | 6.262966 | 1.121235 | -0.082548 |
| H | 3.175698 | -0.900392 | 1.22129 | H | -3.402402 | -0.371645 | 0.974616 |
| H | 3.347687 | 0.837343 | 1.062936 | H | -3.197704 | -1.961007 | 0.26365 |
| H | 1.912125 | -0.948416 | -0.987172 | H | -1.961092 | 0.610199 | -0.869373 |
| H | 1.99702 | 0.808527 | -1.036348 | H | -1.741468 | -1.004507 | -1.533407 |
| H | -5.54213 | 0.716289 | -1.225763 | H | 5.106854 | -0.953865 | 0.738481 |
| H | 0.870717 | -1.999167 | 3.015181 | H | -1.407954 | 0.305237 | 3.338678 |
| 4*R*,6*S*,7*R*-**2 E** | | | | 4*R*,6*S*,7*R*-**2 F** | | | |
| C | -2.748823 | -1.296926 | 1.198393 | C | 3.09622 | 1.761376 | 0.014011 |
| C | -3.050605 | -0.867214 | -0.059442 | C | 3.4797 | 0.457032 | -0.100974 |
| C | -1.626692 | -0.525481 | 1.667556 | C | 1.663757 | 1.787183 | 0.144562 |
| C | 4.336306 | -0.251089 | -0.650407 | C | -4.514037 | 0.217969 | -0.718396 |
| C | 4.940906 | -1.360594 | -0.195436 | C | -5.452613 | 0.499047 | 0.199855 |
| C | -1.278615 | 1.932352 | 0.834419 | C | 0.44617 | -0.19069 | 1.31741 |
| C | 0.199808 | 2.34676 | 0.697763 | C | -0.419442 | -1.326291 | 0.750384 |
| C | -1.253332 | 0.432683 | 0.505469 | C | 1.204077 | 0.30553 | 0.090254 |
| C | 0.760922 | 1.328709 | -0.32237 | C | -0.72594 | -0.838453 | -0.687935 |
| C | 6.425902 | -1.368634 | 0.08641 | C | -6.556569 | 1.485488 | -0.10483 |
| C | 4.24949 | -2.675357 | 0.074872 | C | -5.524751 | -0.106067 | 1.581275 |
| C | 0.447251 | 1.719824 | -1.769932 | C | -0.71026 | -1.939155 | -1.744234 |
| C | -4.855823 | -2.559689 | -0.549709 | C | 5.93165 | 0.888921 | -0.514482 |
| C | -5.124169 | -0.117475 | -1.229107 | C | 5.168776 | -0.991729 | 1.027584 |
| C | 2.88216 | -0.037116 | -0.981781 | C | -3.341057 | -0.72267 | -0.612019 |
| C | 2.247411 | 1.041231 | -0.080205 | C | -1.999899 | 0.028214 | -0.747326 |
| C | -4.136416 | -1.284743 | -1.005786 | C | 4.843983 | -0.153529 | -0.229249 |
| O | -1.023788 | -0.534764 | 2.734839 | O | 0.876579 | 2.716896 | 0.287384 |
| O | -2.248065 | 0.122383 | -0.523815 | O | 2.463376 | -0.435638 | -0.070819 |
| O | 0.025185 | 0.118927 | 0.03612 | O | 0.386335 | 0.079165 | -1.006579 |
| O | 0.405666 | 3.672735 | 0.241009 | O | 0.287069 | -2.561479 | 0.691164 |
| H | -3.258793 | -2.074828 | 1.747457 | H | 3.747553 | 2.622859 | -0.000227 |
| H | 4.96204 | 0.632218 | -0.797632 | H | -4.583538 | 0.736271 | -1.677198 |
| H | -1.679773 | 2.124145 | 1.832533 | H | -0.178967 | 0.629333 | 1.68652 |
| H | -1.889547 | 2.468382 | 0.103577 | H | 1.107601 | -0.51399 | 2.124667 |
| H | 0.710515 | 2.186871 | 1.657138 | H | -1.344157 | -1.448177 | 1.327613 |
| H | 6.626708 | -1.623743 | 1.136599 | H | -7.544778 | 1.013061 | -0.012534 |
| H | 6.939446 | -2.128969 | -0.519034 | H | -6.546867 | 2.321768 | 0.608432 |
| H | 6.887734 | -0.398388 | -0.122946 | H | -6.469992 | 1.898458 | -1.114984 |
| H | 4.683793 | -3.474354 | -0.542226 | H | -5.505317 | 0.681736 | 2.347031 |
| H | 4.396587 | -2.979772 | 1.120364 | H | -6.472778 | -0.644823 | 1.718175 |
| H | 3.174733 | -2.648745 | -0.118603 | H | -4.710332 | -0.801844 | 1.795798 |
| H | 0.671739 | 0.887804 | -2.442709 | H | -0.928563 | -1.512106 | -2.728936 |
| H | 1.044394 | 2.58564 | -2.070493 | H | -1.458534 | -2.706514 | -1.525925 |
| H | -0.608791 | 1.975909 | -1.895185 | H | 0.269626 | -2.418407 | -1.775216 |
| H | -5.385548 | -2.399896 | 0.396321 | H | 6.041114 | 1.587833 | 0.322588 |
| H | -4.15632 | -3.391199 | -0.415407 | H | 5.709416 | 1.466836 | -1.417502 |
| H | -5.595102 | -2.855673 | -1.300614 | H | 6.894219 | 0.388518 | -0.659776 |
| H | -5.655446 | 0.122983 | -0.3013 | H | 5.248372 | -0.348498 | 1.911323 |
| H | -5.864369 | -0.401044 | -1.984093 | H | 6.125622 | -1.505813 | 0.891194 |
| H | -4.605576 | 0.781429 | -1.573947 | H | 4.396114 | -1.742811 | 1.214352 |
| H | 2.804471 | 0.277408 | -2.03188 | H | -3.366954 | -1.281167 | 0.329303 |
| H | 2.307128 | -0.963396 | -0.889843 | H | -3.415623 | -1.468625 | -1.415443 |
| H | 2.788257 | 1.989964 | -0.196536 | H | -1.9377 | 0.800321 | 0.029692 |
| H | 2.363206 | 0.737984 | 0.967717 | H | -1.978731 | 0.559145 | -1.707407 |
| H | -3.638081 | -1.481967 | -1.965879 | H | 4.783965 | -0.846679 | -1.080555 |
| H | 0.210531 | 4.273538 | 0.977172 | H | 0.473774 | -2.831279 | 1.604475 |
| 4*R*,6*S*,7*R*-**2 G** | | | | 4*R*,6*S*,7*R*-**2 H** | | | |
| C | -3.146836 | 1.755529 | 0.054583 | C | -2.218754 | -1.925979 | 0.137938 |
| C | -3.494685 | 0.458653 | 0.296679 | C | -2.953868 | -0.833196 | -0.219206 |
| C | -1.754656 | 1.77543 | -0.308126 | C | -0.89726 | -1.472457 | 0.478714 |
| C | 4.458898 | 0.279291 | -0.554527 | C | 4.292146 | 0.089922 | -0.540474 |
| C | 5.597862 | 0.484494 | 0.126729 | C | 4.72306 | -1.099081 | -0.088383 |
| C | -0.726157 | -0.258662 | -1.561999 | C | -0.618375 | 0.87783 | 1.560453 |
| C | 0.235396 | -1.35862 | -1.086939 | C | -0.031716 | 2.182795 | 0.998909 |
| C | -1.279475 | 0.298622 | -0.25426 | C | -0.916141 | 0.069669 | 0.299415 |
| C | 0.766199 | -0.79431 | 0.254665 | C | 0.71375 | 1.697418 | -0.269941 |
| C | 6.609014 | 1.500138 | -0.353535 | C | 6.170566 | -1.30279 | 0.294789 |
| C | 5.994973 | -0.237651 | 1.391841 | C | 3.852349 | -2.322716 | 0.076863 |
| C | 0.944398 | -1.839361 | 1.351991 | C | 0.644742 | 2.667333 | -1.445372 |
| C | -5.85093 | 0.910702 | 1.079347 | C | -5.039874 | -2.074905 | -0.909551 |
| C | -5.330722 | -1.051145 | -0.462775 | C | -5.179842 | 0.098375 | 0.415308 |
| C | 3.332704 | -0.672303 | -0.24143 | C | 2.897499 | 0.463929 | -0.974969 |
| C | 2.020665 | 0.081497 | 0.061329 | C | 2.160492 | 1.298949 | 0.092206 |
| C | -4.81421 | -0.145038 | 0.677318 | C | -4.389975 | -0.713538 | -0.635676 |
| O | -1.008845 | 2.696603 | -0.623846 | O | 0.097825 | -2.083289 | 0.857062 |
| O | -2.488916 | -0.433864 | 0.147038 | O | -2.282699 | 0.340018 | -0.167472 |
| O | -0.293063 | 0.129534 | 0.705885 | O | -0.008531 | 0.47602 | -0.667937 |
| O | -0.436575 | -2.591387 | -0.847918 | O | -1.047367 | 3.110394 | 0.631793 |
| H | -3.795487 | 2.615758 | 0.131152 | H | -2.5606 | -2.950469 | 0.14803 |
| H | 4.299517 | 0.880557 | -1.452194 | H | 5.021838 | 0.900407 | -0.594477 |
| H | -0.181211 | 0.543128 | -2.071078 | H | 0.127011 | 0.334663 | 2.150236 |
| H | -1.506483 | -0.628147 | -2.231566 | H | -1.504503 | 1.034324 | 2.179896 |
| H | 1.055421 | -1.506927 | -1.800199 | H | 0.671009 | 2.646339 | 1.703125 |
| H | 6.790141 | 2.269073 | 0.410727 | H | 6.263289 | -1.602238 | 1.348285 |
| H | 7.58122 | 1.026776 | -0.551072 | H | 6.624726 | -2.111037 | -0.296115 |
| H | 6.281883 | 2.001027 | -1.27033 | H | 6.76596 | -0.396453 | 0.143974 |
| H | 6.944945 | -0.771547 | 1.249949 | H | 4.149907 | -3.111923 | -0.628531 |
| H | 6.164041 | 0.479941 | 2.206643 | H | 3.981689 | -2.746604 | 1.082215 |
| H | 5.251233 | -0.961617 | 1.732678 | H | 2.787011 | -2.123829 | -0.061646 |
| H | 1.308441 | -1.358653 | 2.266276 | H | 1.107842 | 2.225319 | -2.331992 |
| H | 1.666439 | -2.604225 | 1.05193 | H | 1.175248 | 3.59467 | -1.205503 |
| H | -0.006017 | -2.331827 | 1.56358 | H | -0.392908 | 2.911878 | -1.677406 |
| H | -6.101986 | 1.562857 | 0.234972 | H | -5.090614 | -2.681434 | 0.001954 |
| H | -5.490346 | 1.53737 | 1.901618 | H | -4.48822 | -2.640097 | -1.667856 |
| H | -6.772189 | 0.418286 | 1.406344 | H | -6.062698 | -1.930093 | -1.271428 |
| H | -5.549257 | -0.459156 | -1.358761 | H | -5.221623 | -0.439622 | 1.369253 |
| H | -6.253299 | -1.549541 | -0.148446 | H | -6.205363 | 0.254815 | 0.06576 |
| H | -4.595182 | -1.816946 | -0.72432 | H | -4.720446 | 1.075469 | 0.588889 |
| H | 3.180474 | -1.334124 | -1.107344 | H | 2.965344 | 1.039441 | -1.907793 |
| H | 3.587808 | -1.322343 | 0.600191 | H | 2.299504 | -0.422848 | -1.198661 |
| H | 1.82318 | 0.807812 | -0.737794 | H | 2.723705 | 2.217998 | 0.301874 |
| H | 2.149248 | 0.667372 | 0.979354 | H | 2.148827 | 0.723229 | 1.02604 |
| H | -4.610419 | -0.790676 | 1.543673 | H | -4.384709 | -0.128535 | -1.566706 |
| H | -0.777545 | -2.904914 | -1.700601 | H | -1.507834 | 3.372132 | 1.444884 |
| 4*R*,6*S*,7*R*-**2 I** | | | |  | | | |
| C | 2.694694 | -1.854979 | -0.185185 |  | | | |
| C | 3.236977 | -0.696736 | 0.290006 |  | | | |
| C | 1.361631 | -1.559994 | -0.638974 |  | | | |
| C | -3.985073 | -0.919777 | -0.0671 |  | | | |
| C | -5.29299 | -0.796261 | 0.211894 |  | | | |
| C | 0.849018 | 0.788779 | -1.628822 |  | | | |
| C | 0.019384 | 1.955402 | -1.06975 |  | | | |
| C | 1.150774 | -0.043405 | -0.384471 |  | | | |
| C | -0.750921 | 1.290432 | 0.09923 |  | | | |
| C | -6.324588 | -1.583944 | -0.562682 |  | | | |
| C | -5.870162 | 0.097929 | 1.283069 |  | | | |
| C | -0.94169 | 2.190913 | 1.315889 |  | | | |
| C | 5.401544 | -1.690612 | 1.12309 |  | | | |
| C | 5.361416 | 0.561383 | -0.071009 |  | | | |
| C | -2.813315 | -0.226755 | 0.578514 |  | | | |
| C | -2.077099 | 0.692923 | -0.416742 |  | | | |
| C | 4.597906 | -0.412963 | 0.853776 |  | | | |
| O | 0.498282 | -2.269151 | -1.14546 |  | | | |
| O | 2.419348 | 0.379728 | 0.221539 |  | | | |
| O | 0.11521 | 0.174552 | 0.514251 |  | | | |
| O | 0.843868 | 3.001167 | -0.565972 |  | | | |
| H | 3.173041 | -2.82312 | -0.205433 |  | | | |
| H | -3.710686 | -1.601129 | -0.875578 |  | | | |
| H | 0.254149 | 0.178844 | -2.316264 |  | | | |
| H | 1.75416 | 1.113291 | -2.14722 |  | | | |
| H | -0.680959 | 2.349068 | -1.817346 |  | | | |
| H | -6.917918 | -2.223375 | 0.1062 |  | | | |
| H | -7.039083 | -0.914438 | -1.06247 |  | | | |
| H | -5.865304 | -2.221963 | -1.324531 |  | | | |
| H | -6.587603 | 0.806797 | 0.846806 |  | | | |
| H | -6.431336 | -0.493545 | 2.019977 |  | | | |
| H | -5.115114 | 0.675247 | 1.821665 |  | | | |
| H | -1.390111 | 1.627486 | 2.138844 |  | | | |
| H | -1.603225 | 3.028688 | 1.071156 |  | | | |
| H | 0.017107 | 2.591962 | 1.647592 |  | | | |
| H | 5.613228 | -2.229748 | 0.192705 |  | | | |
| H | 4.867997 | -2.367475 | 1.79836 |  | | | |
| H | 6.359523 | -1.434229 | 1.586329 |  | | | |
| H | 5.563951 | 0.09497 | -1.041844 |  | | | |
| H | 6.319274 | 0.829559 | 0.386116 |  | | | |
| H | 4.789616 | 1.478101 | -0.239946 |  | | | |
| H | -3.129204 | 0.344414 | 1.456226 |  | | | |
| H | -2.098677 | -0.981963 | 0.932179 |  | | | |
| H | -2.736972 | 1.519526 | -0.709813 |  | | | |
| H | -1.869395 | 0.119752 | -1.329635 |  | | | |
| H | 4.42549 | 0.106158 | 1.807657 |  | | | |
| H | 1.319354 | 3.382757 | -1.320999 |  | | | |

Key transitions, oscillator strengths, and rotatory strengths in the ECD of conformers 4*R*,6*S*,7*R*-**2 F** at B3LYP /6-311++G (d,p) level.

| Species | Excited State | *∆E(eV)^a^* | *λ(nm)^b^* | *f*^c^ | *R_vel_^d^* |
| --- | --- | --- | --- | --- | --- |
| 4*R*,6*S*,7*R*-**2 F** | 78->81 | 3.9141 | 316.76 | 0.0019 | -14.0502 |
|  | 80->81 | 4.3577 | 284.51 | 0.0014 | -1.3451 |
|  | 79->81 | 4.7886 | 258.91 | 0.201 | 47.1989 |
|  | 76->81 | 5.4708 | 226.63 | 0.0607 | -23.9251 |
|  | 80->82 | 5.5319 | 224.12 | 0.0017 | 7.4148 |
|  | 77->81 | 5.5764 | 222.34 | 0.0225 | -0.2303 |
|  | 80->83 | 5.8909 | 210.47 | 0.0053 | 1.7347 |
|  | 79->82 | 6.0499 | 204.93 | 0.0193 | -8.903 |
|  | 79->82 | 6.077 | 204.02 | 0.0032 | 0.1021 |
|  | 80->85 | 6.112 | 202.86 | 0.0483 | -16.8889 |
|  | 80->86 | 6.2217 | 199.28 | 0.0926 | 19.6236 |
|  | 78->82 | 6.2648 | 197.9 | 0.004 | -10.6069 |
|  | 79->83 | 6.3614 | 194.9 | 0.0104 | 2.7271 |
|  | 75->81 | 6.382 | 194.27 | 0.0006 | 0.9099 |
|  | 80->88 | 6.3966 | 193.83 | 0.0068 | -17.4723 |
|  | 80->87 | 6.4458 | 192.35 | 0.1304 | 2.2986 |
|  | 79->84 | 6.4697 | 191.64 | 0.0106 | -9.0495 |
|  | 79->85 | 6.5485 | 189.33 | 0.0155 | 13.6308 |
|  | 78->83 | 6.5557 | 189.12 | 0.0021 | -1.591 |
|  | 73->81 | 6.6318 | 186.95 | 0.1247 | -29.5386 |
|  | 80->90 | 6.6555 | 186.29 | 0.0079 | -11.1963 |
|  | 78->84 | 6.6568 | 186.25 | 0.0367 | -19.0484 |
|  | 80->89 | 6.6661 | 185.99 | 0.073 | -1.4207 |
|  | 79->86 | 6.6978 | 185.11 | 0.0024 | -0.8535 |
|  | 78->85 | 6.7327 | 184.15 | 0.0125 | -29.6386 |
|  | 80->91 | 6.7446 | 183.83 | 0.001 | -0.2118 |
|  | 79->87 | 6.7889 | 182.63 | 0.0032 | 8.3437 |
|  | 74->81 | 6.8032 | 182.24 | 0.0254 | 7.3183 |
|  | 72->81 | 6.8238 | 181.69 | 0.0068 | 13.7303 |
|  | 77->82 | 6.8578 | 180.79 | 0.0003 | -1.8256 |
|  | 80->94 | 6.8689 | 180.5 | 0.0197 | 27.9916 |
|  | 78->86 | 6.8807 | 180.19 | 0.0145 | -23.6273 |
|  | 79->88 | 6.8878 | 180.01 | 0.0035 | -4.9209 |
|  | 78->86 | 6.9175 | 179.23 | 0.0178 | -3.1186 |
|  | 80->93 | 6.9363 | 178.75 | 0.004 | -4.0711 |
|  | 78->87 | 6.9806 | 177.61 | 0.0044 | -7.9641 |
|  | 80->94 | 7.0198 | 176.62 | 0.0563 | 11.1067 |
|  | 80->94 | 7.0336 | 176.27 | 0.0079 | -16.6268 |
|  | 71->81 | 7.0475 | 175.93 | 0.0186 | -7.5874 |
|  | 78->88 | 7.0782 | 175.16 | 0.0131 | -5.9352 |

*^a^*Excitation energy. *^b^*Wavelength. *^c^*Oscillator strength. *^d^*Rotatory strength in velocity form (10^-40^cgs.).

**^13^C NMR calculation section**

In order to confirm the relative configurations of compounds **1** and **2**, ^13^C NMR calculations were employed and the absolute structures of which were established by calculated ECD spectra analyses.


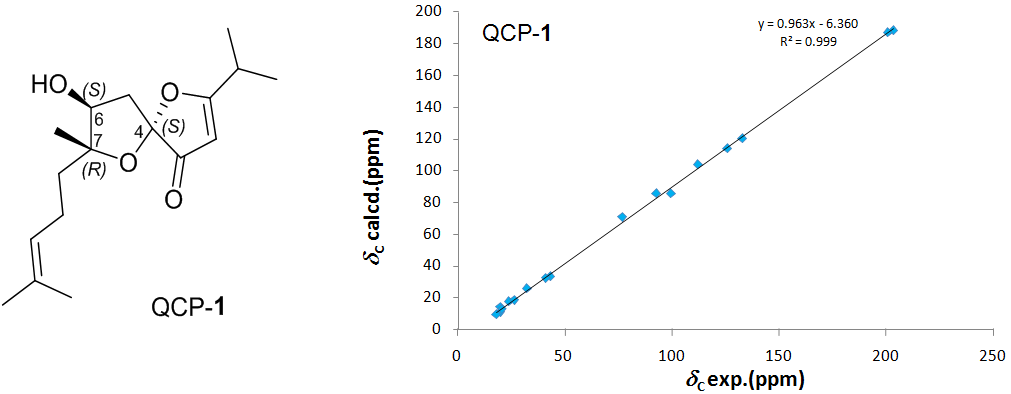


Linear correlation between the scaled calculated and experimental ^13^C NMR chemical shifts for QCP-**1**.

Optimized coordinates (Å) of compound QCP-**1** in the methanol solution at B3LYP/6-31G* level.

| C | 0.04165 | 2.47573 | -1.00251 |
| --- | --- | --- | --- |
| C | -0.7149 | 1.69253 | 0.11007 |
| O | 0.33629 | 0.81713 | 0.65241 |
| C | 1.37346 | 0.63283 | -0.26798 |
| C | 1.00589 | 1.40545 | -1.54057 |
| C | 2.71714 | 1.04609 | 0.37791 |
| C | 3.50335 | -0.14801 | 0.46828 |
| O | 2.95221 | 2.21252 | 0.70439 |
| O | 0.73948 | 3.61481 | -0.52516 |
| C | 2.76874 | -1.15953 | -0.08401 |
| O | 1.54593 | -0.78645 | -0.53554 |
| C | 3.08701 | -2.61174 | -0.27045 |
| C | 2.10639 | -3.47788 | 0.55269 |
| C | 4.54882 | -2.94119 | 0.05472 |
| C | -1.85147 | 0.83435 | -0.48581 |
| C | -2.46206 | -0.20144 | 0.48081 |
| C | -3.407 | -1.13619 | -0.22842 |
| C | -4.72341 | -1.31404 | -0.03074 |
| C | -5.49906 | -2.31055 | -0.8614 |
| C | -5.55267 | -0.58309 | 0.99768 |
| C | -1.18853 | 2.56776 | 1.26428 |
| H | -0.64948 | 2.83565 | -1.76956 |
| H | 0.51639 | 0.7273 | -2.24388 |
| H | 1.87186 | 1.84951 | -2.03648 |
| H | 4.49667 | -0.23315 | 0.88337 |
| H | 1.4968 | 3.29462 | 0.01422 |
| H | 2.90211 | -2.82454 | -1.33334 |
| H | 2.24623 | -3.30619 | 1.62578 |
| H | 1.06692 | -3.25345 | 0.29835 |
| H | 2.29296 | -4.53677 | 0.34787 |
| H | 4.76963 | -2.763 | 1.11326 |
| H | 5.2412 | -2.34352 | -0.54701 |
| H | 4.74283 | -3.99784 | -0.15377 |
| H | -1.47886 | 0.30389 | -1.37103 |
| H | -2.63861 | 1.51083 | -0.84304 |
| H | -2.95917 | 0.30746 | 1.31164 |
| H | -1.63784 | -0.78317 | 0.91595 |
| H | -2.93719 | -1.73391 | -1.013 |
| H | -4.86163 | -2.81719 | -1.59314 |
| H | -6.31894 | -1.81934 | -1.4044 |
| H | -5.96563 | -3.07687 | -0.22621 |
| H | -6.387 | -0.05523 | 0.51515 |
| H | -6.00551 | -1.29247 | 1.70454 |
| H | -4.98351 | 0.1482 | 1.57637 |
| H | -1.99425 | 3.22952 | 0.92871 |
| H | -1.56793 | 1.95403 | 2.08605 |
| H | -0.36945 | 3.18499 | 1.63742 |

Deviations between the calculated and experimental ^13^C NMR chemical shifts for QCP-**1**.

| **C** | **exp.** | QCP-**1** | | | |
| --- | --- | --- | --- | --- | --- |
| **calc.** | **scal.calc.** | ***∆δ*** | **|*∆δ*|** |
| 1 | 200.8682 | 187.3857 | 201.1691 | 0.3009 | 0.3009 |
| 2 | 99.3011 | 85.7544 | 95.6440 | -3.6571 | 3.6571 |
| 3 | 203.3114 | 188.5308 | 201.1691 | -2.1423 | 2.1423 |
| 4 | 112.0611 | 103.8156 | 114.3972 | 2.3361 | 2.3361 |
| 5 | 43.0615 | 33.2947 | 41.1743 | -1.8872 | 1.8872 |
| 6 | 76.3907 | 70.4488 | 79.7519 | 3.3612 | 3.3612 |
| 7 | 92.5793 | 86.0396 | 95.9401 | 3.3608 | 3.3608 |
| 8 | 41.2714 | 32.3084 | 40.1502 | -1.1212 | 1.1212 |
| 9 | 23.7765 | 17.6551 | 24.9355 | 1.159 | 1.159 |
| 10 | 125.4258 | 113.8746 | 124.8416 | -0.5842 | 0.5842 |
| 11 | 132.6904 | 120.2971 | 131.5101 | -1.1803 | 1.1803 |
| 12 | 17.8308 | 9.7346 | 16.7116 | -1.1192 | 1.1192 |
| 13 | 26.0024 | 18.6096 | 25.9266 | -0.0758 | 0.0758 |
| 14 | 32.0246 | 25.78 | 33.3717 | 1.3471 | 1.3471 |
| 15 | 19.6943 | 10.87 | 17.8905 | -1.8038 | 1.8038 |
| 16 | 19.7081 | 13.7296 | 20.8596 | 1.1515 | 1.1515 |
| 17 | 20.3102 | 12.5777 | 19.6636 | -0.6466 | 0.6466 |
|  |  |  |  | AveDev | 1.5337 |
|  |  |  |  | MaxDev | 3.6571 |


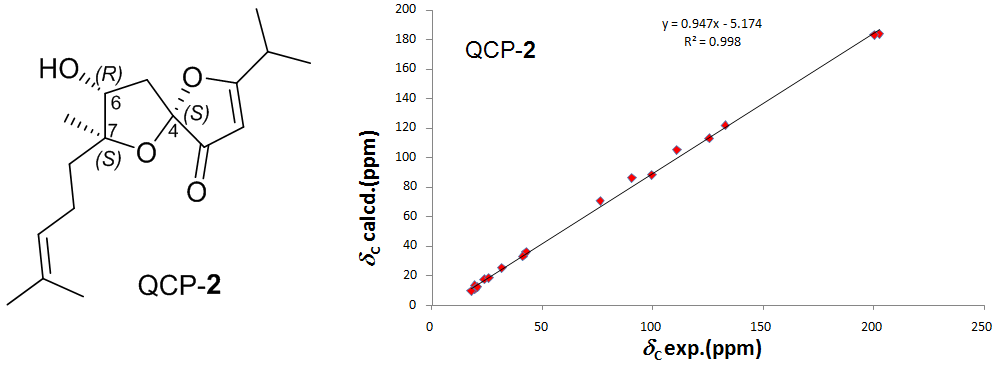


Linear correlation between the scaled calculated and experimental 13C NMR chemical shifts for QCP-**2**.

Optimized coordinates (Å) of compound QCP-**2** in the methanol solution at B3LYP/6-31G* level.

| C | 0.06631 | 2.05473 | -1.04278 |
| --- | --- | --- | --- |
| C | -0.7041 | 1.64012 | 0.24631 |
| O | -0.04575 | 0.38025 | 0.65671 |
| C | 0.86747 | -0.0438 | -0.29126 |
| C | 0.53924 | 0.69597 | -1.58445 |
| C | 0.94417 | -1.58792 | -0.37154 |
| C | 2.30402 | -1.93388 | -0.04584 |
| O | -0.01804 | -2.28425 | -0.67662 |
| O | 1.15533 | 2.94245 | -0.8185 |
| C | 2.98104 | -0.78354 | 0.23179 |
| O | 2.23037 | 0.34199 | 0.13738 |
| C | 4.41588 | -0.55456 | 0.60237 |
| C | 5.13587 | 0.22826 | -0.51903 |
| C | 5.14829 | -1.8575 | 0.94465 |
| C | -2.17508 | 1.31897 | -0.08811 |
| C | -2.92715 | 0.50728 | 0.98745 |
| C | -4.34003 | 0.18305 | 0.57181 |
| C | -4.80376 | -0.97806 | 0.08124 |
| C | -6.26142 | -1.13873 | -0.28265 |
| C | -3.95941 | -2.2093 | -0.15084 |
| C | -0.56286 | 2.62805 | 1.39859 |
| H | -0.5986 | 2.57414 | -1.73702 |
| H | -0.26838 | 0.15935 | -2.09151 |
| H | 1.39021 | 0.7811 | -2.26278 |
| H | 2.70872 | -2.9346 | -0.00819 |
| H | 1.85382 | 2.44712 | -0.35633 |
| H | 4.39748 | 0.08852 | 1.49399 |
| H | 4.62279 | 1.1675 | -0.74422 |
| H | 6.15863 | 0.46067 | -0.20578 |
| H | 5.18564 | -0.36819 | -1.43692 |
| H | 6.16977 | -1.63279 | 1.267 |
| H | 5.21033 | -2.51804 | 0.07237 |
| H | 4.6487 | -2.40103 | 1.75312 |
| H | -2.21501 | 0.75561 | -1.02856 |
| H | -2.6961 | 2.2673 | -0.27471 |
| H | -2.95858 | 1.08061 | 1.92337 |
| H | -2.35727 | -0.40132 | 1.19803 |
| H | -5.05058 | 1.00581 | 0.6721 |
| H | -6.37866 | -1.39508 | -1.34506 |
| H | -6.7213 | -1.96027 | 0.28499 |
| H | -6.83558 | -0.22744 | -0.08633 |
| H | -4.10211 | -2.57904 | -1.17554 |
| H | -2.88946 | -2.04016 | -0.00747 |
| H | -4.2696 | -3.02705 | 0.51542 |
| H | -1.03016 | 3.58294 | 1.13616 |
| H | -1.04962 | 2.24223 | 2.29884 |
| H | 0.48876 | 2.81372 | 1.62699 |

Deviations between the calculated and experimental ^13^C NMR chemical shifts for QCP-**2**.

| **C** | **exp.** | QCP-**2** | | | |
| --- | --- | --- | --- | --- | --- |
| **calc.** | **scal.calc.** | ***∆δ*** | **|*∆δ*|** |
| 1 | 200.3932 | 182.6764 | 198.2176 | -2.1756 | 2.1756 |
| 2 | 99.7121 | 87.9857 | 98.3013 | -1.4108 | 1.4108 |
| 3 | 202.4681 | 183.3801 | 198.9601 | -3.508 | 3.508 |
| 4 | 111.0439 | 105.1257 | 116.3871 | 5.3432 | 5.3432 |
| 5 | 42.6236 | 35.7779 | 43.2123 | 0.5887 | 0.5887 |
| 6 | 76.0608 | 70.665 | 80.0247 | 3.9639 | 3.9639 |
| 7 | 90.9602 | 85.7878 | 95.9821 | 5.0219 | 5.0219 |
| 8 | 41.603 | 33.3449 | 40.6450 | -0.958 | 0.958 |
| 9 | 23.6256 | 17.2843 | 23.6981 | 0.0725 | 0.0725 |
| 10 | 125.4476 | 112.8344 | 124.5213 | -0.9263 | 0.9263 |
| 11 | 132.672 | 122.0874 | 134.2849 | 1.6129 | 1.6129 |
| 12 | 17.8566 | 9.5212 | 15.5066 | -2.35 | 2.35 |
| 13 | 25.9879 | 18.4417 | 24.9194 | -1.0685 | 1.0685 |
| 14 | 32.0058 | 25.3466 | 32.2053 | 0.1995 | 0.1995 |
| 15 | 19.6211 | 10.8646 | 16.9241 | -2.697 | 2.697 |
| 16 | 19.7274 | 13.7046 | 19.9209 | 0.1935 | 0.1935 |
| 17 | 20.4985 | 12.4281 | 18.5739 | -1.9246 | 1.9246 |
|  |  |  |  | AveDev | 2.0134 |
|  |  |  |  | MaxDev | 5.3432 |

HRESIMS spectrum of (±)-Japonone A [(±)-**1**]

^1^H NMR spectrum of (±)-Japonone A [(±)-**1**, in CD_3_OD]


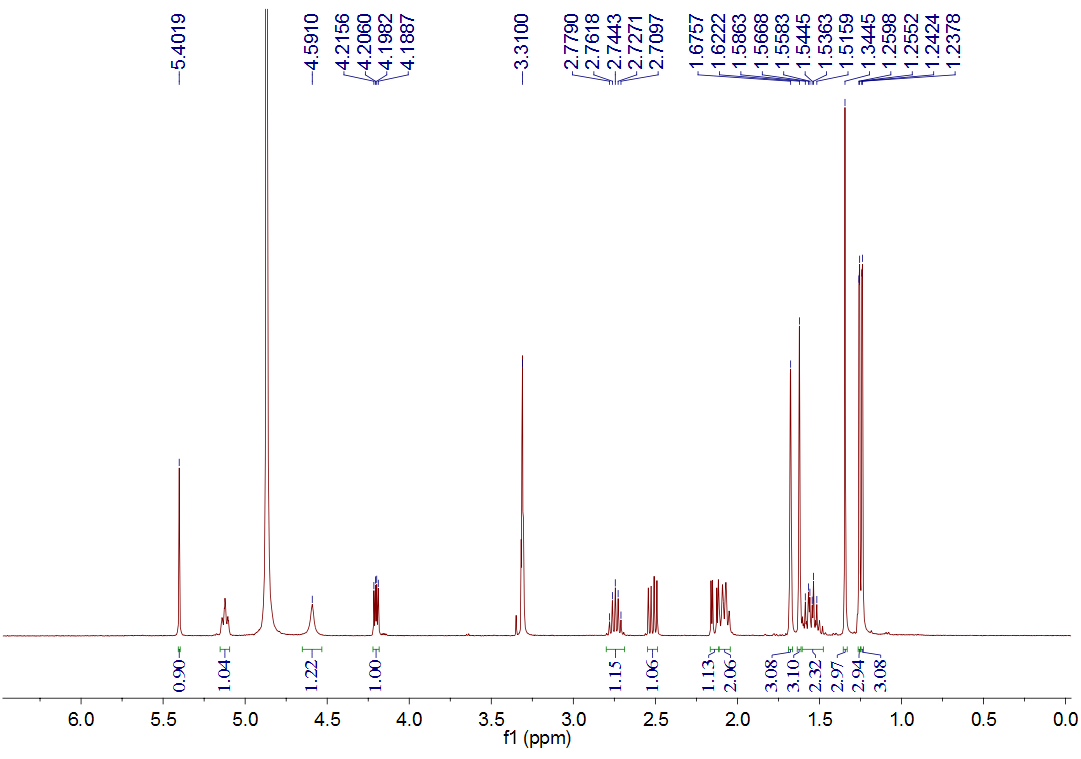


^13^C NMR and DEPT135 spectra of (±)-Japonone A [(±)-**1**, in CD_3_OD]


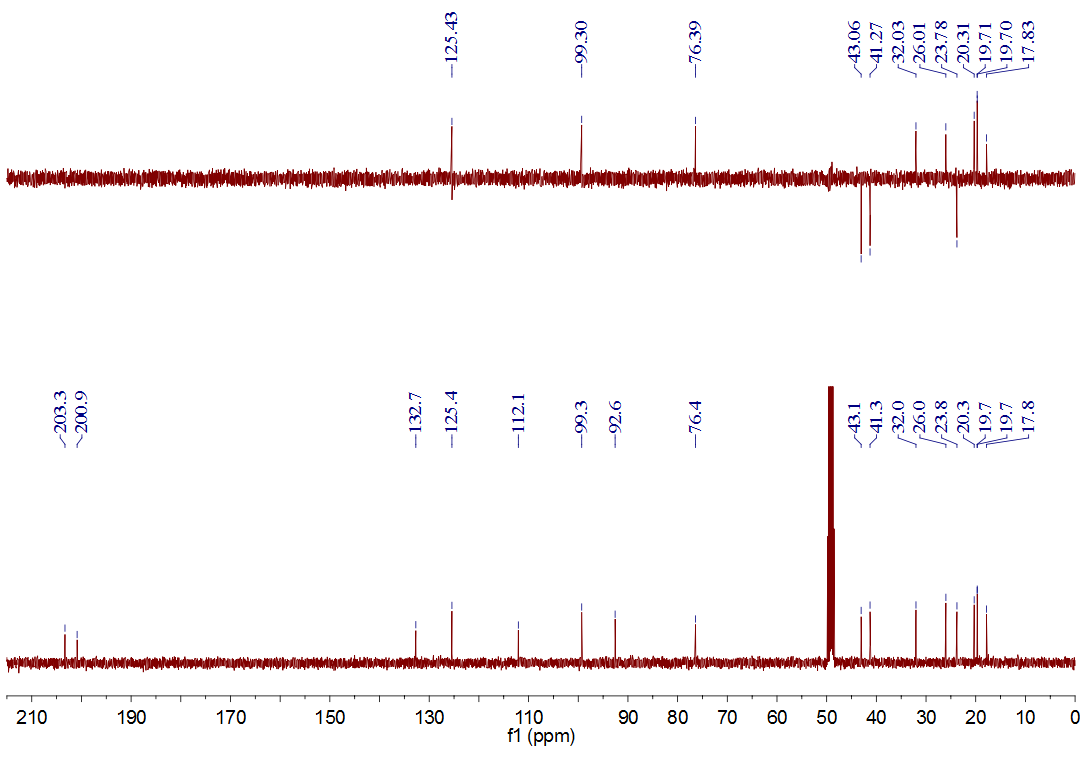


HSQC spectrum of (±)-Japonone A [(±)-**1**, in CD_3_OD]


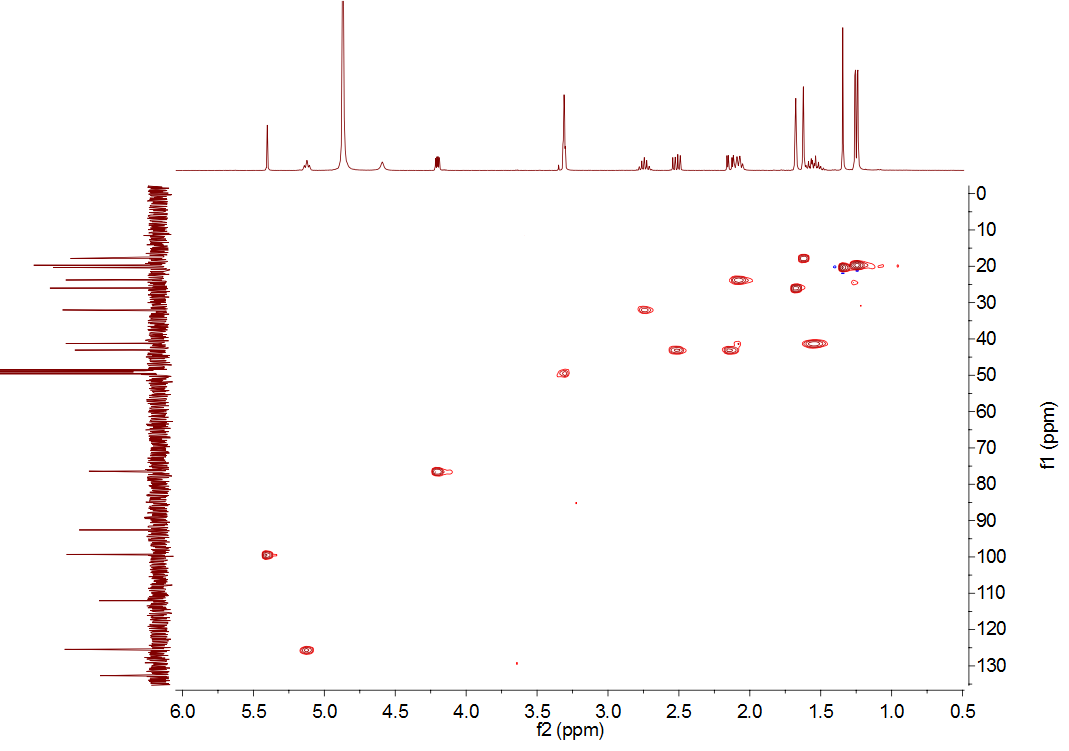


HMBC spectrum of (±)-Japonone A [(±)-**1**, in CD_3_OD]


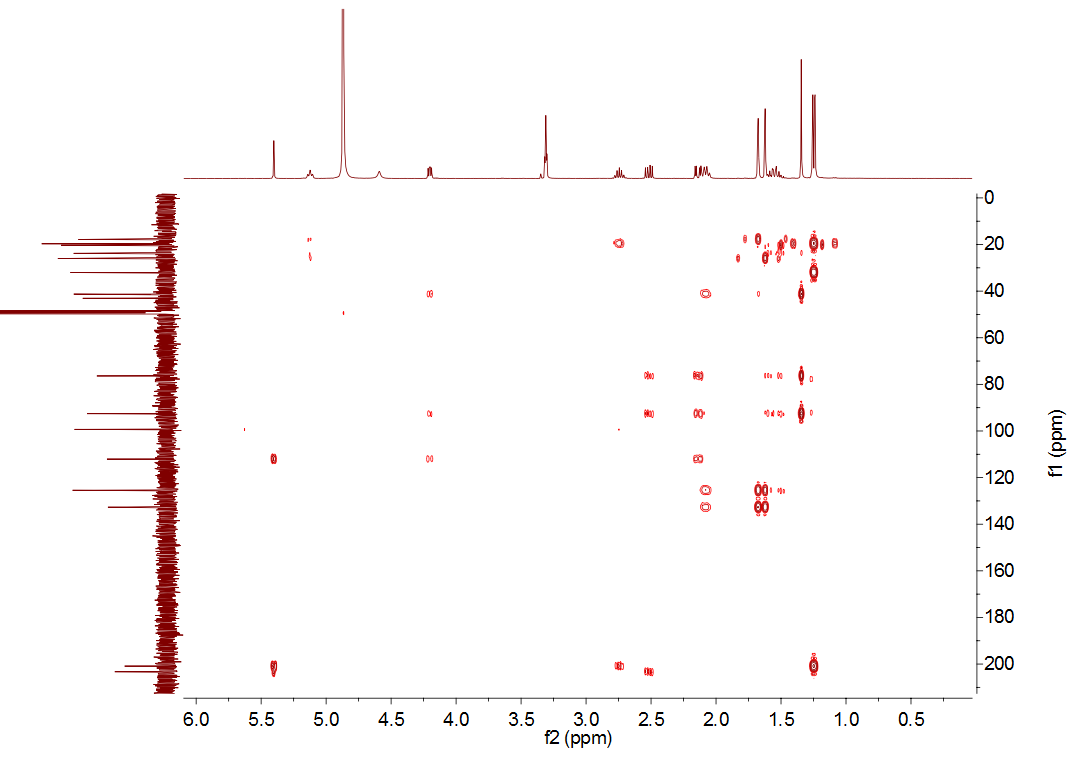


^1^H–^1^H COSY spectrum of (±)-Japonone A [(±)-**1**, in CD_3_OD]


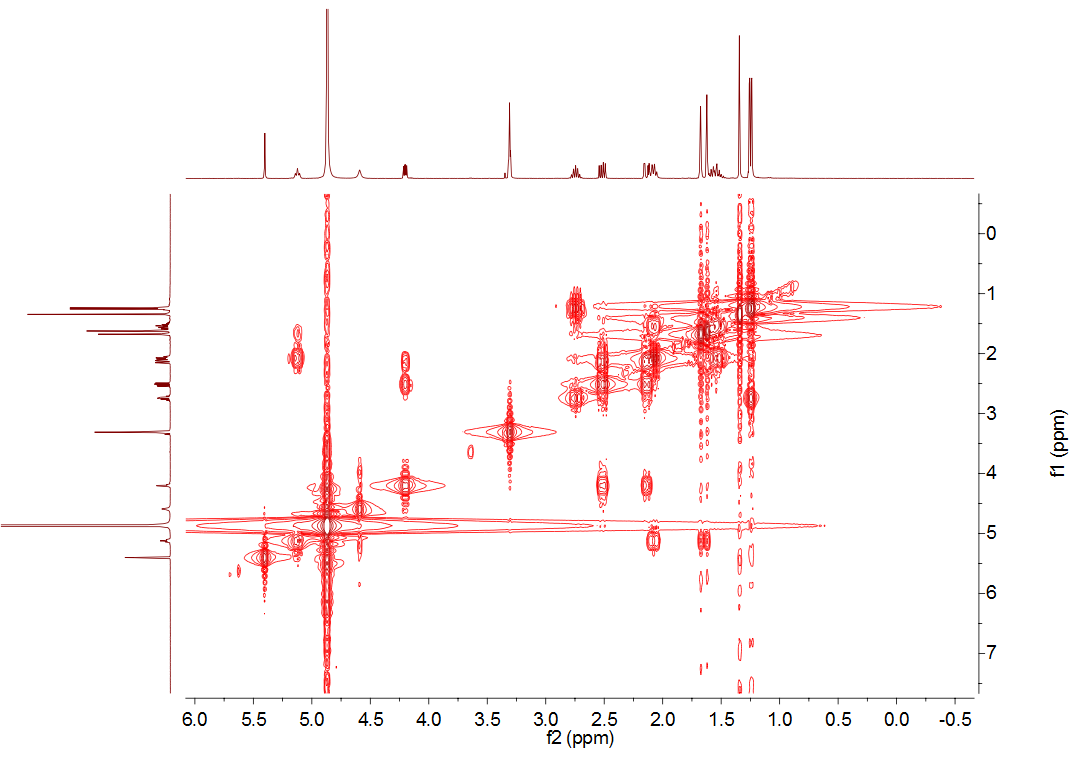


NOESY spectrum of (±)-Japonone A [(±)-**1**, in CD_3_OD]


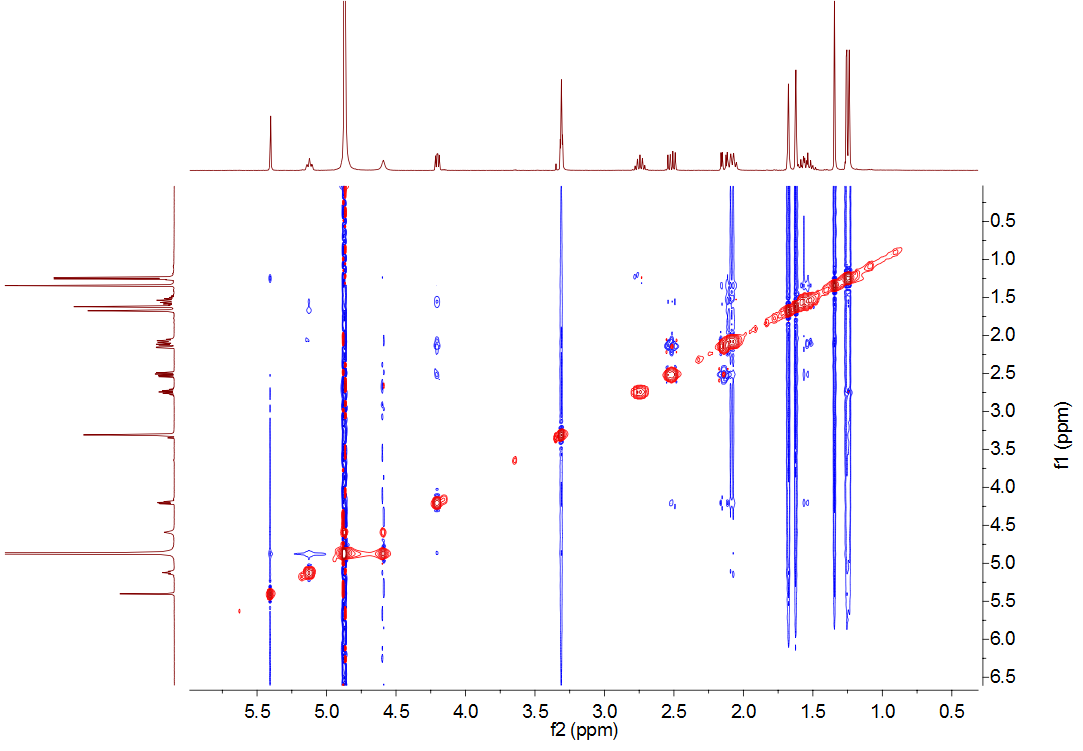


# UV spectrum of (±)-Japonone A [(±)-1]


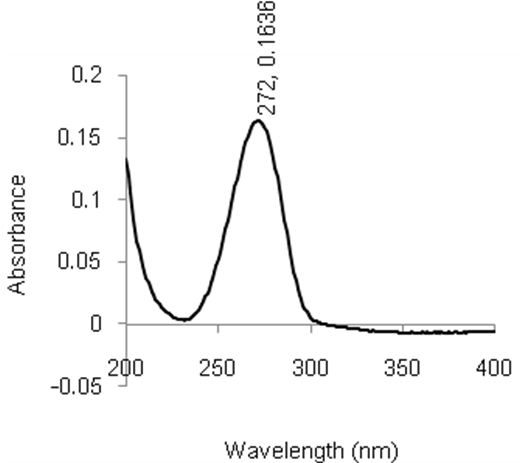


IR spectrum of (±)-Japonone A [(±)-**1**]


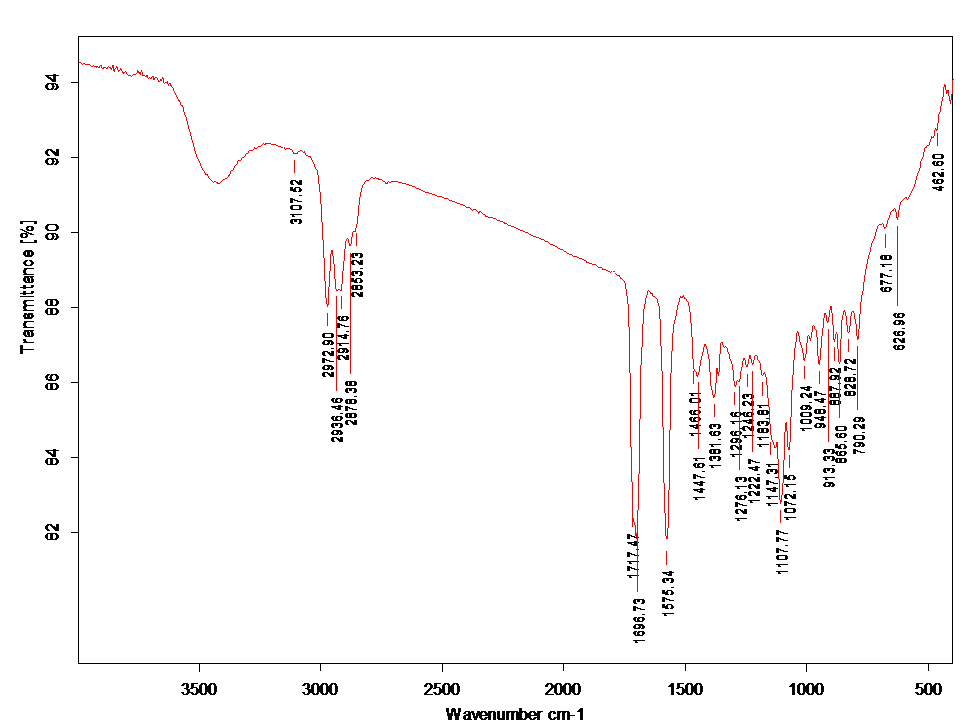


HRESIMS spectrum of (±)-Japonone B [(±)-**2**]


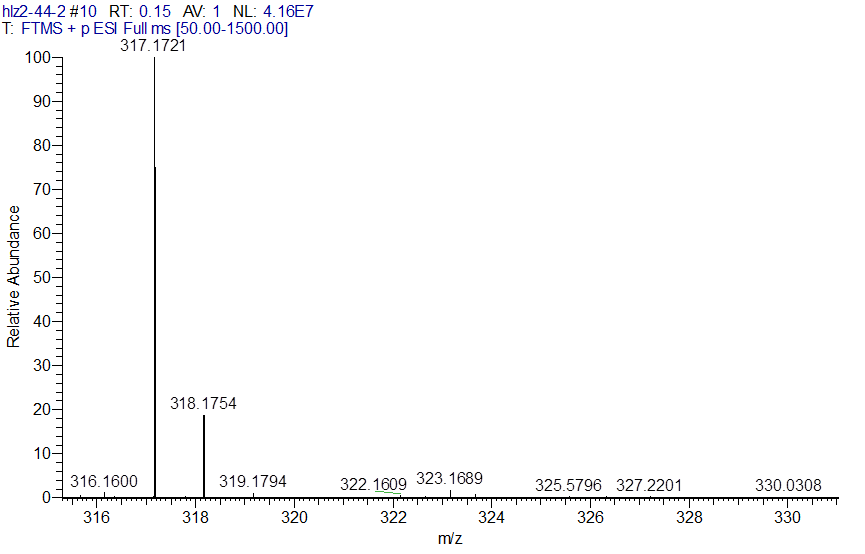


^1^H NMR spectrum of (±)-Japonone B [(±)-**2**, in CD_3_OD]


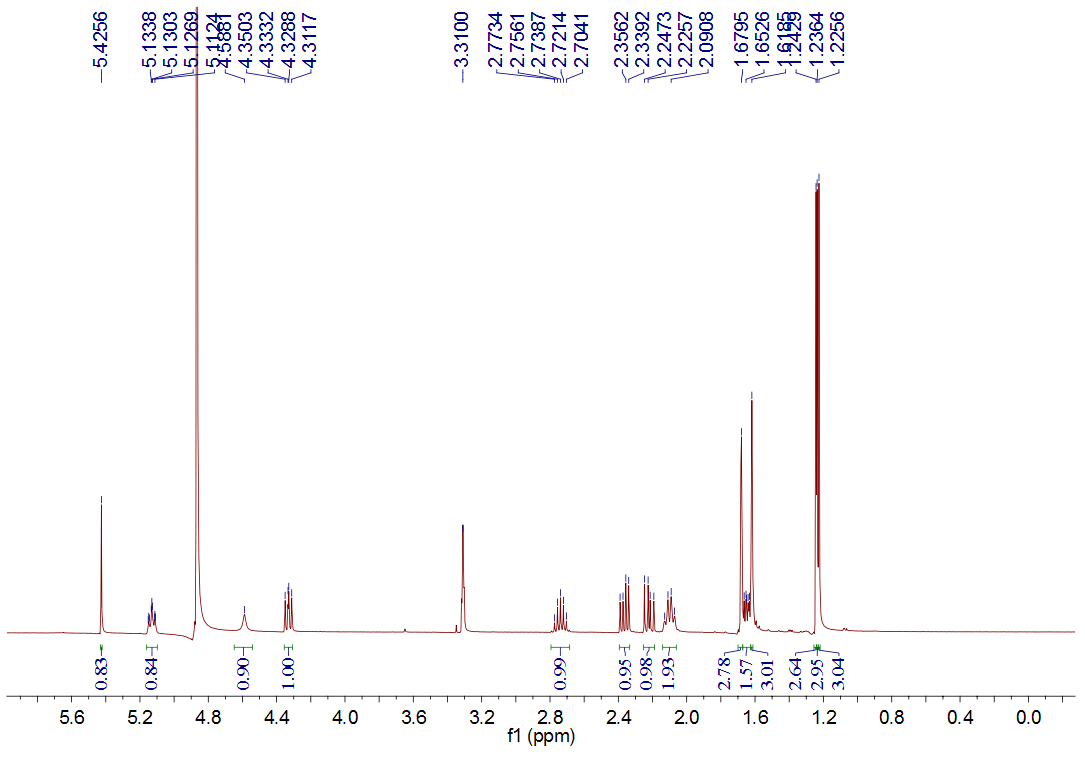


^13^C NMR and DEPT spectra of (±)-Japonone B [(±)-**2**, in CD_3_OD]


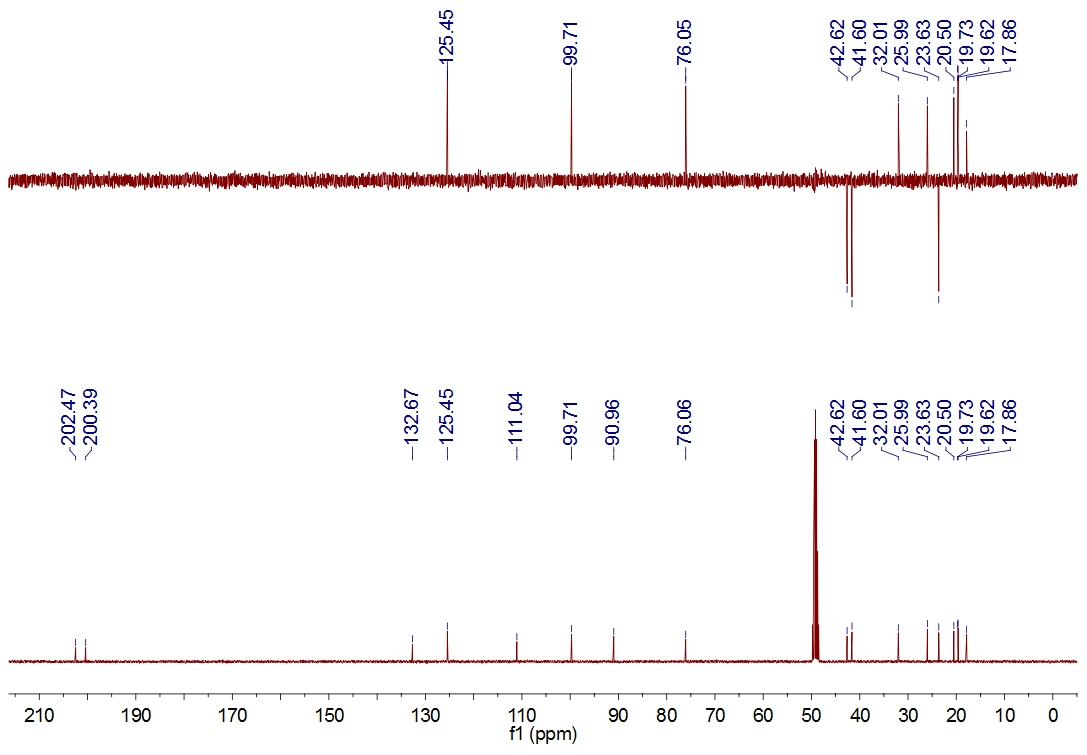


HSQC spectrum of (±)-Japonone B [(±)-**2**, in CD_3_OD]


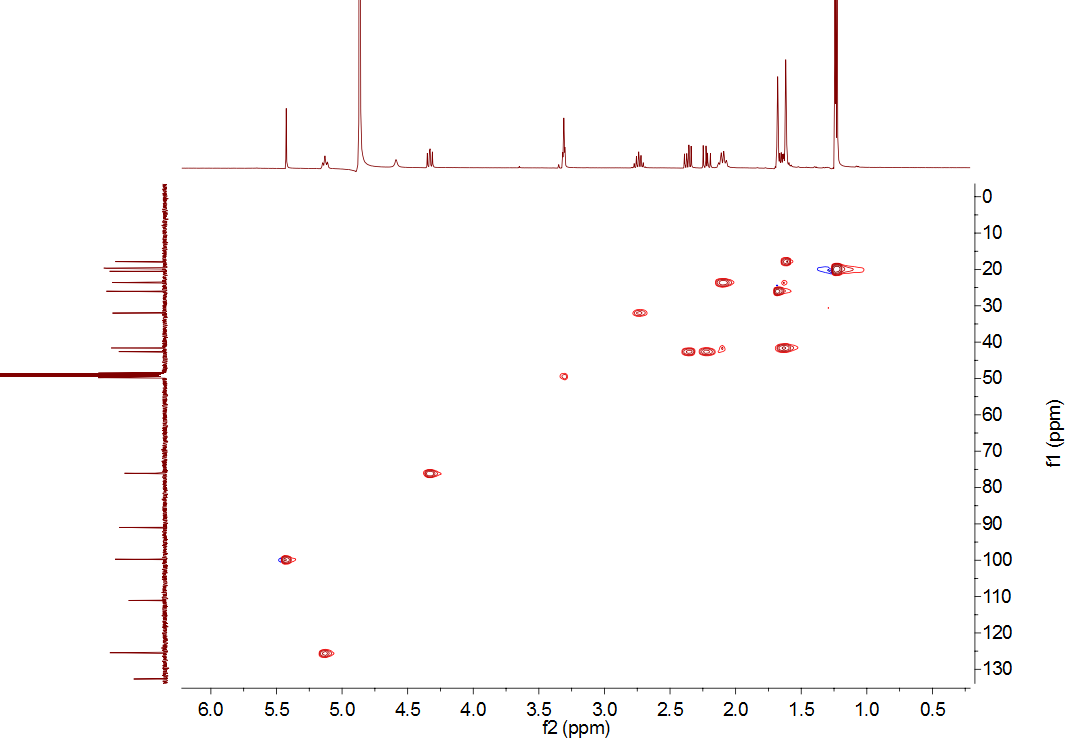


HMBC spectrum of (±)-Japonone B [(±)-**2**, in CD_3_OD]


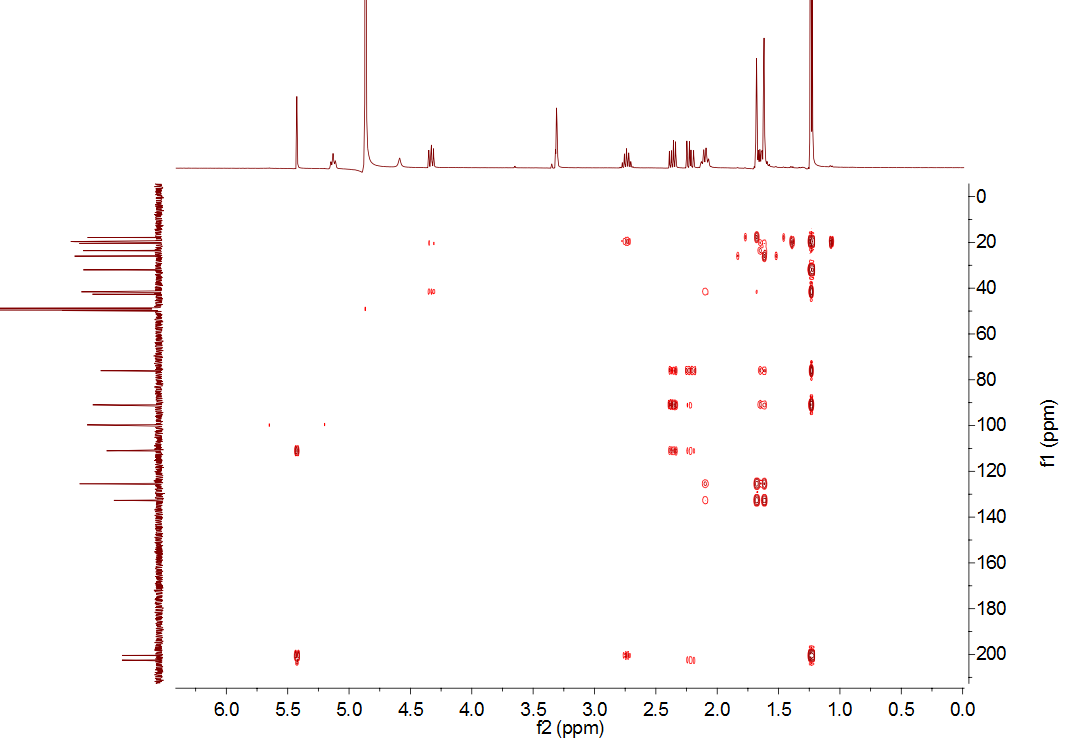


^1^H–^1^H COSY spectrum of (±)-Japonone B [(±)-**2**, in CD_3_OD]


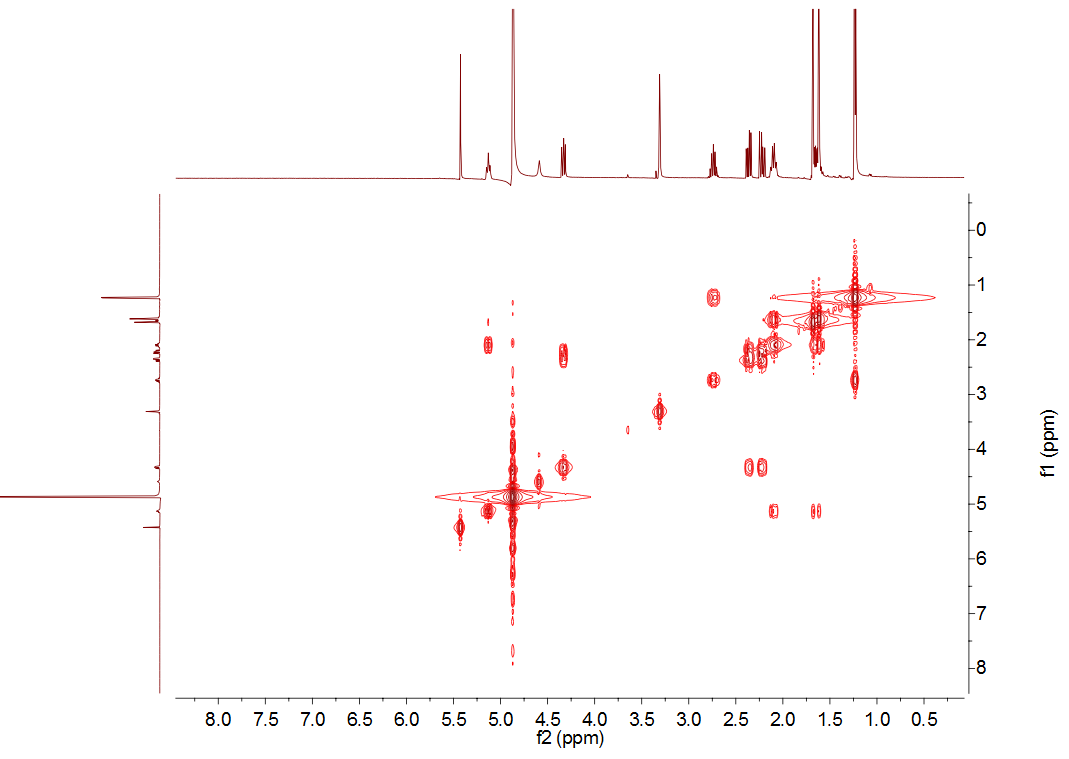


NOESY spectrum of (±)-Japonone B [(±)-**2**, in CD_3_OD]


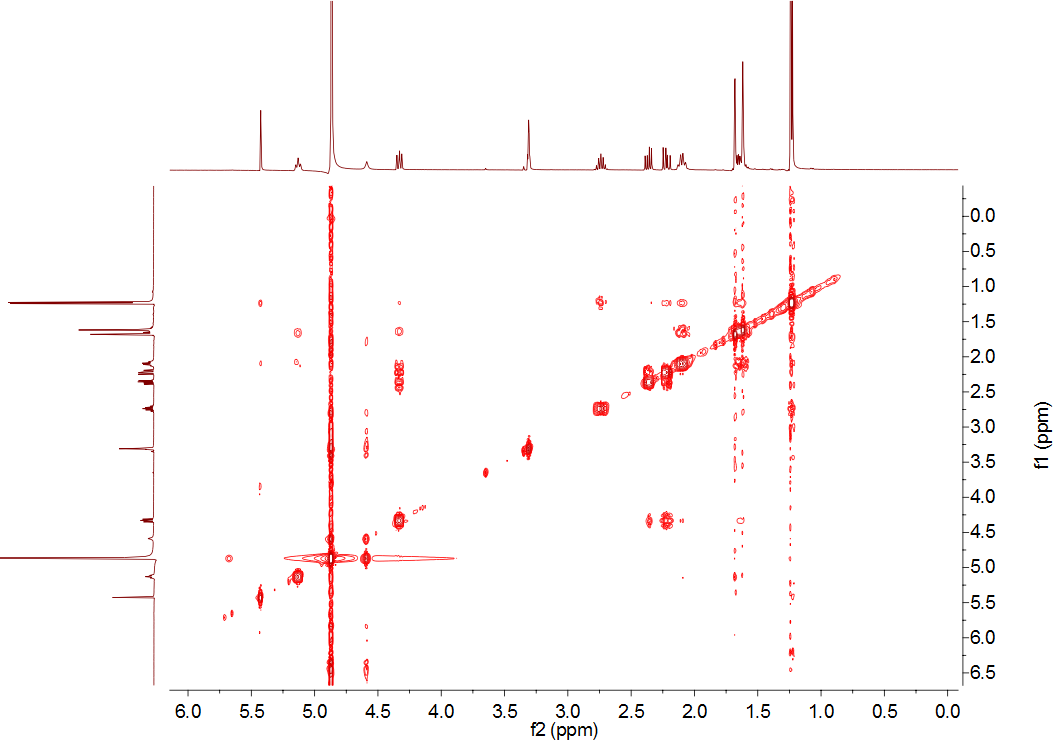


# UV spectrum of (±)-Japonone B [(±)-2]


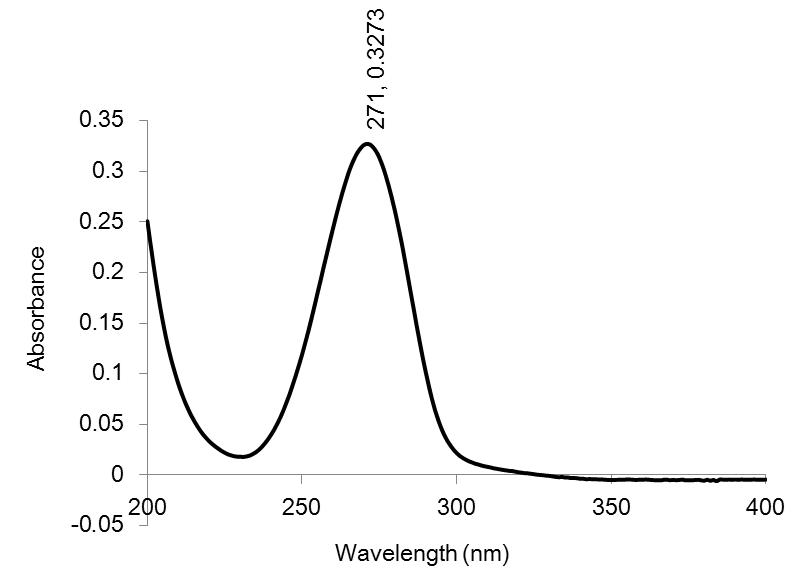


IR spectrum of (±)-Japonone B [(±)-**2**]


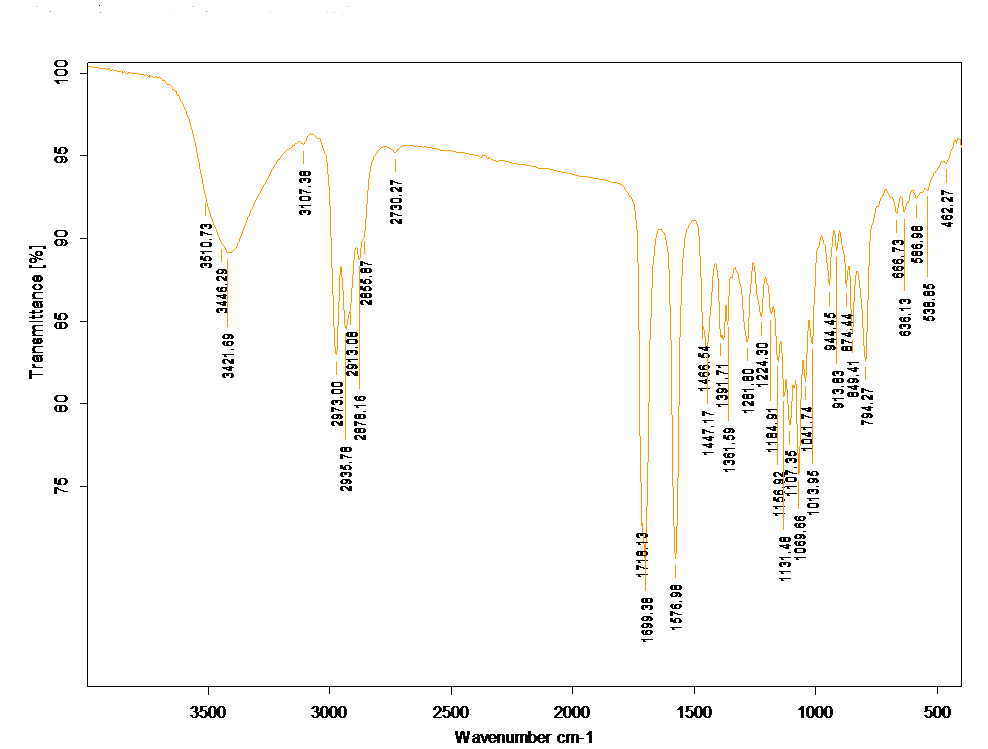


HRESIMS spectrum of compound (−)-**1a**

^1^H NMR spectrum of compound (−)-**1a** (in CD_3_OD)

**
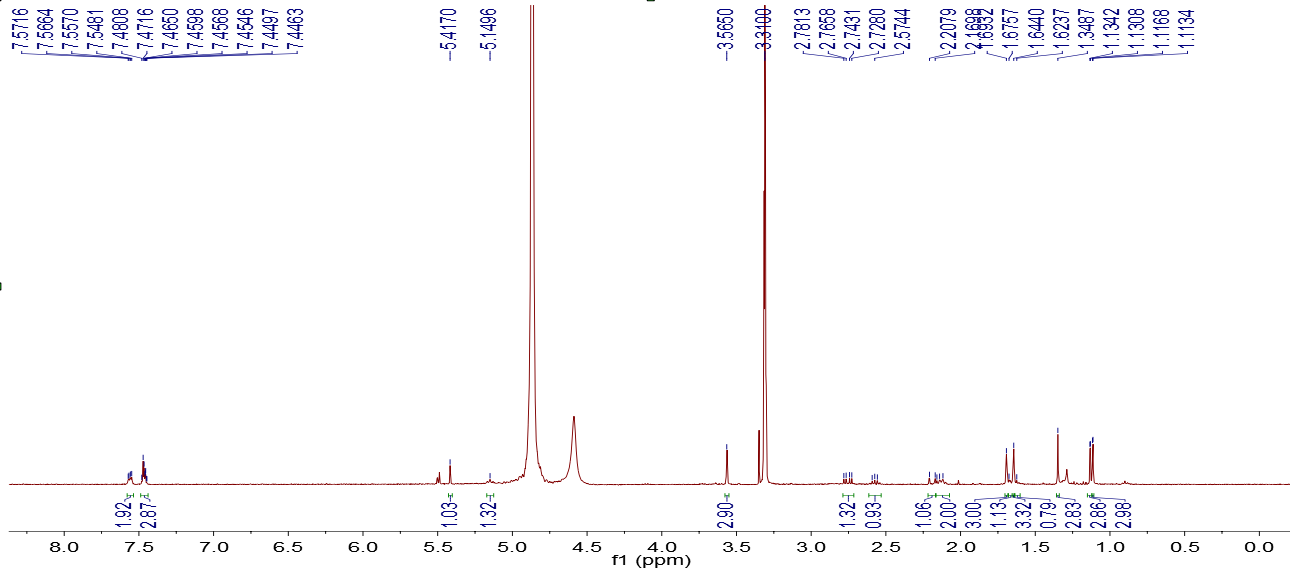
**

HRESIMS spectrum of compound (−)-**1b**

^1^H NMR spectrum of compound (−)-**1b** (in CD_3_OD)


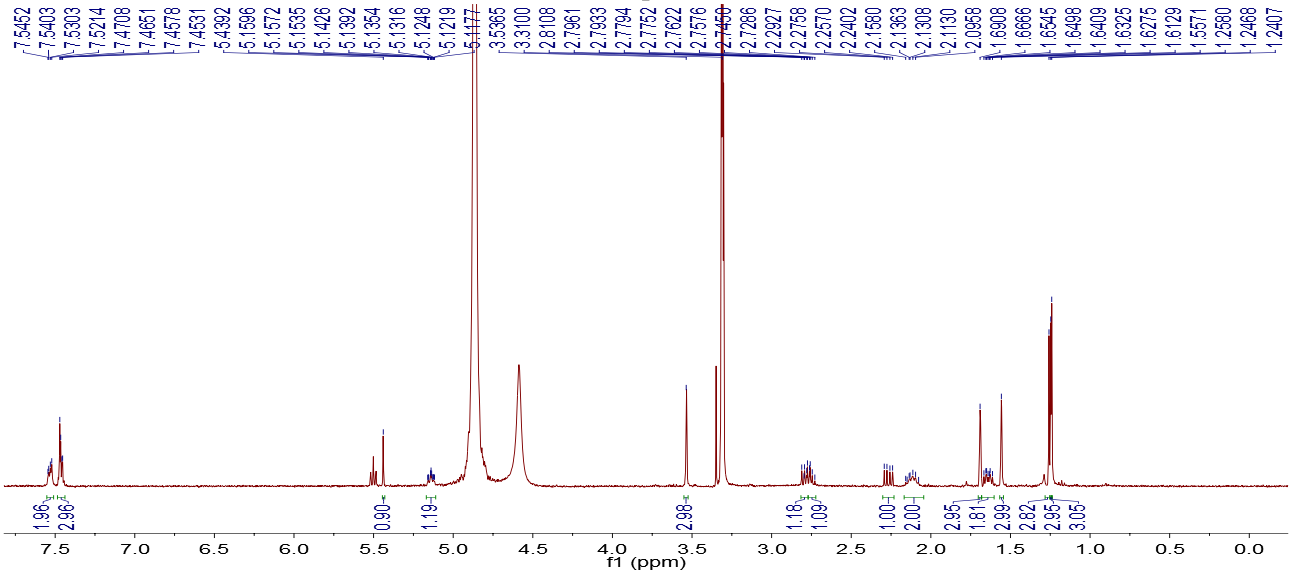


HRESIMS spectrum of compound (−)-**2a**

^1^H NMR spectrum of compound (−)-**2a** (in CD_3_OD)

**
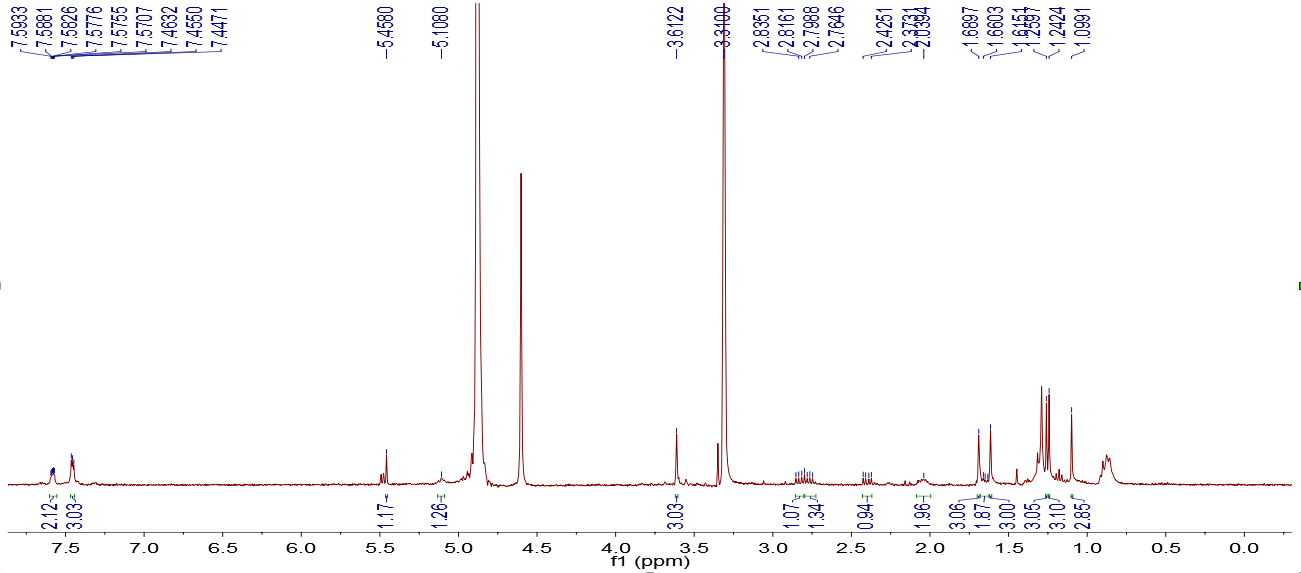
**

HRESIMS spectrum of compound (−)-**2b**

^1^H NMR spectrum of compound (−)-**2b** (in CD_3_OD)

**
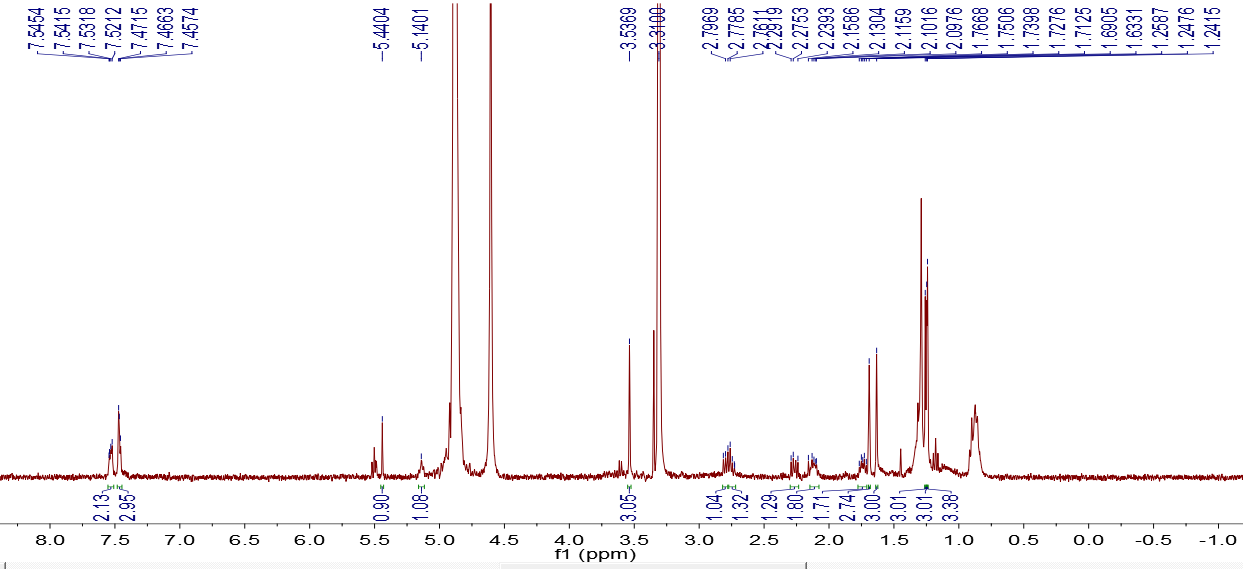
**
